# Synthesis and Reactivity of Heteroleptic U^4+^ Alkyl, Benzyl, and Hydride Imidophosphorane Complexes

**DOI:** 10.1021/acs.inorgchem.6c01434

**Published:** 2026-06-17

**Authors:** Haruko Tateyama, Maximilian G. Bernbeck, Tyler-Rayne Nero, Grant R. Wilkinson, Kaitlyn S. Engle, Gabriel M. Betts, Henry S. La Pierre

**Affiliations:** † School of Chemistry and Biochemistry, 1372 Georgia Institute of Technology, Atlanta, Georgia 30332-0400, United States; ‡ Nuclear and Radiological Engineering Program, Georgia Institute of Technology, Atlanta, Georgia 30332-0400, United States; ∥ Physical Sciences Division, Pacific Northwest National Laboratory, Richland, Washington 99352, United States

## Abstract

A series of heteroleptic U^4+^ benzyl, neopentyl, and methyl complexes supported by the imidophosphorane ligand, [N = P­(*N*,*N*′-di*tert*-butylethylenediamide)­(diethylamide)]^1–^ (NP*), were synthesized from the monoiodide precursor, [UI­(NP*)_3_]. These heteroleptic complexes were synthesized through the selective formation of [UI­(NP*)_3_] under transmetalation conditions in the reaction between [UI_4_(1,4-dioxane)_2_] and K­[NP*]. Formation of the homoleptic complex [U­(NP*)_4_] was not observed even in the presence of excess K­[NP*]. The oxidation and hydrogenolysis reactivity of the neopentyl complex, [U­(Npt)­(NP*)_3_] (Npt = neopentyl) was explored. While cyclic voltammetry indicates a potentially isolable U^5+^ alkyl cation, chemical oxidation of the neopentyl complex results in the isolation of a cationic U^4+^ complex with a bound diethyl ether in the primary coordination sphere, [U^4+^(NP*))_3_(Et_2_O)]­[(BArF_24_)] (BArF_24_ = tetrakis­(3,5-bis­(trifluoromethyl)­phenyl)­borate). Notably, hydrogenolysis of [U­(Npt)­(NP*)_3_] with H_2_ gas at −20 °C results in the formation of a terminal hydride intermediate confirmed by in situ NMR spectroscopy and deuterium labeling with D_2_. The connectivity and structural parameters of this hydride intermediate, [UH­(NP*)_3_], which rapidly thermally decomposes to the homoleptic complex, [U­(NP*)_4_], can be confirmed by single-crystal X-ray diffraction studies of a crystal grown by chilling the reaction mixture. The identity of [U­(NP*)_4_] was confirmed by its direct, bulk synthesis from [U­(Me)­(NP*)_3_] and HNP* in a protonolysis reaction.

## Graphical abstract

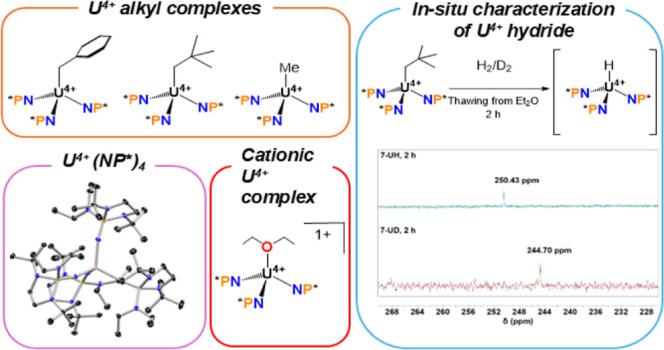

## Introduction

The search for volatile compounds of uranium to facilitate isotope separation drove initial interest in uranium alkyl complexes.^1,2^ However, these initial studies contended with the thermal instability of U–C σ bonds in homoleptic complexes.^3–10^ While homoleptic alkyl complexes have remained important and illuminating targets for the synthetic community, a range of supporting ligands have been developed and/or examined for their ability to stabilize alkyl complexes spanning U^3+^ to U^6+^. This creativity in approaches include the examples of cyclopentadienyl (Cp) ligands to fully saturate the coordination sphere around the uranium and protect the complex from undergoing β-hydrogen elimination and, in turn, isolate the first uranium alkyl complexes, Cp_3_UR (R includes methyl, allyl, neopentyl).^2,11,12^ Similar to its transition-metal congeners, alkyl complexes of uranium have been demonstrated as precursors to hydrides via hydrogenolysis.^13–16^ Hydrides of tetravalent uranium are known; however terminal hydrides (especially mononuclear hydrides) remain comparatively elusive due to their high reactivity.^14–21^

Imidophosphorane complexes have demonstrated the ability to stabilize high oxidation state f-element ions in the lanthanides, and in the actinides such as uranium, neptunium, and plutonium.^22–30^ Recently, we reported methodologies to access heteroleptic, imidophosphorane complexes of Ce^4+^ and demonstrated that the imidophosporane ligand system can stabilize seemingly incompatible combination of highly reducing alkyl ligands and highly oxidizing cerium metal centers.^31^ The high stability afforded by the imidophosphorane ligands to the Ce^4+^–alkyl bond presents opportunities to extend this chemistry in to the actinides, such as its redox congener Pu^4+^. Nonetheless, foundational chemistry of U^4+^ must be developed first in order to mitigate the risks and challenges of working with transuranic elements. We hypothesized that the NP* ligand, which has previously allowed for the isolation of high oxidation state lanthanide complexes, [Ln^4+^(NP*)_4_ ]­(Ln = Ce, Pr, Tb)^22,32,33^ would serve as a candidate ligand to access the heteroleptic, monoiodide complex of U^4+^, which would allow for further alkylation parallel to the Ce^4+^-alkyl complexes supported by imidophosphorane ligands.^31^ Given the high stability provided by the ligand coupled with the tunability of the ligand by varying the substituents for electronic and steric effects, heteroleptic imidophosphorane complexes present a robust platform for studying high oxidation state f-element systems bearing reactive, reducing ligands such as alkyls and hydrides.

Herein, we report the development of a methodology to access heteroleptic imidophosphorane complexes of U^4+^ and the synthesis and characterization of U^4+^ alkyl complexes supported by imidophosphorane complexes. The oxidation chemistry is evaluated electrochemically and chemically, and a putative U^5+^ alkyl species is found to undergo reductive U–C bond homolysis en route to a cationic, heteroleptic U^4+^ complex. Additionally, low-temperature hydrogenolysis conditions enable a terminal hydride complex to be spectroscopically and crystallographically identified. These synthetic studies define the challenges in extending these methodologies to Np and Pu.

## Results

### Synthesis

A uranium monoiodide complex was synthesized by salt metathesis in the reaction between [UI_4_(1,4-dioxane)_2_] and 3 equiv of K­[N = P­(*N*,*N*′-di*tert*-butylethylenediamide)­(diethylamide)]­(NP*) in hexanes for 16 h. Purple, needle-like single-crystal, X-ray diffraction (SC-XRD) quality crystals of **1** were obtained from hexanes at −35 °C. The resulting crystals were recrystallized in hexanes twice to remove byproducts (principally HNP* which is not volatile and soluble in hexanes), for bulk isolation of **1** in 62% yield overall. Alkylation of **1** with 1.1 equiv of benzyl potassium, or 1.1 equiv of neopentyl lithium, or 1.6 equiv of methyl potassium yields the benzyl complex, [U^4+^(NP*)_3_CH_2_(C_6_H_5_)] (**2**), the neopentyl complex, [U^4+^(NP­(NP*)_3_CH_2_C­(CH_3_)] (**3**), and [U^4+^(NP*)_3_CH_3_] (**4**) in 42%, 77%, and 73% yields, respectively (Scheme 1). Complexes **2** and **3** are relatively stable under the reaction conditions, and the reaction mixtures were allowed to stir in diethyl ether (Et_2_O) for 16 h at room temperature, whereas modifications were necessary in the synthesis of **4** to account for the low solubility of methyl potassium. At −40 °C, 1.6 equiv of methyl potassium was added to **1**, where the reaction mixture was warmed up gradually to room temperature and stirred for 3 days, and **4** was collected as pink crystals from pentane in 73% yield.

**Scheme 1:**
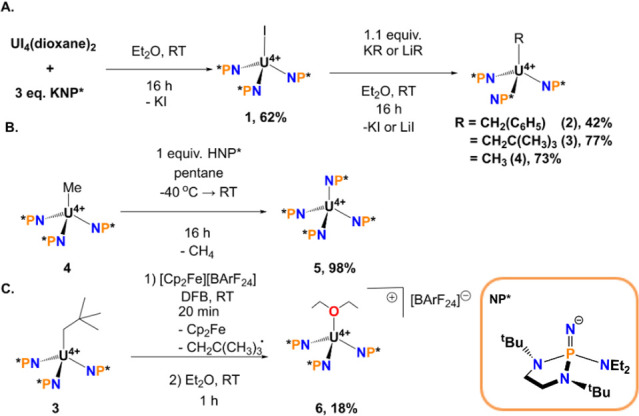
(A) Synthetic Scheme of U^4+^ Monoiodide (**1**), Benzyl (**2**), Neopentyl (**3**), and Methyl (**4**) Complex. (B) Protonolysis of **4** to Synthesize the U^4+^ Tetrahomoleptic Complex, **5**. (C) Synthesis of the Cationic, Et_2_O Coordinated Complex, **6**. Depiction of the NP* Ligand is Shown in the Orange Inset

In contrast to the monoiodide and alkyl complexes, the tetrahomoleptic NP* complex cannot be accessed through direct salt metathesis between K­[NP*] and [UI_4_(1,4-dioxane)_2_]. Even with 4 equiv of K­[NP*] added to [UI_4_(1,4-dioxane)_2_], the reaction stopped at **1**. This result is distinct from the +3 lanthanide analogues, where the anionic, tetrahomoleptic complexes K­[Ln­(NP*)_4_] can be synthesized for Ce, Pr, Nb, Tb, Dy, and Gd through salt metathesis, and the Ce, Pr, and Tb complexes can be oxidized to access the +4 complexes, [Ln­(NP*)_4_] (Ln = Ce, Pr, Tb).^22,32,34^ Furthermore, the [NP­(^*t*^Bu)_3_]^1–^ ligand, which forms a monoiodide complex, [Ce^4+^(I)­(NP­(^*t*^Bu)_3_)_3_], upon addition of substoichiometric amount of K­[NP­(^*t*^Bu)_3_] and subsequent oxidation, instead forms a tetrahomoleptic complex, [U^4+^(NP^t^Bu_3_)_4_], upon addition of 4 equiv of K­[NP­(^*t*^Bu)_3_].^26^ This dichotomy highlights how, despite the similar ionic size between Ce^4+^(0.87 Å) and U^4+^(0.89 Å), electronic and steric consideration, along with redox chemistry, are essential to prepare heteroleptic imidophosphorane complexes.^35^

The homoleptic uranium complex [U^4+^(NP*)_4_] (**5**) can be instead synthesized through protonolysis of the methyl complex, **4**, with 1 equiv of HNP* in pentane at −40 °C and isolated as pink crystals from pentane at −35 °C in 98% yield (Scheme 1). Addition of 1 equiv of Fc­(BArF_24_) to the neopentyl complex, **3**, in 1,2-difluorobenzene followed by crystallization from Et_2_O leads to a cationic tetravalent uranium complex, [U^4+^(NP*)_3_(Et_2_O)]­[BArF_24_] **(6**), as brown crystals (18% yield).

### Crystallographic Analyses

Molecular structures of complexes **1**–**6** were characterized by SC-XRD (Figure 1 and Table 1). **1** and **4** crystallize in the *P*2_1_/*c* space group with one formula in the asymmetric unit, with **4** showing disorder on the NP* ligand. **2** crystallizes in **P*1̅*, and within the asymmetric unit, one formula unit is found with **1** cocrystallizing at 0.0367 occupancy. **3** crystallizes in the *Pbca* space group, with two molecules in the asymmetric unit, with one formula demonstrating disorder at the neopentyl ligand and the NEt_2_ fragment on each NP* ligand. **5** crystallizes in the 4-fold *I*4̅ space group, as a two-component inversion twin, whereas **6** crystallizes in the *P*1̅ space group.

**Figure 1:**
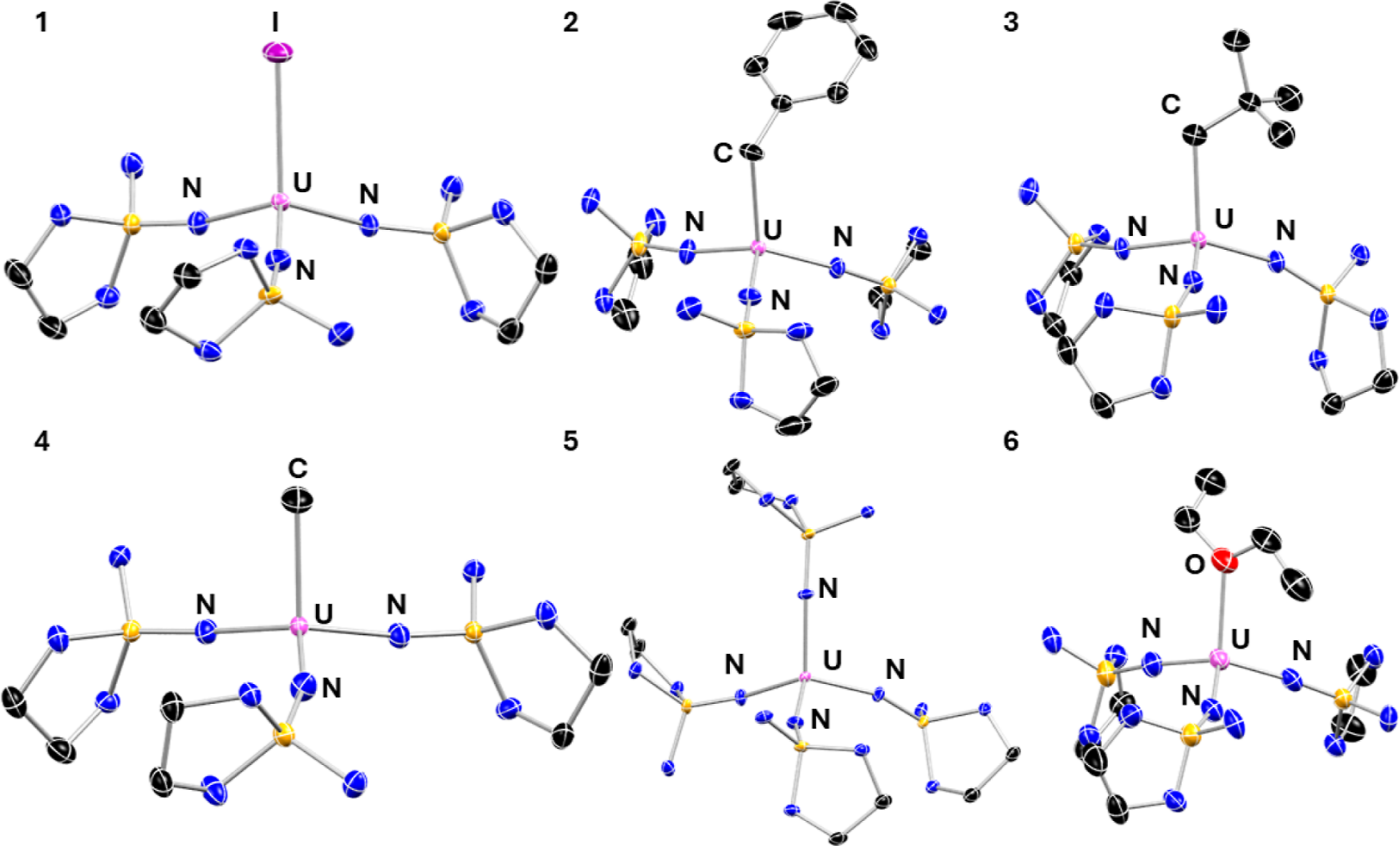
Molecular structures of complexes **1**–**6** determined from SC-XRD with thermal ellipsoids shown at 50% probability. (Pink = uranium, blue = nitrogen, orange = phosphorus, black = carbon, red = oxygen, purple = iodine). H atoms, ^*t*^Bu group, and Et group on the NP* ligand and [B­(ArF_6_)]_4_ anion in **6** are omitted for clarity.

**Table 1:** Structure Metrics and Calculated τ_4_ Values^36^ from SC-XRD for **1**–**6** ^a^

	*d*(U–N)_avg_ (Å)	D(U–E) (Å)	τ_4_
**1**	2.137 (3)	3.0610 (3)	0.97
**2**	2.152 (8)	2.532 (4)	0.92
**3** ^b^	2.174 (11)	2.506 (5)	0.93
	2.175 (5)	2.494 (11)	0.92
**4**	2.168 (8)	2.448 (2)	0.89
**5** ^b^	2.2012 (19)	N/A	0.95
	2.2013 (19)	N/A	0.95
**6**	2.12 (2)	2.530 (9)	0.93

a E = I for **1**, C for **2**–**4**, and O for **6**.

b Two values are shown for **3** and **5** as these structures contain two molecules in the asymmetric unit.

All of the complexes, **1**–**6**, are pseudotetrahedral complexes within the first coordination sphere, albeit with small deviations in the geometry index, τ_4_ (Table 1).^36,37^ The iodide complex, **1**, shows a near tetrahedral symmetry with a τ_4_ = 0.97. This value is on par with the tetrahomoleptic imidophosphorane complex, [U^4+^(NP­(^*t*^Bu)_3_)_4_], which has a τ_4_ = 0.98.^26^ In particular, the iodide complex, **1**, and the methyl complex, **4**, share the same space group *P*2_1_/*c*, yet the τ_4_ values between the two are substantially different. The alkyl complexes deviate slightly from ideal tetrahedral geometry, with τ_4_ = 0.92 (**2**), 0.93 (**3**), and 0.89 (**4**). τ_4_ of **5** is at 0.95, close to an ideal tetrahedral geometry.

Averaged U–N distances (U–N_avg_) of the heteroleptic complexes **1**–**4** and **6** fall within the range 2.12(2) (**6**)–2.158­(8) Å (**4**), shorter than the U–N_avg_ at 2.201 (3) Å of the tetrahomoleptic complex, **5**, suggestive of less steric repulsion for the heteroleptic complexes (Table 1). Compared to the previously reported tetrahomoleptic U^4+^ complexes supported by imidophosphorane ligands, these U–N_avg_ distances are shorter than U–N_avg_ = 2.19 (5) Å of [U­(NP­(pip)_3_)_4_, (pip = piperidinyl)] and U–N_avg_ = 2.185 (2) Å of [U­(NP­(^*t*^Bu)_3_)_4_,] and comparable to U–N_avg_ of 2.167 (7) Å of [U­(NP^t^Bu­(pyrr)_2_)_4_] (pyrr = pyrrolidinal).^25–27^ Compared to the Ce^4+^-benzyl complex supported by the imidophosphorane ligand, [NP^*t*^Bu_3_]^1–^, [Ce^4+^(η^2^-CH_2_Ph)­(NP­(^*t*^Bu)_3_)] where the benzyl ligand has a η^2^ coordination, **2** can be considered as a η^1^ coordination based on the U–CH_2_–C_ipso_ angle of 121.3(3)°.^31^ Given the change in the imidophosphorane ligand and small change in metal ionic radii, it is unclear whether this coordination geometry change is steric or electronic in origin.

The tetrahomoleptic complex, **5**, is distinct from the heteroleptic monoiodide and the alkyl complexes. The *I*4̅ space group is shared among the previously crystallographically characterized [Ln^4+^(NP*)_4_] complexes, [Ce^4+^(NP*)_4_][Bibr ref32] and [Tb^4+^(NP*)_4_].^22^ U^4+^ tetrahomoleptic complexes of smaller imidophosphorane ligands do not possess the same 4-fold symmetry. [U­(NP­(pip)_3_)_4_][Bibr ref25] crystallizes in the *R*3 space group, [U­(NP^*t*^Bu­(pyrr)_2_)_4_][Bibr ref27] and [U­(NP­(^*t*^Bu)_3_)_4_][Bibr ref26] both crystallize in the *P*2_1_/*n* space group.

The U–N_avg_ distance of 2.12(2) Å of complex **6** (Table 1) is shorter than the neutral, alkyl complexes **1** – **4**. Shortening of the U-L distance in the cationic complex by 0.03–0.06 Å has also been reported in the cationic U^4+^ alkyl complex, [(XA_2_)­U­(CH_2_SiMe_3_)­(η^6^-C_6_H_6_)]­[B­(C_6_F_5_)_4_]·2­(C_6_H_6_) [{XA_2_ = 4,5-bis­(2,6-diisopropylanilido)-2,7-di*tert*-butyl-9,9dimethylxanthene}] compared to its neutral analog, [(XA_2_)­U­(CH_2_SiMe_3_)_2_].^38^ The U^4+^–OEt_2_ distance in **6** is 2.530(9) Å, which is slightly longer than the U^4+^–OEt_2_ distance in an alkyl cation Et_2_O coordinated complex, [Fc­(NSi^*t*^BuMe_2_)_2_U­(CH_2_Ph)­(OEt_2_)]­[BPh_4_] at 2.4112(63) Å.^39^

### Nuclear Magnetic Resonance (NMR) Spectroscopy

The solution NMR reveals solution structures congruent with the solid-state assignments by SC-XRD. ^31^P­{^1^H}­NMR shows a single resonance at 456.61 ppm (**1**), 443.50 ppm (**2**), 439.52 ppm (**3**), 454.31 ppm (**4**), 357.45 ppm (**5**), and 373.07 ppm (**6**) which are consistent with tetravalent uranium imidophosphorane 5f^2^ complexes previously reported.^25–27^ In the alkyl complexes, methylene­(−CH_2_) protons bonded to the U^4+^ metal center are shifted upfield at chemical shifts of −172.55 ppm (**2**) and −160.35 ppm (**3**), while the methyl (−CH_3_) protons are less shifted at −1.28 ppm (**4**). The substantial upfield shift in the alpha–CH_2_ complex is rationalized by the direct interaction of the protons with the paramagnetic f^2^ center of U^4+^ ion.^38,40^ Consequently, the ^13^C chemical shifts of the methylene carbons cannot be located, consistent with previous reports on U^4+^ alkyl complexes.^2^ One molecule of Et_2_O at 1.10 and 3.29 ppm is observed per molecule of **6** (Supporting Information Figure S24). These resonances are nearly identical to free diethyl ether, in contrast to the previously reported cationic U^4+^ Et_2_O complex, [Fc­(NSi^*t*^BuMe_2_)_2_U­(CH_2_Ph)­(OEt_2_)]­[BPh_4_] where stoichiometric Et_2_O resonances were identified at 1.73 ppm (OCH_2_CH_3_) and −10.30 ppm (OCH_2_CH_3_).^39^ These results suggests that the diethyl ether is relatively weakly bound and in equilibrium with uncoordinated diethyl ether under NMR conditions.

### Electronic Absorption Spectroscopy

UV–vis–NIR (ultraviolet–visible–near-infrared) absorption spectroscopy of **1**, **2**, **3**, **4**, and **5** (Figure 2) all demonstrate Laporte forbidden *f*–*f* transitions from 468 to 1816 nm with molar extinction coefficients over the range of 5 M^–1^cm^–1^ to 67 M^–1^cm^–1^ which are characteristic of tetravalent uranium complexes.^25,41–43^ Furthermore, charge transfer features are observed at 320 nm (**1**), 355 nm (**2**), 352 nm (**3**), 349 nm (**4**), 312 nm (**5**) (Supporting Information Figure S33). These LMCT features are consistent with previous reports of imidophosphorane LMCT energies in U^4+^ complexes.^25,43^ Notably, the heteroleptic complexes **1**, **2**, **3**, and **4**, where the only differences are the iodide/alkyl ligands, mostly share the same transitions throughout the UV–vis region. Nonetheless, in the NIR region, the transition between 1400 and 1600 nm is sensitive to the change in the heteroligand identity. The tetrahomoleptic complex, **5**, is unique from these heteroleptics. In addition to a red-shifted peak at 1545 nm (ε = 48 M^–1^cm^–1^), the feature that is most sensitive to the identity of the heteroleptic ligand in complexes **1**–**4**, it also has well-resolved peaks at 964 nm (ε = 26 M^–1^cm^–1^) and 1259 nm (ε = 49 M^–1^cm^–1^). The resolution of these features is common among other homoleptic uranium imidophosphorane complexes.

**Figure 2:**
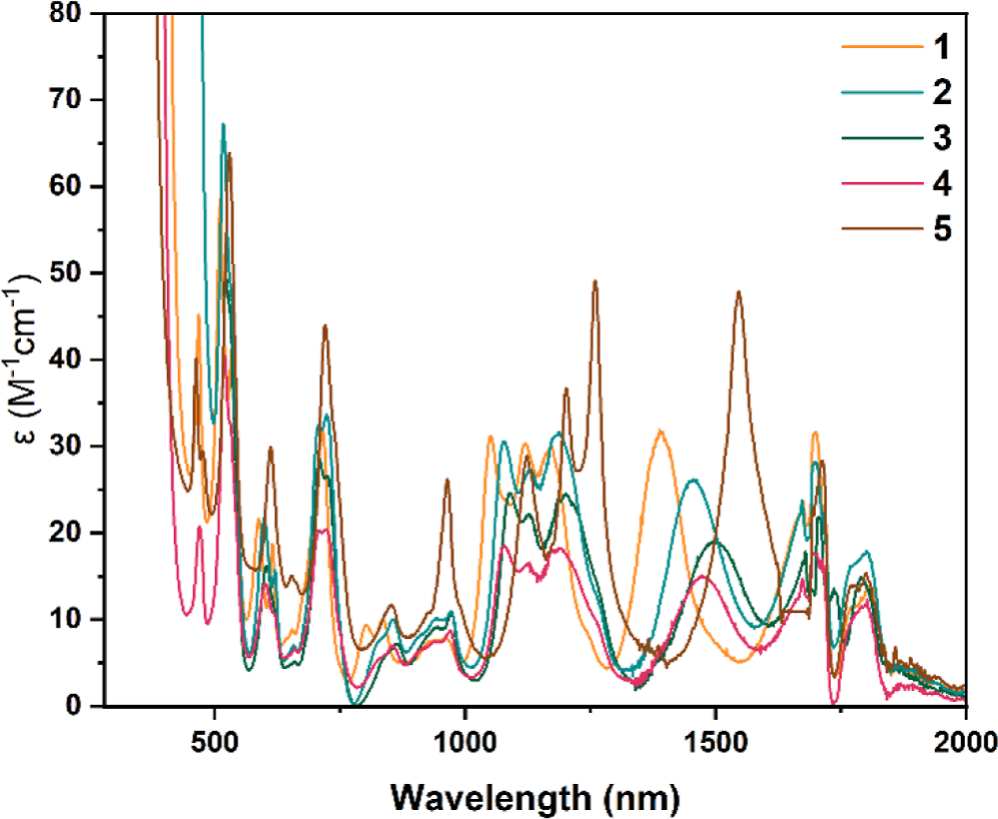
Molar absorptivity of 1 (8.7 mM), 2 (9.1 mM), 3 (8.9 mM), 4 (8.3 mM), 5 (6.3 mM) in THF to show *f*–*f* transitions.

### Electrochemical and Chemical Reactivity of **1** and **3**

Redox profiles of **1** and **3** were studied employing cyclic voltammetry, with 3 mM analyte in 0.1 M [^*n*^Bu_4_N]­[PF_6_] in 1,2-difluorobenzene (Figure 3) (cyclic voltammograms of **1** and **3** at 3 mM analyte concentration in 0.1 M [^n^Bu_4_N]­[PF_6_] in 1,2-difluorobenzene at the scan rate of 200 mV/s). Initial potential and the scan direction are indicated by the arrow. It was observed that **2** deposits onto the electrode, therefore, a satisfactory CV of **2** could not be obtained. CV of both **1** and **3** display electrochemical reduction at *E*_pc1_ = −1.03 V and −1.26 V, respectively, vs Fc^+^/Fc. Oxidation of **1** and **3** was observed at *E*_pa1_ = −0.84 V and −1.06 V vs Fc^+^/Fc. This redox couple is assigned as a reversible U^5+/4+^ couple, anodically shifted compared to −1.62 V of U^4+^[NP­(^*t*^Bu)_3_]_4_ and −1.57 V of [U^4+^(NP^*t*^Bu­(pyrr)_2_)_4_].^26,28^ The U^4+/3+^ redox couple was not observed within the solvent window for either complex. **1** shows another couple at *E*_pc2_ = −0.61 V and *E*_pa2_ = −0.42 V. The more negative *E*_pc1_ value of **3** compared to **1** follows the trend observed in tetravalent cerium alkyl and iodide complexes, emphasizing the increased electrochemical stability of the neopentyl complex compared to the iodide complex.^31^ There is a secondary feature observed in **1** at *E*_1/2_ = −0.51 V, which is also observed in **3** around *E*_1/2_ = −0.75 V yet poorly resolved. This feature is considered to originate from solution behavior or speciation, consistent with the Ce^4+^ monoiodide and alkyl complexes.^31^ A third oxidation peak is observed for **1** at 0.33 V, without the corresponding reduction peak, which could be either the oxidation of the iodine ligand or an irreversible U^6+/5+^ peak. In **3**, the secondary couple is not as clearly resolved as compared to **1**. However, consistent with **1**, the oxidation peak at *E*_pa2_ = 0.80 V could be assigned as the oxidation of neopentyl ligand or an irreversible metal-based oxidation.

**Figure 3:**
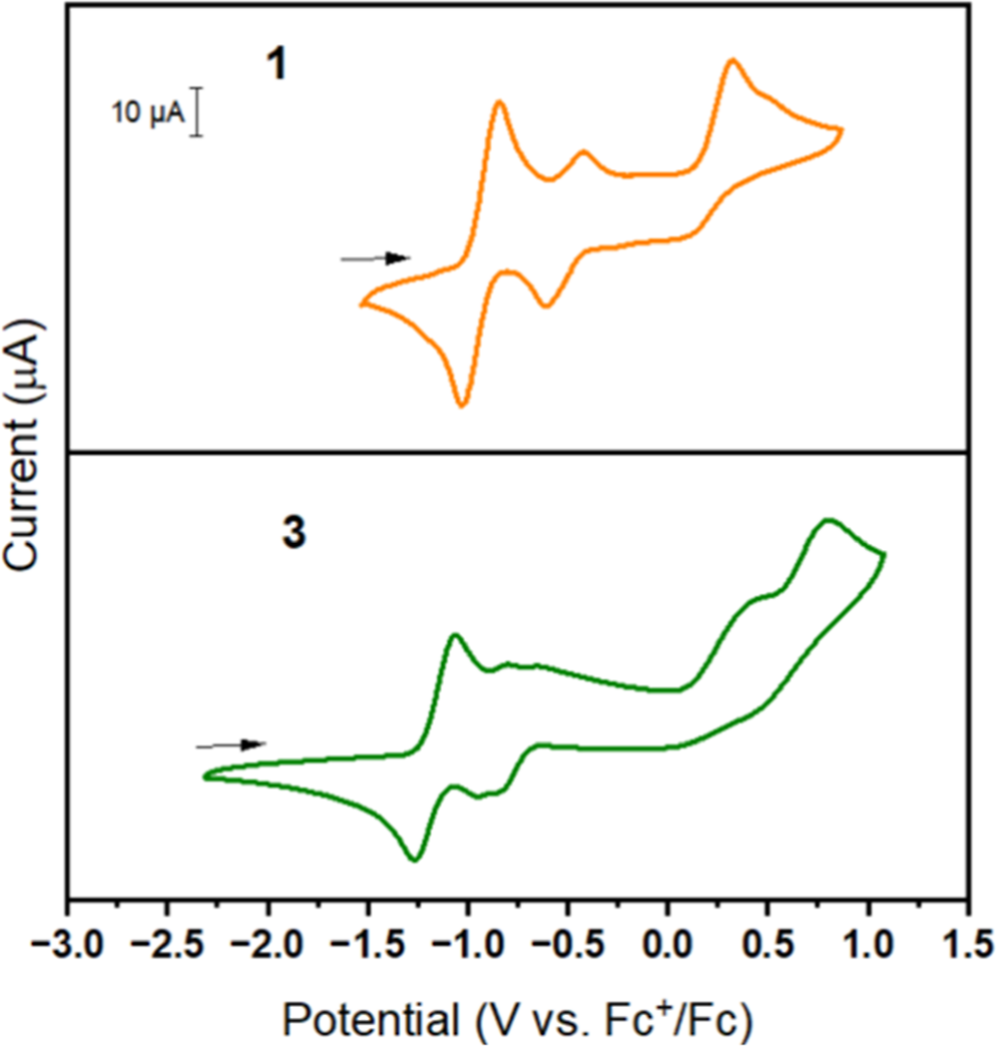
Cyclic voltammogram of 1 and 3 at 3 mM analyte concentration in 0.1 M [^*n*^Bu_4_N]­[PF_6_] in 1,2-difluorobenzene at the scan rate of 200 mV/s. Initial potential and the scan direction are indicated by the arrow.

In light of the reversible U^5+/4+^ couple observed in the cyclic voltammogram of **3** at *E*_1/2_ = −1.16 V, chemical oxidation of **3** was performed. Silver salts such as AgCl, AgI, and AgBArF_20_ did not result in a color change with **3**, which warranted the use of ferrocenium oxidants. Reaction of **3** with 1 equiv of FcBArF_24_ in PhF produces an unisolated intermediate with a ^31^P­{^1^H} NMR chemical shift of 100.68 ppm (Supporting Information Figure S23), a shift from 439.52 ppm of **3**, consistent with ^31^P­{^1^H} shift of previously reported U^5+^ complex, [U^5+^(NP^t^Bu­(pyrr)_2_)_4_]­[BArF_20_], at 94.48 ppm and that of [U^5+^(NP^t^Bu_3_)_4_] at 84.65 ppm.^26,28^ Attempts to isolate this potential U^5+^ alkyl complex were frustrated by chemical incompatibility with sufficiently high-dielectric solvents necessary to solubilize the salt. Use of even the mildly coordinating solvent, Et_2_O, a solvent coordinated, cationic tetravalent complex **6** is obtained (Scheme 1). Cationic, ethereal complexes of U^4+^ have limited precedent.^39^ A similar reactivity was observed by Monreal and Diaconescu in the isolation of [Fc­(NSi^*t*^BuMe_2_)_2_U­(CH_2_Ph)­(OEt_2_)]­[BPh_4_].^39^

### Spectroscopic Identification of a Terminal Hydride Intermediate

The potential hydride formation via hydrogenolysis of **3** with H_2_ gas was considered. The reaction of **3** with H_2_ gas at low temperature (<−20 °C) yields a mixture of the starting material **3** and a terminal hydride intermediate (**7-H**) that could be observed in ^1^H and ^31^P­{^1^H} NMR spectra at room temperature (Scheme 2 and Figure 4) via in situ analysis of reactions in J. Young NMR tubes. ^31^P­{^1^H} NMR spectra show a new peak appearing at 455.90 ppm (Supporting Information Figure S28). Furthermore, a singlet peak that integrates to one with respect to the ligand *tert*-butyl resonances in the ^1^H spectrum is located substantially downfield at 250.43 ppm, assigned to the U–H resonance which is indicative of bonding to the paramagnetic, tetravalent uranium with f^2^ electronic configuration. In order to confirm that this chemical shift responds to a hydride, the reaction of **3** with D_2_ gas was performed, wherein the U–D resonance is observed at 244.70 ppm in ^2^H (D) NMR.

**Scheme 2:**
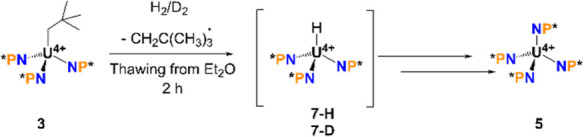
Reaction of **3** with H_2_/D_2_ to form Spectroscopically and Crystallographically Identifiable Uranium Hydride Intermediates (**7-H** and **7-D**), which Disproportionate to **5**

**Figure 4:**
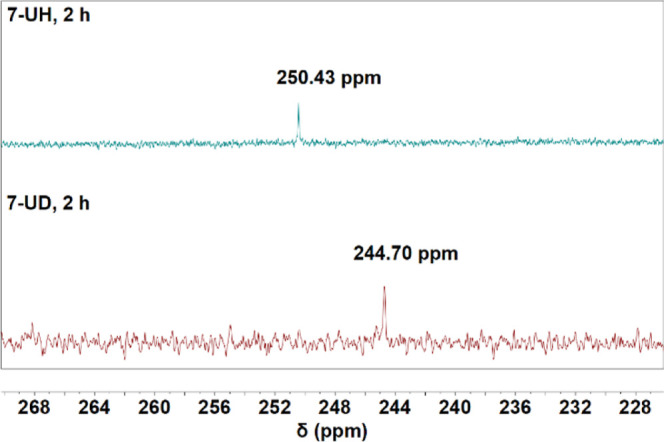
^1^H NMR of 7-H (400 MHz, C_6_D_6_) and ^2^H NMR of 7-D (61 MHz, C_6_D_6_) overlaid showing the U–H and U–D resonance in the 270–226 ppm region.

The hydride intermediate, **7-H**, eventually degrades to the tetrahomoleptic complex (**5**) as the temperature rises to room temperature as confirmed by ^1^H NMR and ^31^P­{^1^H} NMR (Supporting Information Figures S27, S28 and Scheme 2). **5** can be prepared independently (via protonolysis of **4** (Scheme 1) as discussed above) facilitating its spectroscopic assignment in these reaction mixtures. When handled at cold temperature under H_2_ (<−20 °C), SC-XRD quality yellow crystals of **7-H** could be crystallized from the reaction mixture by redissolving in pentane and cooling at −35 °C for 16 h. **7-H** crystallizes in the *P*2_1_/*n* space group with 3 NP* ligands (Figure 5). The metal bound hydride could not be located in the difference map due to the large electron density difference between the proton and the uranium atom. The found U–N_avg_ = 2.151 (11) Å is consistent with the U–N_avg_ of **1**–**5**. Clean, bulk isolation of **7-H** could not be achieved due to the thermal instability of **7-H**. The NMR and IR indicate the presence of the starting material, **7-H**, and **5** in attempts for a bulk synthetic procedure. Conditions to selectively form **7-H** and enable bulk characterization could not be identified.

**Figure 5:**
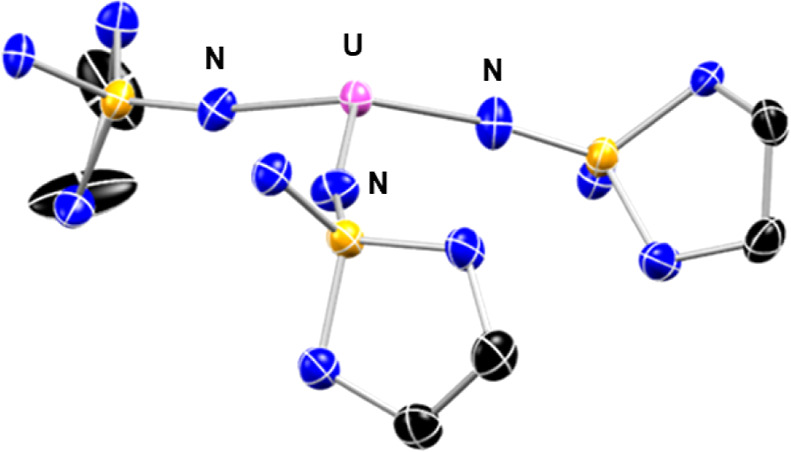
Molecular structure of **7-H** determined from SC-XRD with thermal ellipsoids shown at 50% probability (pink = uranium, blue = nitrogen, orange = phosphorus). H atoms, ^*t*^Bu group, and Et group on the ligand are omitted. U–H proton is not located from the SC-XRD.

A few terminal, mononuclear U^4+^ hydride complexes are known either crystallographically and/or as bulk characterized materials including complexes by Ephritikhine and co-workers [U^4+^(C_5_H_4_SiMe_3_)_3_H] and [U^4+^(C_5_H_4_^*t*^Bu)_3_H][Bibr ref16] and Walensky and co-workers recent report of [U^4+^(C_5_Me_5_)_2_(2,6-^*t*^Bu)_2_-4-MeC_6_H_2_O)­(H)] ^18,19^ and [U^4+^(C_5_Me_5_)_2_(2,4,6-Me_3_C_6_H_2_O)­(H)].^21^ These complexes (and one observed in situ [U^4+^(C_5_Me_5_)_2_(MesO)­(H)]) have ^1^H NMR shifts that agree well with that observed for **7-H** of 250.43 ppm for the U–H resonance, such as U–H resonance of 290.5 ppm for [U^4+^(C_5_H_4_SiMe_3_)_3_H] and 276.1 ppm for [U^4+^(C_5_H_4_^*t*^Bu)_3_H].^16^ The U–H resonances of 272.8 ppm for [U^4+^(C_5_Me_5_)_2_(MesO)­(H)], 352.3 ppm for [U^4+^(C_5_Me_5_)_2_(2,6-^*t*^Bu)_2_-4-MeC_6_H_2_O)­(H)],^18,19^ and 283.8 ppm for [U^4+^(C_5_Me_5_)_2_(2,4,6-Me_3_C_6_H_2_O)­(H)][Bibr ref21] further corroborate the formation of **7-H**, as represented in the molecular structure (Figure 5). Terminal uranium hydride complexes are often thermally unstable. However, the thermal reactivity of **7-H** is noteworthy. The formation of **5** under warming in an H_2_ atmosphere indicates a ligand disproportionation reaction and the formation of free phosphinimine (HNP*) via U–N bond hydrogenolysis. Activation of nitrogen and hydrogenation with U^3+^ and U^4+^ hydrides have been recently reported.^44,45^ The potential for direct activation of H_2_ by metal–imidophosporane complexes may be useful for reaction development.

## Conclusion

U^4+^ alkyl and benzyl complexes (**2**, **3**, **4**) were synthesized through salt metathesis of the U^4+^ monoiodide complex, **1**. Spectroscopic characterization of these complexes through NMR, UV–vis–NIR, and SC-XRD confirms the +4 oxidation state, with similar electronic structures as observed from UV–vis–NIR spectra. The U^4+^ methyl complex, **4**, affords the tetrahomoleptic complex, **5**, upon protonolysis, which is otherwise synthetically inaccessible through salt metathesis. Leveraging the robust platform provided by the NP* ligand, further reactivity studies of **3** revealed that oxidation does not yield an isolable U^5+^ oxidation product as indicated by cyclic voltammetry. Rather, upon exposure to a coordinating solvent, Et_2_O, a cationic U^4+^ ether complex, **6**, is isolated from the formal homolytic cleavage of the U–C bond. Furthermore, upon hydrogenolysis of **3** with H_2_ gas, a terminal uranium hydride complex (**7-H**) was characterized in situ through NMR and SC-XRD. The identity of the terminal hydride was further supported by formation of the deuteride via reaction of **3** and D_2_ as indicated by in situ ^2^H NMR. **7-H** is thermally unstable under reaction conditions (excess H_2_) and decomposes to the tetrahomoleptic complex, **5**. This study demonstrates the utility and limitations of the NP* framework to support alkyl and hydride ligands in the mid-actinides and maps the synthetic constraints in translating this chemistry to neptunium and plutonium.

## Experimental Section

### General Considerations (Synthesis)

*Caution!* Depleted uranium which mainly contains ^238^U is a weak α-emitter (4.197 MeV, *t*_1/2_ = 4.47 × 109 years). Handling of depleted uranium requires a well-ventilated fume hood or a glovebox and appropriate radiological control measures are necessary.

Otherwise noted, all manipulations were conducted in a N_2_-filled glovebox (Vigor, <0.1 ppm of O_2_/H_2_O) or Schlenk line (UHP Ar). Et_2_O, pentane, hexanes, and THF were purged through UHP Ar and dried through Q-5/alumina and molecular sieves in solvent purification system (JC Meyer Systems, Pure Process Technology). HMDSO and 1,2-difluorobenzene were dried over 3 Å molecular sieves for 48 h, then subsequently distilled and stored over dry 3 Å molecular sieves for >48 h prior to use. H_2_ and D_2_ gases (UHP, Matheson) were purchased in 55 L lecture bottles and used as received. Starting materials UI_4_(1,4-dioxane)_2_, KNP*, HNP*, and KBn were prepared following previously reported procedures.^4,22,46^ Neopentyllithium was modified from the previously reported procedure, substituting neopentyl bromide with neopentyl chloride.^47^ KMe was prepared by following a previous report.^48^ [Fc­(BArF_24_)] was synthesized following a procedure for [Fc­(BArF_20_)].^30^

### [U^4+^I­(NP­(1,2-bis-^t^Bu-diamidoethane)­(NEt_2_))_3_], **1**

UI_4_(1,4-dioxane)_2_ (719 mg, 0.78 mmol) was dissolved in 10 mL of Et_2_O in a 50 mL pear Schlenk flask equipped with a PTFE stir bar. In a 20 mL vial, [(CH_2_N^t^Bu)_2_(Et_2_N)P = NK] (764 mg, 2.34 mmol) was dissolved in 10 mL of Et_2_O and added as a solution to the reaction vessel, slowly. The reaction mixture turned dark and within 10 min formed a purple slurry. This reaction mixture was allowed to stir for 16 h, at which point a purple precipitate formed with a brown solution. The resulting slurry was filtered through a 15 mL fine frit packed with Celite. The brown filtrate was dried in vacuo, yielding a purple-brown solid, followed by trituration with pentane (1 mL × 3). Then, the purple-brown solid was extracted with 10 mL of hexanes in a 20 mL vial, which was then filtered through Celite pipet filter. The brown solution was then concentrated to 5 mL, at which point purple crystals started to form on the sides of the vial. The purple crystals were dissolved back into solution, and the concentrated hexanes solution was placed in the freezer at −35 °C, yielding purple brown crystals overnight. The resulting purple brown crystals were recrystallized from hexanes again at −35 °C to yield purple crystals, which was then decanted and dried in vacuo to a purple powder (592.1 mg, 62%). SC-XRD quality crystals were obtained from hexanes. ^1^H NMR (500 MHz, C_6_D_6_): δ 21.55 (broad s, 12H, N**CH**_**2**_CH_3_), 10.12 (s, 6H, N**CH**_**2**_CH_2_N), 8.84 (s, 6H, NCH_2_ **CH**_**2**_N), 7.45 (s, 18H, NCH_2_ **CH**_**3**_), −0.25 (s, 54H, –C­(**CH**_**3**_)_3_). ^13^C­{^1^H} NMR (126 MHz, C_6_D_6_): δ 57.94, 48.39, 34.98, 20.92, 12.92. ^31^P­{^1^H} NMR (203 MHz, C_6_D_6_): δ 454.45. IR (cm^–1^): 2966 (m), 2925 (m), 2853 (m), 1459 (w), 1390 (m), 1377 (m), 1359 (m), 1247 (m), 1203 (m), 1147 (m), 1098 (s), 1049 (s), 1019 (s), 977 (m), 937 (m), 867 (m), 799 (m), 707 (m), 644 (m), 593 (w), 516 (m), 499 (m), 450 (m). Elemental Analysis % found (calculated) for C_42_H_96_IN_12_P_3_U: C, 39.52 (41.11); H, 7.94 (7.84); N, 13.20 (13.70). Carbon was consistently low on duplicate analyses.

### [U^4+^(NP­(1,2-bis-^t^Bu-diamidoethane)­(NEt_2_))_3_CH_2_(C_6_H_5_)], **2**

**1** (288 mg, 0.23 mmol) was dissolved in 5 mL of hexanes in a 20 mL scintillation vial equipped with a glass stir bar. In a 4 mL vial, benzylpotassium­(33 mg, 0.26 mmol) was weighed out. Benzyl potassium was added as a solid to the solution of **1-UI** with stirring, followed by a wash of the 4 mL vial using 3 mL of hexanes. Reaction mixture turned from purple to orange-yellow over 16 h of stirring at RT. After 16 h of stirring, benzylpotassium­(17 mg, 0.13 mmol) was added to ensure the reaction goes to completion. The reaction mixture was stirred for another 16 h. The resulting orange-yellow slurry was filtered through a glass pipet filled with glass fiber filter and Celite to yield a yellow solution. The solution was dried in vacuo and triturated with pentane (1 mL × 3), yielding a yellow, bubbly solid. The crude material was extracted with 6 mL of pentane, filtered through pipet packed with glass fiber and Celite, and concentrated to ≈2 mL in vacuo and cooled at −35 °C for 16 h to yield yellow crystals (108 mg). Supernatant solution was further concentrated in vacuo and resubjected to cooling at −35 °C for 16 h to yield a second crop of yellow crystals (11 mg). Crystals were dried in vacuo to yield a yellow-brown powder (combined mass 118 mg, 42.4% total yield). ^1^H NMR (500 MHz, C_6_D_6_): δ 18.30 (broad s, 12H, NCH_2_ **CH**_**3**_), 9.59 (s, 6H, N**CH**_**2**_CH_2_N), 7.88 (s, 6H, NCH_2_ **CH**_**2**_N), 5.97 (s, 18H, NCH_2_ **CH**_**3**_), 0.46 (s, 54H, –C­(**CH**_**3**_)_3_), −1.51 to −3.96 (m, 4H, Ph–CH), −29.88 (s, 1H, Ph–C**H**), −172.55 (s, 2H, U–**CH**_**2**_-Ph). ^13^C­{^1^H} NMR (126 MHz, C_6_D_6_): δ 113.73, 94.77, 58.67, 47.85, 19.93, 12.32. ^31^P­{^1^H} NMR (203 MHz, C_6_D_6_): δ 441.83. IR (cm^–1^): 2966 (m), 2928 (m), 2846 (m), 1589 (m), 1460 (w), 1387 (w), 1358 m), 1249 m), 1193 (s), 1145 (m), 1101 (s), 1051 (m), 1021 (s), 978 (m), 933 (m), 910 (m), 860 (m), 790 (w), 705 (s), 499 (s). Elemental analysis % found (calculated) for C_49_H_103_N_12_P_3_U: C, 49.56(49.40); H, 8.96(8.71); N, 13.85(14.11).

### [U^4+^(NP­(1,2-bis-^t^Bu-diamidoethane)­(NEt_2_))_3_CH_2_C­(CH_3_)], **3**

**1** (267 mg, 0.22 mmol) was dissolved in 3 mL of Et_2_O in a 20 mL scintillation vial equipped with a glass stir bar. In a 4 mL vial, neopentyllithium (17 mg, 0.22 mmol) was dissolved in 1 mL of Et_2_O. Solution of neopentyllithium was added to the solution of **1-UI** with stirring. Upon addition of neopentyllithium, an immediate color change from purple to yellow was observed. The reaction mixture was stirred at RT for 2 days, at which point a yellow solution with a small amount of colorless precipitate formed. The resulting suspension was filtered through a pipet filter packed with glass fiber filter and Celite to yield a yellow, clear solution. The yellow solution was dried in vacuo and triturated with pentane (1 mL × 3) to yield a yellow, bubbly solid. Subsequently, the yellow solid was extracted with 5 mL of Hexamethyldisiloxane (HMDSO), then filtered through pipet filter packed with glass fiber and Celite, and concentrated to ≈1.5 mL in vacuo and cooled at −35 °C. Yellow SC-XRD quality crystals (71 mg) formed over 16 h, and the supernatant solution was further concentrated and cooled at −35 °C for another 16 h to yield a second crop of yellow crystals (125 mg). Crystals were dried in vacuo to yield a yellow powder (combined mass 196 mg, 77% total yield). ^1^H NMR (500 MHz, C_6_D_6_): δ 16.67 (broad s, 12H, NCH_2_ **CH**_**3**_), 9.16 (s, 6H, N**CH**_**2**_CH_2_N), 7.38 (s, 6H, NCH_2_ **CH**_**2**_N), 5.04 (s, 18H, NCH_2_ **CH**_**3**_), 1.03 (s, 54H, –C­(**CH**_**3**_)_3_), −26.93 (s, 9H, U–CH_2_–C­(**CH**_**3**_)_**3**_), −160.35 (s, 2H, U–**CH**_**2**_–C­(CH_3_)_3_). ^13^C­{^1^H} NMR (126 MHz, C_6_D_6_): δ 58.59, 47.36, 18.96, 12.85. ^31^P­{^1^H} NMR (203 MHz, C_6_D_6_): δ 438.41. IR (cm^–1^): *v* 2967 (m), 2926 (m), 2851 (m), 1460 (s), 1388 (s), 1377 (m), 1357 (m), 1266 (m), 1248 (m), 1194 (m), 1105 (w), 1052 (w), 1021 (w), 976 (m), 934 (m), 867 (m), 798 (m), 704 (m), 640 (m), 593 (m), 516 (m), 499 (w), 471 (m), 448 (m). Elemental analysis % found (calculated) for C_47_H_107_N_12_P_3_U: C, 47.95(48.19); H, 9.11(9.21); N, 14.18(14.35).

### [U^4+^(NP­(1,2-bis-^t^Bu-diamidoethane)­(NEt_2_))_3_CH_3_], 4

A cold (−40 °C) suspension of methyl potassium (25 mg, 0.47 mmol) in 5 mL of hexanes was charged with a glass stir bar. A −40 °C solution of **1** (355 mg, 0.29 mmol) in 2 mL of hexanes was added quickly to this stirring solution. Over the next 5 min, a precipitate begins to form, yielding a slightly cloudy pale pink solution. After stirring for 3 days, the pale pink mixture was passed through a fine frit packed with Celite to yield a tan solid and a dull pink solution. The solution was dried in vacuo to yield a dull pink foam. This residue was triturated with pentane (1 mL × 3), taken up in minimal pentane, passed through a glass fiber filter packed with Celite, and concentrated in vacuo before being placed in a −40 °C freezer. Pink-purple crystals suitable for X-ray diffraction analysis grew overnight and were isolated by decantation and dried in vacuo to yield a light pink powder (234 mg, 73%). ^1^H NMR (500 MHz, C_6_D_6_): δ 21.57 (broad s, 12H, N**CH**_**2**_CH_3_), 9.78 (s, 6H, N**CH**_**2**_CH_2_N), 8.71 (s, 6H, NCH_2_ **CH**_**2**_N), 7.53 (t, 18H, *J* = 5 Hz, NCH_2_ **CH**_**3**_), −1.28 (s, 54H, –C­(**CH**_**3**_)_3_), −182.07 (s, 3H, U–**CH**_**3**_). ^13^C NMR (126 MHz, C_6_D_6_): δ 58.19, 48.53, 21.20, 6.78. ^31^P NMR (203 MHz, C_6_D_6_): 454.14. IR (cm^–1^): *v.* 2968 (s), 2926 (m), 2854 (m), 2686 (vs), 2323 (vw), 1476 (w), 1461 (w), 1387 (m), 1376 (m), 1358 (m), 1266 (m), 1247 (m), 1202 (s), 1146 (s, shoulder), 1118 (vs), 1045 (s), 1023 (vs), 975 (s), 932 (s), 866 (m), 796 (s), 788 (s, shoulder), 704 (s), 642 (s), 593 (w), 514 (s), 500 (s), 449 (m). Elemental analysis % found (calculated) for UP_3_N_12_C_43_H_99_: C, 45.16 (46.31); H, 8.77 (8.95); N, 14.95 (15.07). Carbon was consistently low on multiple analyses.

### [U^4+^(NP­(1,2-bis-^t^Bu-diamidoethane)­(NEt_2_))_4_·C_5_H_12_], **5**

A −40 °C 0.18 M solution of HNP* (0.904 mL, 0.163 mmol) in pentane was added dropwise to a stirring −40 °C solution of **4** (181.81 mg, 0.163 mmol) in 5 mL of pentane equipped with a glass stir bar at room temperature. The transparent pink solution was stirred overnight at room temperature, then dried in vacuo to yield a foamy amorphous solid. The pink residue was taken up in minimal pentane and filtered through a glass fiber filter packed with Celite. The bright pink solution was concentrated and placed in a −40 °C freezer, where blocky pink crystals suitable for crystallographic analysis grew overnight. These crystals were isolated by decantation, washed once with cold pentane, and dried in vacuo to afford the target compound as pink powder (221 mg, 98%). ^1^H NMR (500 MHz, C_6_D_6_): δ 4.80 (s, 16H, N**CH**_**2**_CH_3_), 4.05 (s, 8H, N**CH**_**2**_CH_2_N), 2.26 (s, 8H, s, 8H, NCH_2_ **CH**_**2**_N), 1.26 (t, 24H, *J* = 5 Hz, NCH_2_ **CH**_**3**_), 1.21 (m, 4H, Pentane (CH_3_ **CH**_**2**_)_2_CH_2_), 1.10 (broad s, 2H, Pentane (CH_3_CH_2_)_2_ **CH**_**2**_), 0.87 (t, 6H, *J* = 10 Hz, Pentane-**CH**_**3**_), 0.70 (s, 72H, –C­(**CH**_**3**_)_3_). ^13^C NMR (126 MHz, C_6_D_6_): δ 52.57, 39.42, 34.45, 22.73, 14.28, 13.97, 13.51. ^31^P NMR (203 MHz, C_6_D_6_): 357.45. IR (cm^–1^): 2968 (m), 2953 (m, shoulder), 2926 (m, shoulder), 2914 (m), 2900 (m, shoulder), 2850 (m), 2686 (vw), 1980 (vw), 1473 (w, shoulder), 1459 (w), 1387 (m), 1377 (m), 1358 (m), 1266 (m), 1249 (m), 1227 (m), 1208 (s), 1194 (s), 1150 (s), 1109 (vs), 1056 (vs), 1048 (s, shoulder), 1021 (vs), 978 (s), 932 (s), 868 (m), 796 (s), 778 (w), 729 (vw), 702 (s), 636 (s), 597 (w), 517 (s), 500 (s), 471 (m), 453 (m). Elemental analysis % found (calculated) for UP_4_N_16_C_56_H_128_·C_5_H_12_: C, 49.94 (50.26); H, 9.58 (9.54); N 15.5 (15.37).

### [U^4+^(NP­(1,2-bis-^t^Bu-diamidoethane)­(NEt_2_))_3_(Et_2_O)] [(BArF_24_)], **6**

**3** (222.7 mg, 0.19 mmol) was dissolved in 2 mL of DFB in a 20 mL vial. In another 20 mL vial with a PTFE stir bar, Fc­(BArF_24_) (196 mg, 0.19 mmol) was weighed out. Both vials were chilled at −35 °C for 10 min. Solution of **3** was then added to the vial containing Fc­(BArF_24_), at which point the reaction immediately turned brown with subtle shade of red. The reaction mixture was stirred at RT for 20 min. Subsequently, the reaction mixture was dried in vacuo to yield a brown solid. This brown solid was washed with pentane (5 mL × 3) three times to remove ferrocene. The brown solid is not soluble in pentane, while ferrocene dissolves in pentane to yield a tan yellow solution. After three washes, the next aliquot of pentane added remained colorless, indicating that the wash is sufficient. The brown residue remaining in the vial was dried in vacuo, yielding brown solids. This brown solid was then dissolved in 3 mL of Et_2_O and stirred for 1 h at RT with a PTFE stir bar, yielding a brown solution (no more shade of red). The reaction mixture was filtered through Celite pipet filter, then concentrated to 1 mL. Brown SC-XRD quality crystals were obtained upon cooling at −35 °C for 16 h. The brown crystals were isolated by decantation and dried in vacuo to yield a dark brown powder (85 mg, 18%). ^1^H NMR (400 MHz, C_6_D_6_): δ 16.72 (s, br, 12H, N**CH**_**2**_CH_3_), 10.44 (s, 6H, N**CH**_**2**_CH_2_N), 8.11 (s, 4H,BArF_24_-**Hp**), 7.81 (s, 8H, BArF_24_-**Ho**), 5.18 (s, 18H, NCH_2_ **CH**_**3**_), 3.26 (s, 4H, Diethyl Ether-**CH**_**2**_), 1.05 (s, 6H, diethyl ether**-CH**_**3**_), 0.38 (s, 54H, –C­(**CH**_**3**_)_3_), −0.48 (m, 6H, N**CH**_**2**_CH_2_N). ^13^C NMR (101 MHz, C_6_D_5_Cl): δ 123.05, 117.18, 58.54, 48.12, 19.09, 15.49. ^31^P NMR (162 MHz, C_6_D_5_Cl): δ 372.91. IR (cm^–1^): 2978 (m), 2869 (w), 2156 (w), 1610, (w), 1465 (w), 1381 (w), 1351 (m), 1273 (s), 1207 (m), 1124 (s), 1080 (s), 885 (m), 838, (m), 804 (m), 744 (w), 711 (m), 682 (m), 668 (m), 649 (w), 516 (m), 501 (m), 474 (w), 448 (w), 405 (w). Elemental analysis % found (calculated) for C_78_H_118_N_12_OP_3_BF_24_U: C, 45.96 (45.98); H, 5.70 (5.84); N, 8.40 (8.25).

### In Situ Synthesis of [U^4+^H­(NP­(1,2-bis-^t^Bu-diamidoethane)­(NEt_2_))_3_], **7-H**

**3** (131.5 mg, 0.11 mmol) was dissolved in 2 mL of Et_2_O in a 20 mL vial equipped with a PTFE stir bar. The solution was subsequently cooled in the cold well to <−40 °C. A balloon was filled to ∼5 in. and while holding the neck of the balloon to prevent gas from escaping, it was attached to the vial. The reaction was stirred for 2 h, while keeping a temperature <−20 °C. Note that at cold well temperatures lower than −40 °C, the reaction does not proceed as rapidly. The reaction color still remains yellow. At which point, the reaction mixture was dried in vacuo, yielding a brown, bubbly solid. The bubbly solid was triturated with cold pentane 3 times. NMR was acquired by dissolving the crude material in cold C_6_D_6_ in a J-Young tube equipped with Teflon tube. The J-young tube was kept at LN_2_ temperature during transport to the NMR facility and warmed up to room temperature right before data collection, at which point the data were collected at room temperature. The rest of the crude materials were dissolved in 1 mL of cold pentane and concentrated to 0.5 mL for crystallization at −35 °C overnight. Yellow, SC-XRD quality crystals formed; however, bulk isolation of this complex was not successful due to the reaction yielding mixtures of the starting material, **7-H**, and the decomposition product, **5.** ^31^P NMR (162 MHz, C_6_D_6_): δ 455.90 (*d*, *J*_P–P_ = 205.3 Hz), observed in a mixture with **3** and **5**.

### In Situ Synthesis of [U^4+^D­(NP­(1,2-bis-^t^Bu-diamidoethane)­(NEt_2_))_3_], **7-D**

This intermediate was also prepared for NMR analysis from **3** (89 mg, 0.076 mmol) following synthetic methods and reaction conditions described for **7-H**, with the only difference being that D_2_ gas was used in place.

## Supplementary Material



## Notes

Detailed experimental conditions, NMR, IR, cyclic voltammogram, SC-XRD, and UV–vis NIR (PDF)
