# Supplementary material for: Synthesis and Reactivity of Heteroleptic U4+ Alkyl, Benzyl, and Hydride Imidophosphorane Complexes
Source: Inorg Chem. 2026 Jun 17;65(26):14787–95. doi: 10.1021/acs.inorgchem.6c01434 (PMC13343460; doi:10.1021/acs.inorgchem.6c01434)
Supplement: Supplementary file 1 [file ic6c01434_si_001.pdf]

Supporting Information for:

Synthesis and Reactivity of Heteroleptic U^{4+} Alkyl, Benzyl, and Hydride Imidophosphorane Complexes

Haruko Tateyama[†], Maximilian G. Bernbeck[†], Tyler-Rayne Nero[†], Grant R. Wilkinson[†], Kaitlyn S. Engle[†], Gabriel M. Betts[†] and Henry S. La Pierre[†,‡,||*]

[†]School of Chemistry and Biochemistry, Georgia Institute of Technology, Atlanta, Georgia 30332-0400,United States

[‡]Nuclear and Radiological Engineering Program, Georgia Institute of Technology, Atlanta, Georgia 30332-0400, United States

[||]Physical Sciences Division, Pacific Northwest National Laboratory, Richland, Washington 99352, United States

Email: hsl@gatech.edu

Contents

General Considerations 2

NMR Spectroscopy 3

UV-vis NIR Electronic Absorption Spectra 18

Infra-Red Spectrum of 7-H and 7-D 21

Electrochemistry 22

Crystallographic Analyses 24

References 94

General Considerations

NMR spectra were obtained on a Bruker Avance III 400 MHz or 500 MHz spectrometer at 298 K. ^{1}H NMR spectra and $^{13}C\{^{1}H\}$ spectra are referenced to the residual ^{1}H resonances of the deuterated solvent unless otherwise noted. $^{31}P\{^{1}H\}$ NMR spectra are referenced using an absolute reference to H_3PO_4. Peak position is reported, followed by peak multiplicity, integration value, and assignment where applicable. Abbreviations for the peak multiplicity are as follows: s (singlet); d (doublet); t (triplet); m (multiplet). Infrared (IR) spectroscopy was conducted on a Bruker ALPHA FTIR Spectrometer from 400 to 4000 cm^{-1}, using an ATR attachment inside of a N_2 glovebox. The intensities of the peaks are reported using the following abbreviations: vw (very weak); medium (medium); s (strong); vs (very strong); br (broad). UV-vis NIR spectroscopy was conducted on a Hitachi UH4150 UV-vis-NIR scanning spectrophotometer from 1000 nm to 300 nm. Characterization was performed in small-volume screw cap quartz cuvettes (Starna Scientific) with a 1 cm path length. SC-XRD structures were performed at the Georgia Institute of Technology X-ray Crystallography Facility, on a Bruker D8 Venture diffractometer.

Electrochemical analysis: $[^{n}Bu_4N][PF_6]$ (Oakwood) was recrystallized three times from absolute ethanol, then dried under vacuum at 100 °C prior to use. $[^{n}Bu_4N][BPh_4]$ was prepared as previously reported, and dried under vacuum at 85 °C for 24 h prior to use. Ferrocene used for reference was sublimed before use. Electrochemical data were measured using a Pine WaveDriver 20 Bipotentiostat/Galvanostat. Measurements were performed in a glovebox under an atmosphere of N_2 with a glassy carbon working electrode (3 mm diameter), a bare Ag wire reference electrode in a fritted capillary filled with the corresponding electrolyte solution, and a platinum wire counter electrode at ambient temperature (~25 °C, 298 K). The cell was constructed in a 20 mL scintillation vial The fritted capillary was stored in electrolyte solution when not in use, and the inner solution was replaced with fresh electrolyte solution prior to use. The glassy carbon and Ag wire electrodes were polished before use. Electrolyte solutions were prepared in 1,2-difluorobenzene. Measurements were made in positive feedback iR compensation mode (~500 Ω for 0.1 M $[nBu_4N][BPh_4]$ in THF, ~800 Ω for 0.1 M $[nBu_4N][PF_6]$ in 1,2-difluorobenzene). Voltammograms were referenced by adding a small amount of ferrocene (0.2 M $[nBu_4N][PF_6]$ in PhF) at the end of each experiment. All potentials are reported vs. Fc+/Fc. CHN analyses for **3** and **4** were conducted at University of California Berkeley Microanalytical Facility (Berkeley, CA). For complexes **1, 2**, **5**, **6** CHN analyses were performed on Exeter Analytical CE-440 elemental analyzer at the University of Iowa MATFab Facility (Iowa City, IA).

NMR Spectroscopy

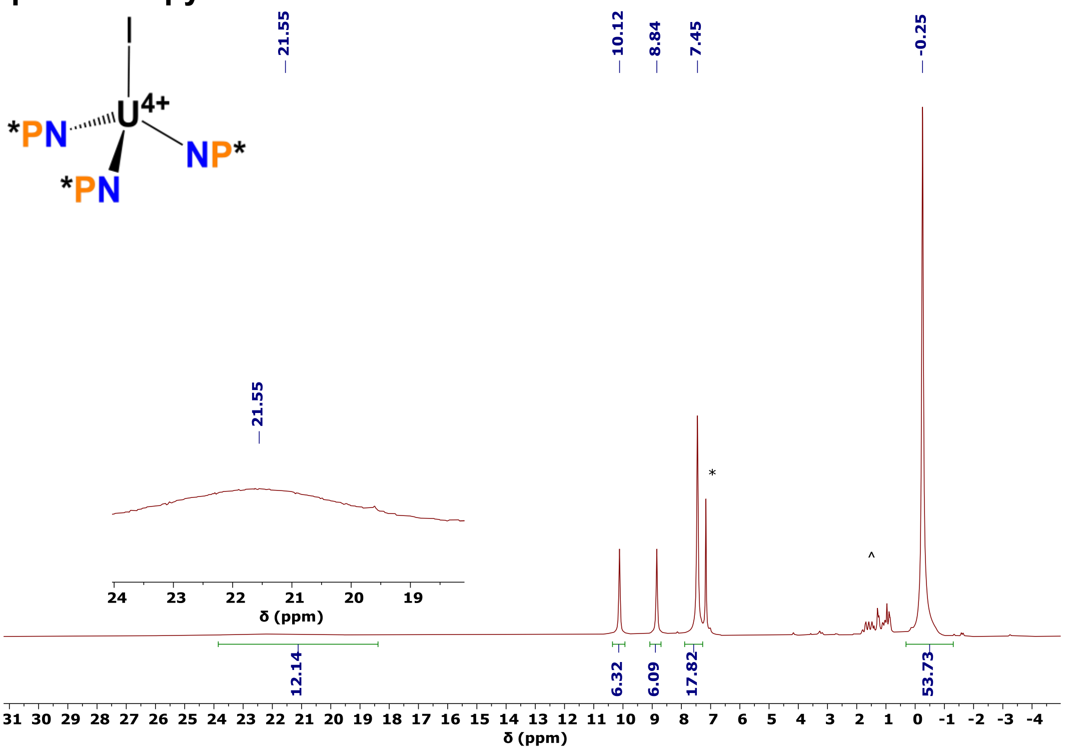

Figure S1. ^{1}H NMR (500 MHz) of **1** in C_6D_6. Solvent residual peak is denoted with *, and impurity is shown in ^.

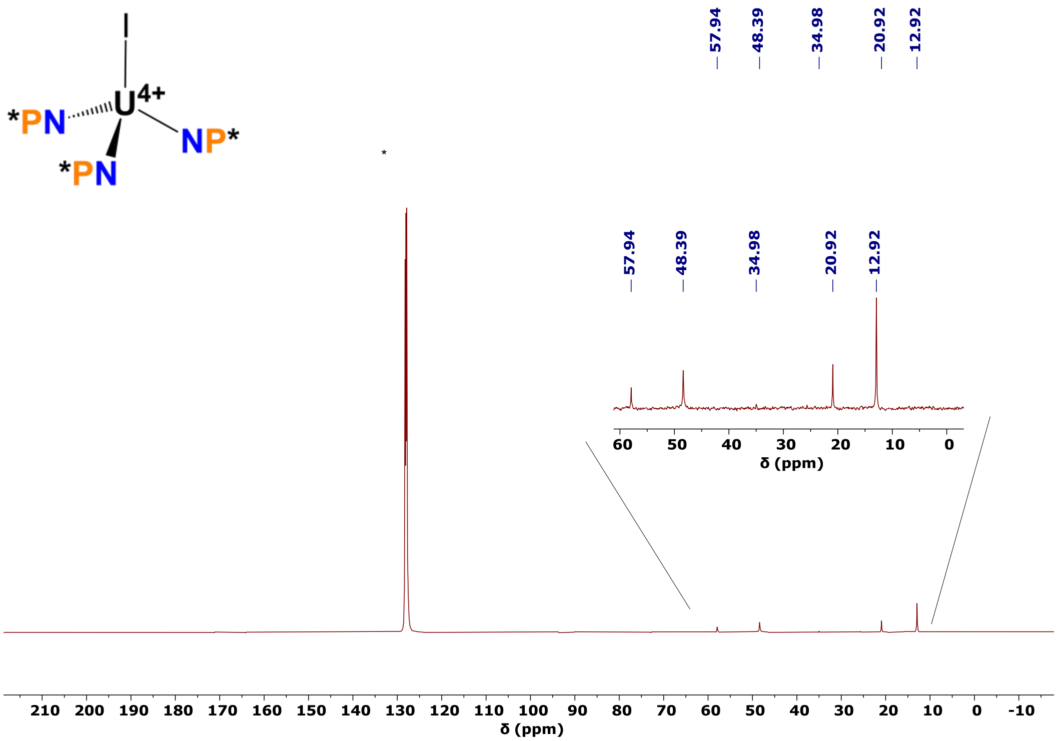

Figure S2. $^{13}C\{^{1}H\}$ NMR (126 MHz) of **1** in C_6D_6. Solvent residual peak is denoted with *.

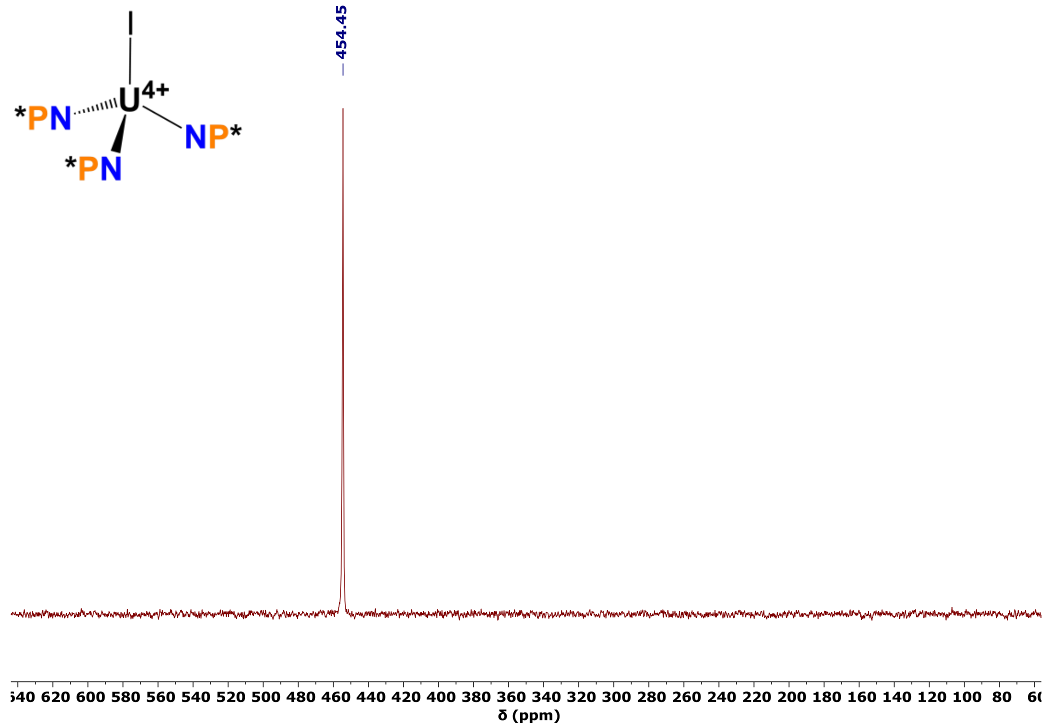

Figure S3. $^{31}P\{^{1}H\}$ NMR (203 MHz) of **1** in C_6D_6.

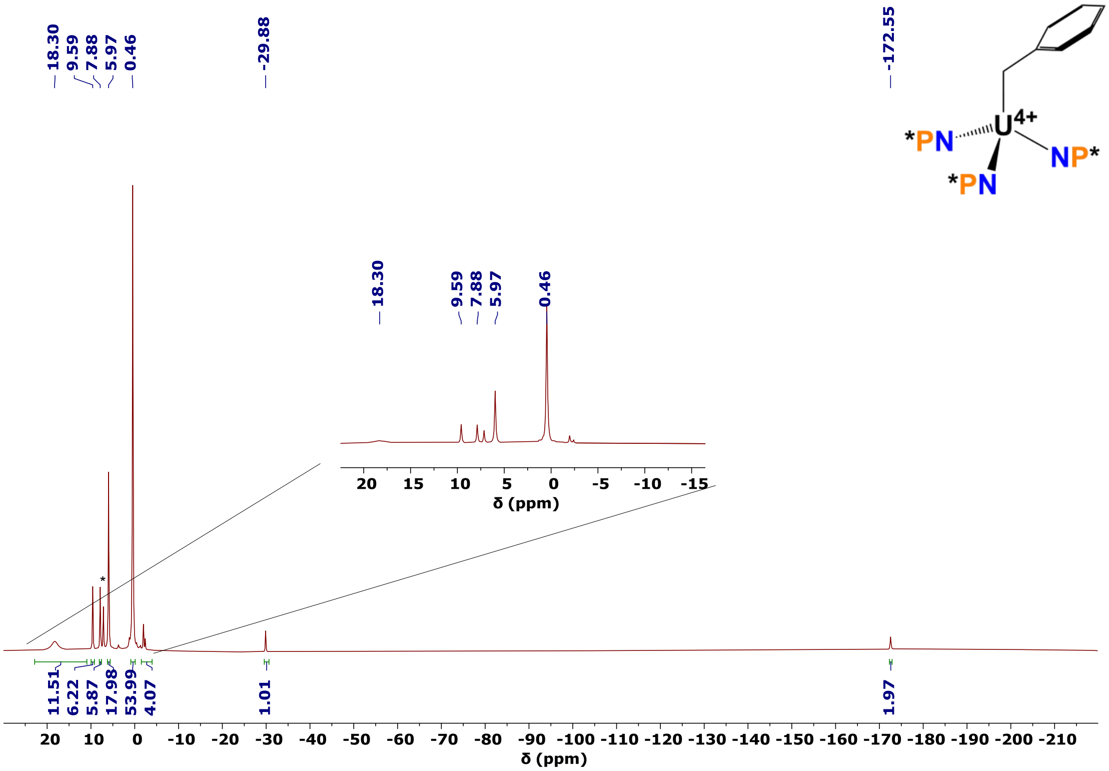

Figure S4. ^{1}H NMR (500 MHZ) of **2** in C_6D_6. Solvent residual peak is denoted with *.

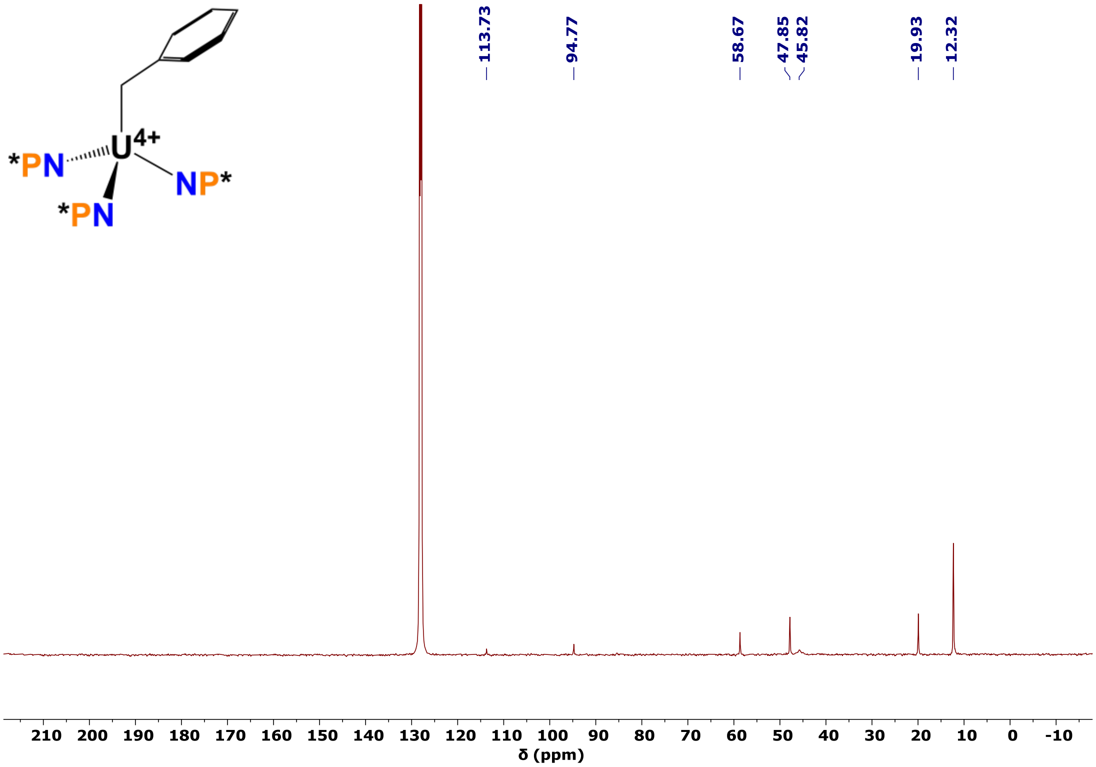

Figure S5. $^{13}C\{^1H\}$ NMR (126 MHz) of **2** in C_6D_6. Solvent residual peak is denoted with *.

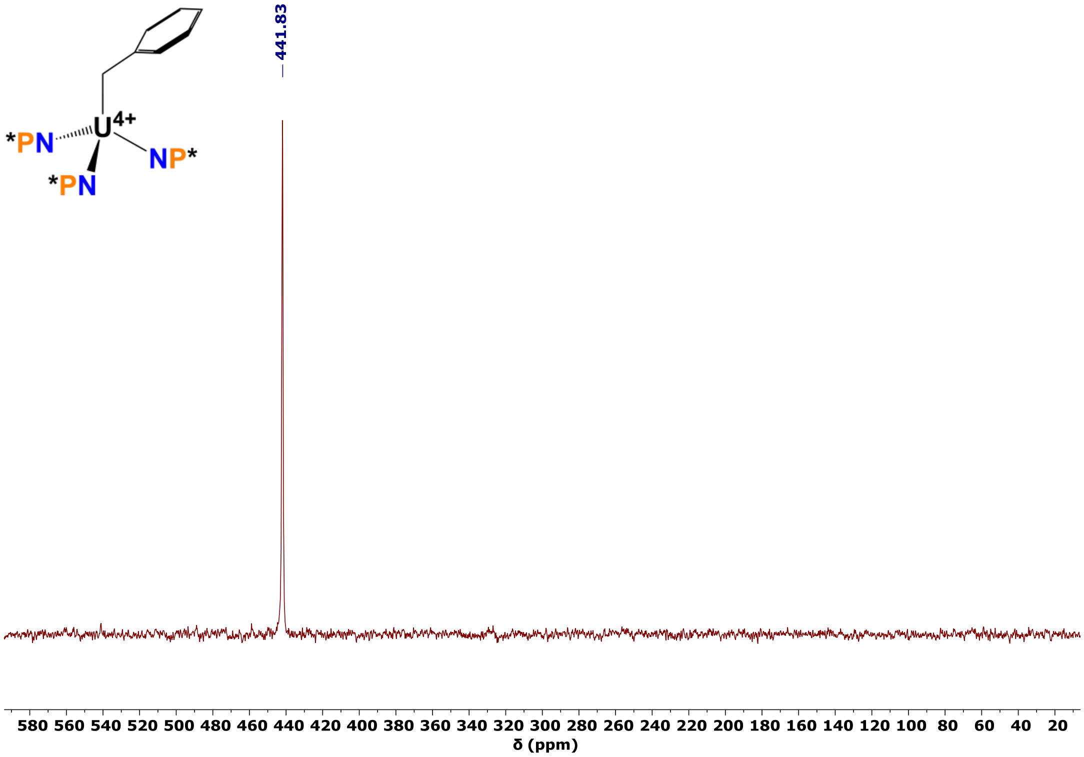

Figure S6. $^{31}P\{^1H\}$ NMR (203 MHz) of **2** in C_6D_6.

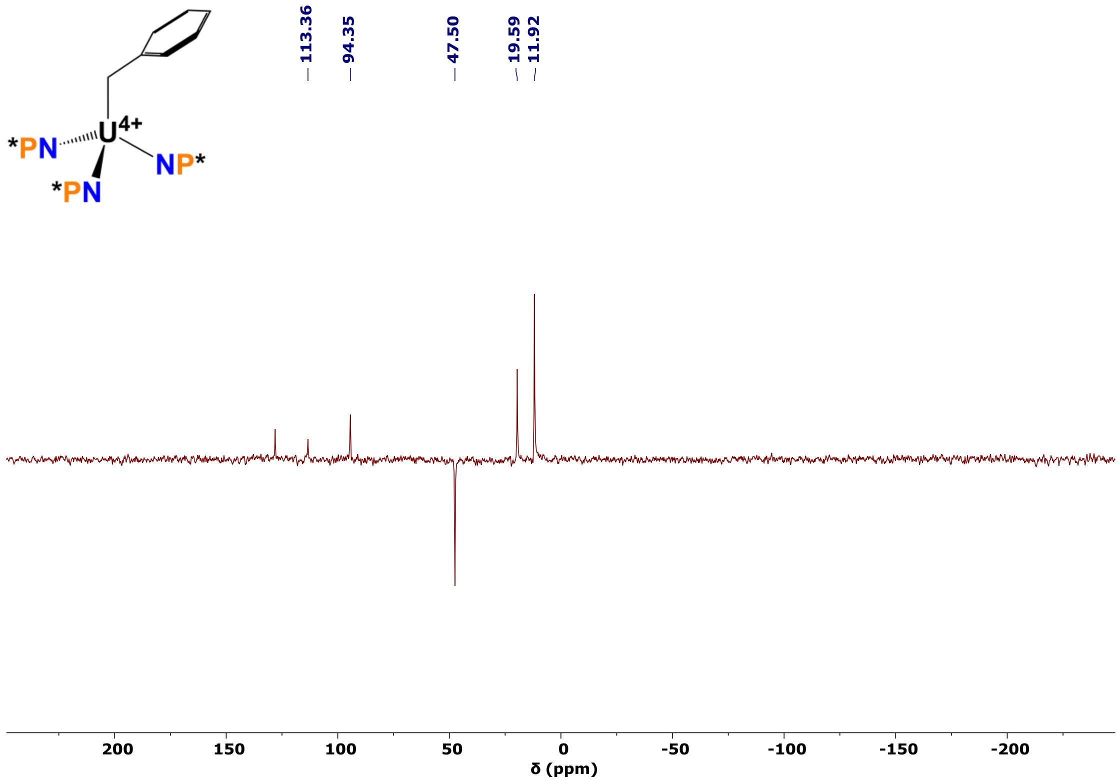

Figure S7. $^{13}C\{^{1}H\}$ DEPT135 NMR of **2 in** C_6D_6.

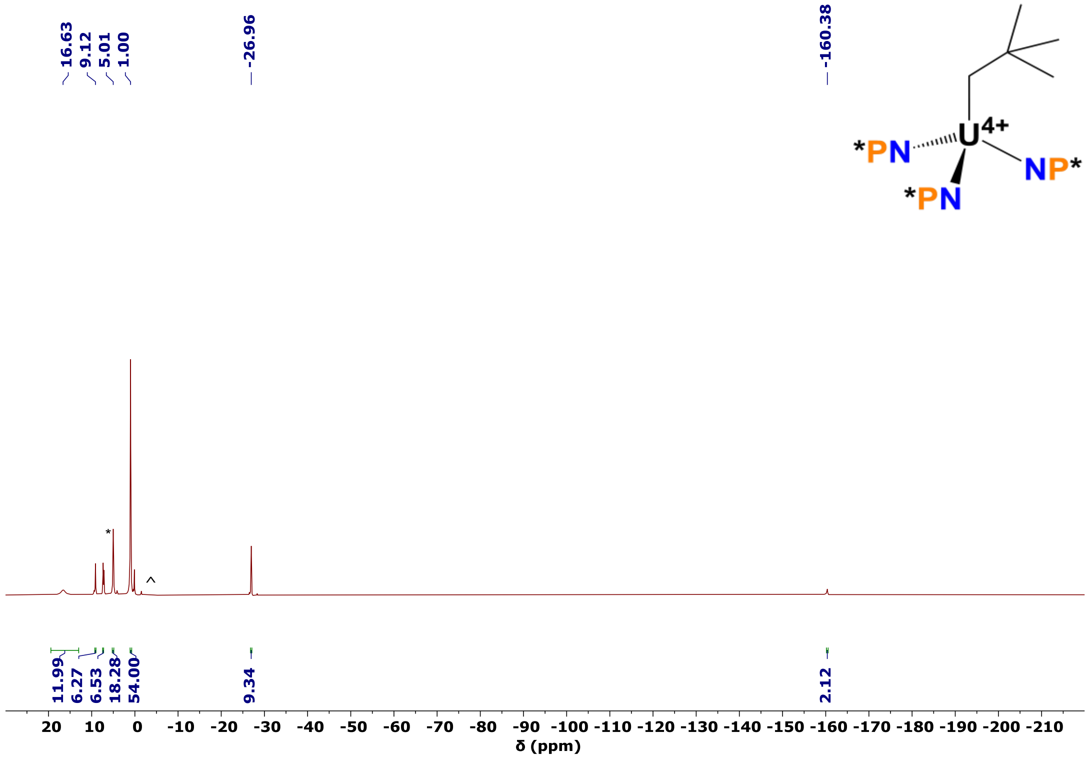

Figure S8. ^{1}H NMR (500 MHz) of **3** in C_6D_6. Solvent residual peak is denoted with * and HMDSO is marked with ^.

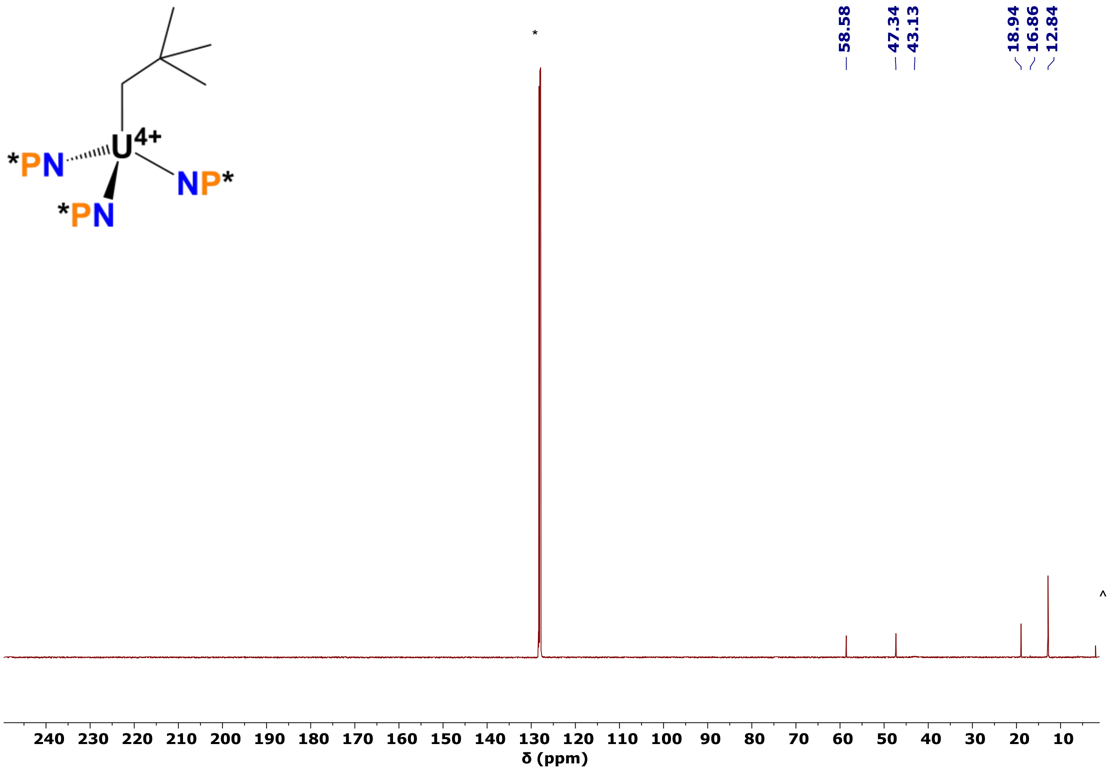

Figure S9. $^{13}C\{^1H\}$ NMR (126 MHz) of **3** in C_6D_6. Solvent residual peak is denoted with * and HMDSO is marked with ^.

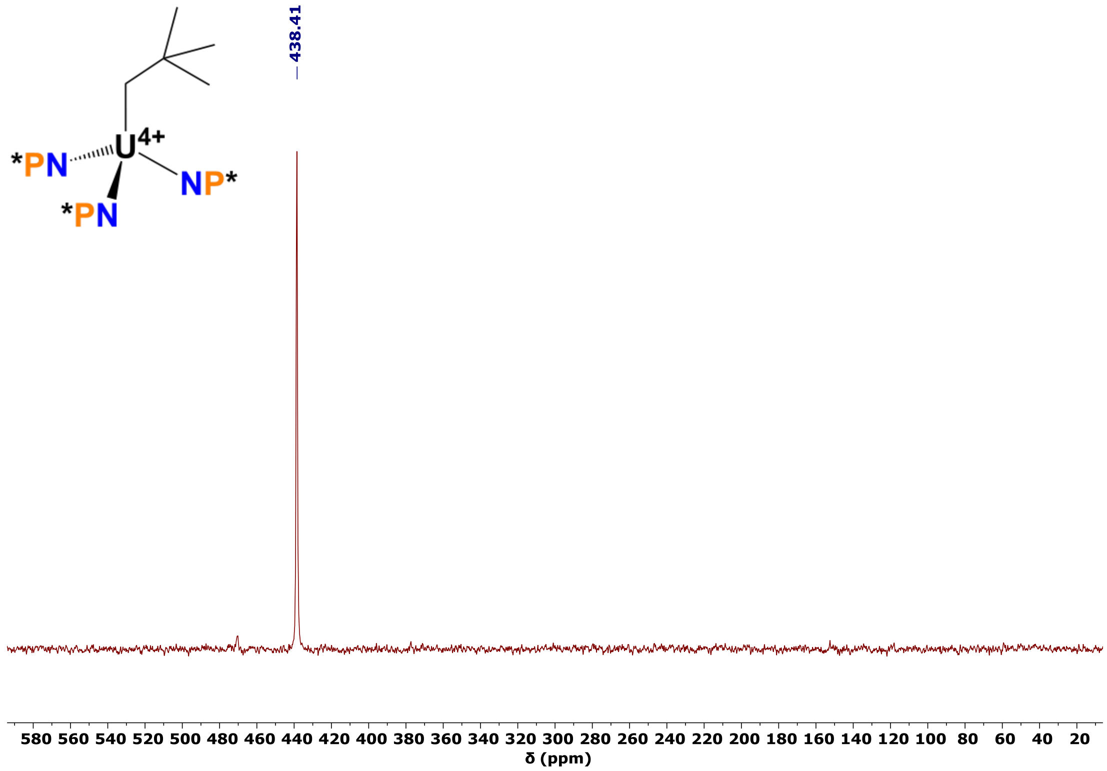

Figure S10. $^{31}P\{^1H\}$ NMR (203 MHz) of **3** in C_6D_6.

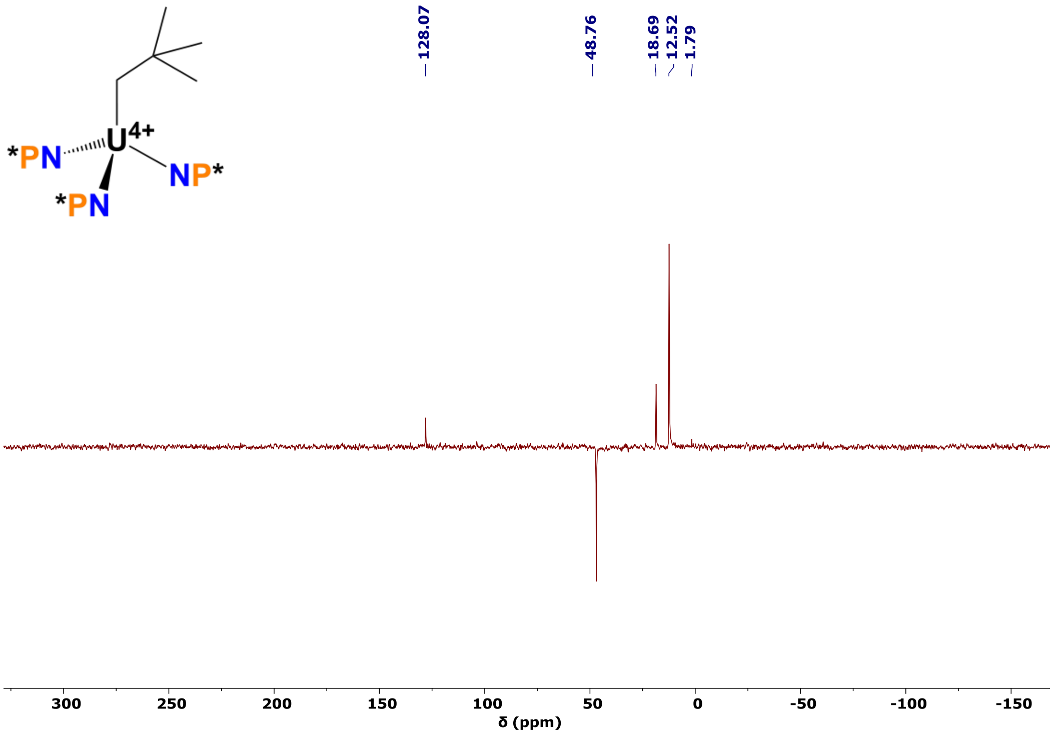

Figure S11. $^{13}C\{^1H\}$ DEPT135 NMR of **3.**

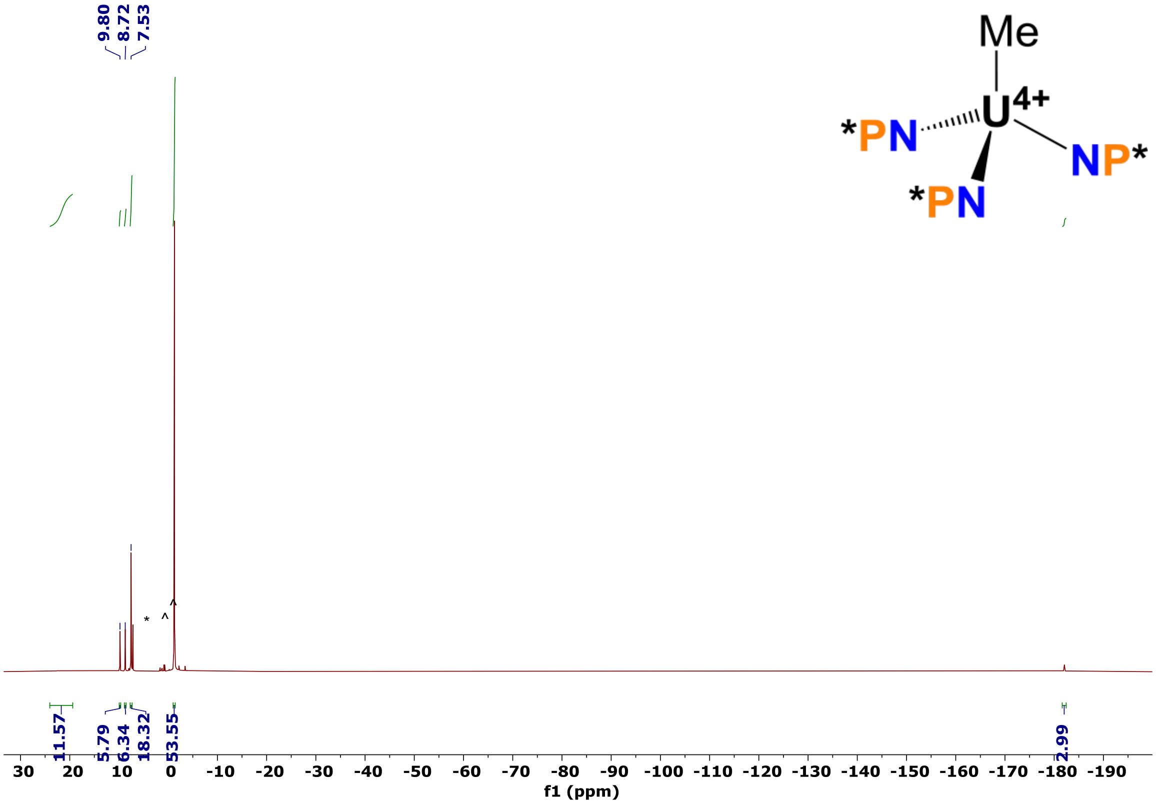

Figure S12. ^1H NMR (500 MHz) of **4** in C_6D_6. Solvent residual peak is denoted with * and hexanes is marked with ^.

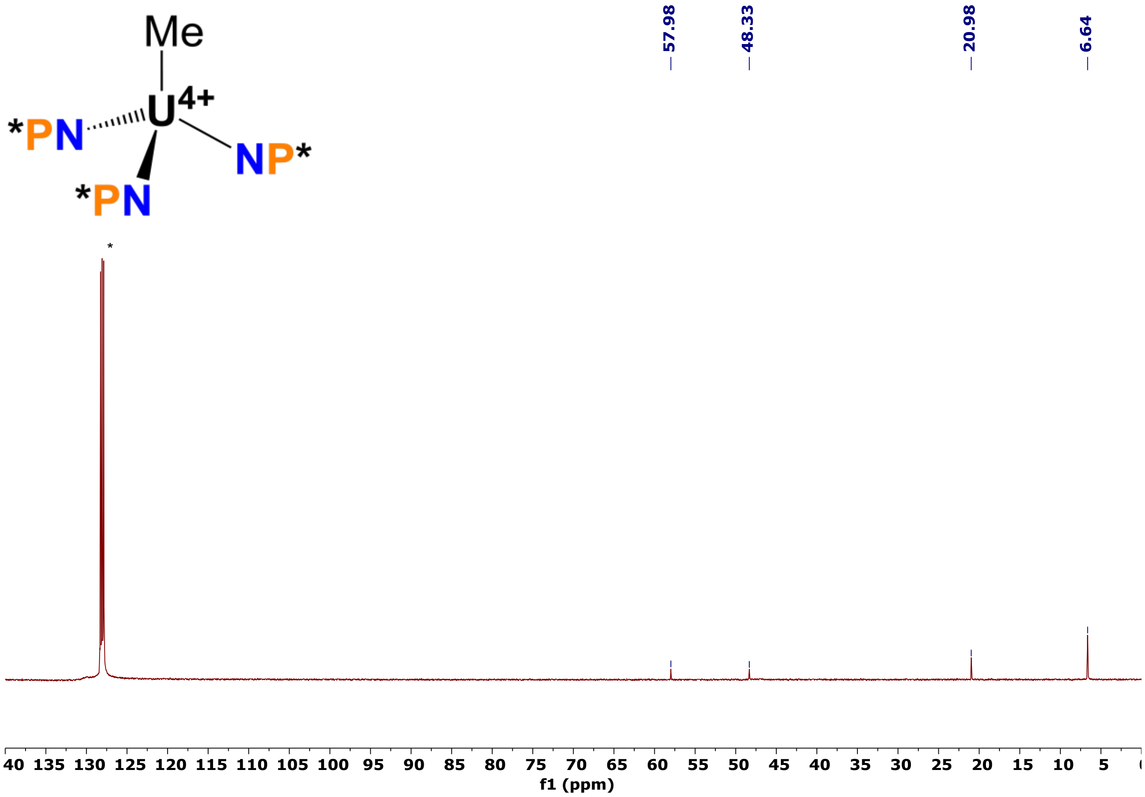

Figure S13. $^{13}C\{^1H\}$ NMR (126 MHz) of **4** in C_6D_6. Solvent residual peak is denoted with *

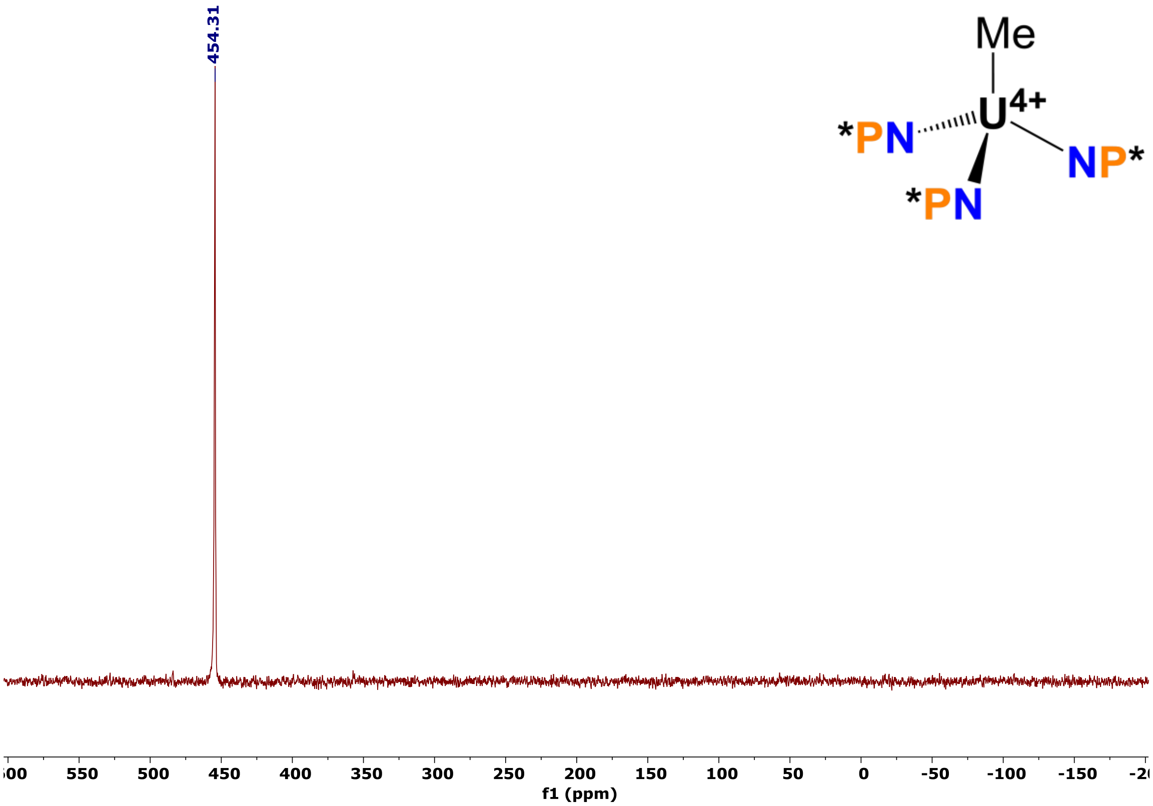

Figure S14. $^{31}P\{^1H\}$ NMR (203 MHz) of **4** in C_6D_6.

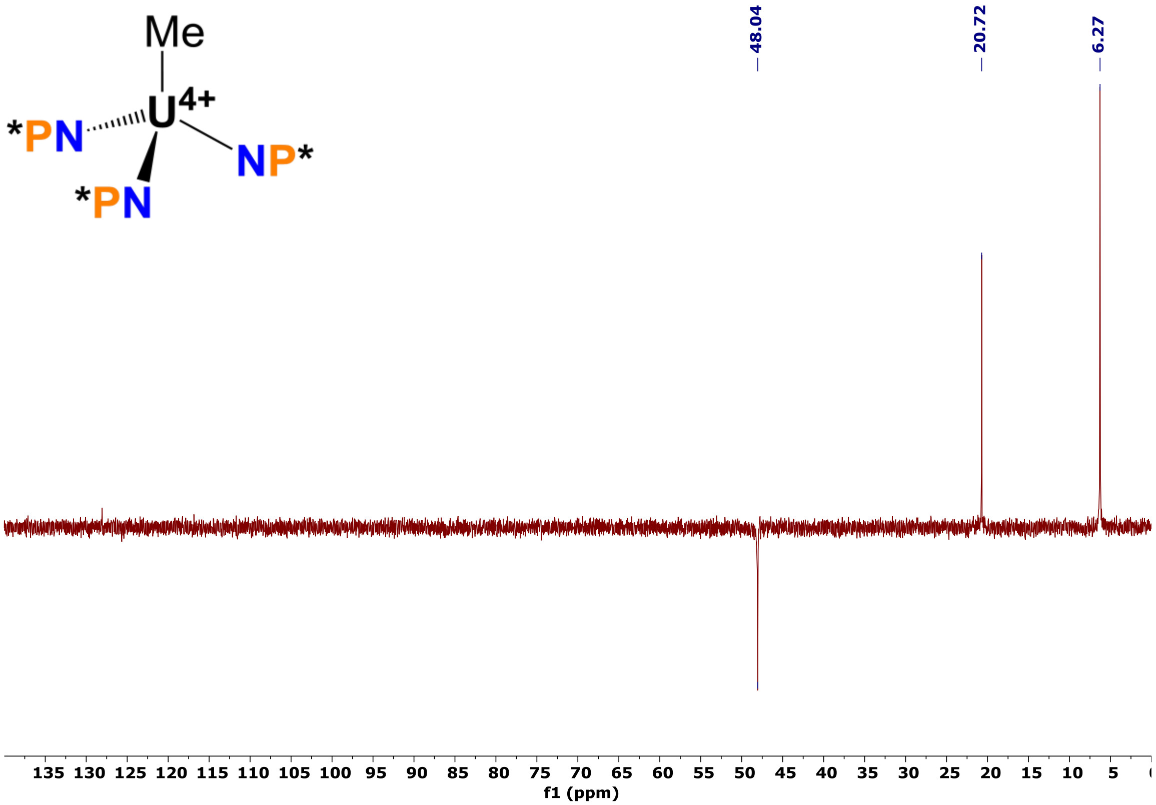

Figure S15. $^{13}C\{^{1}H\}$ DEPT135 NMR of **4.**

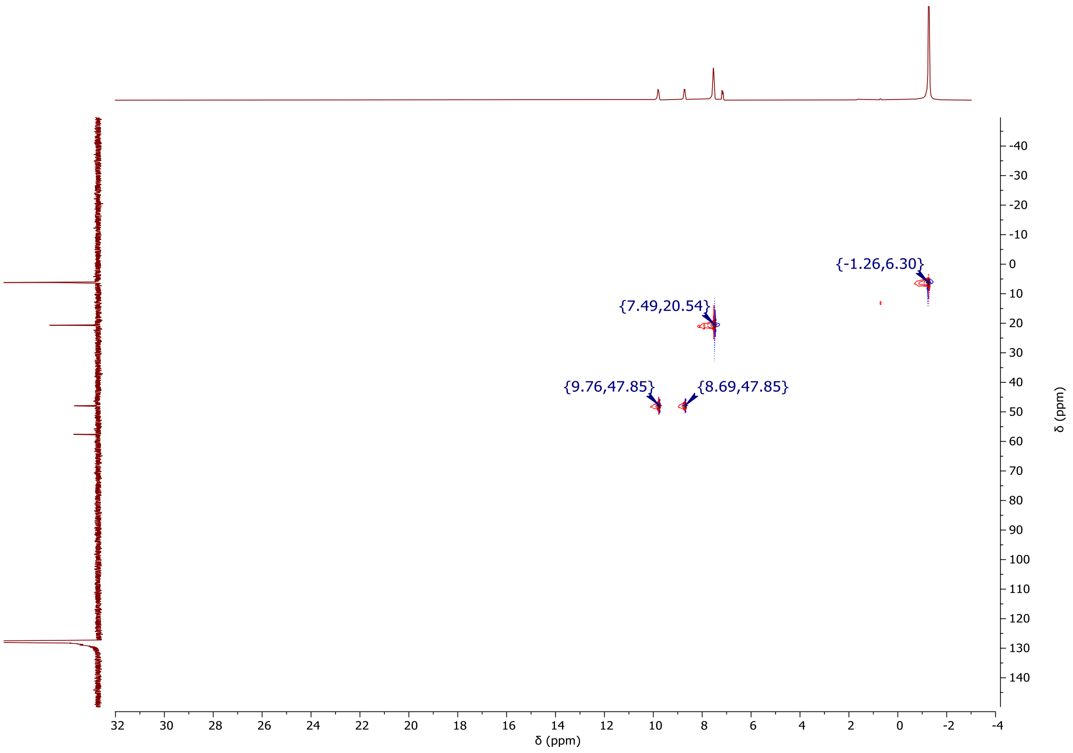

Figure S16. ^{1}H-^{13}C HSQC of **4** in C_6D_6. Residual solvent signals are at 7.16 ppm (^{1}H) and 128.06 ppm (^{13}C).

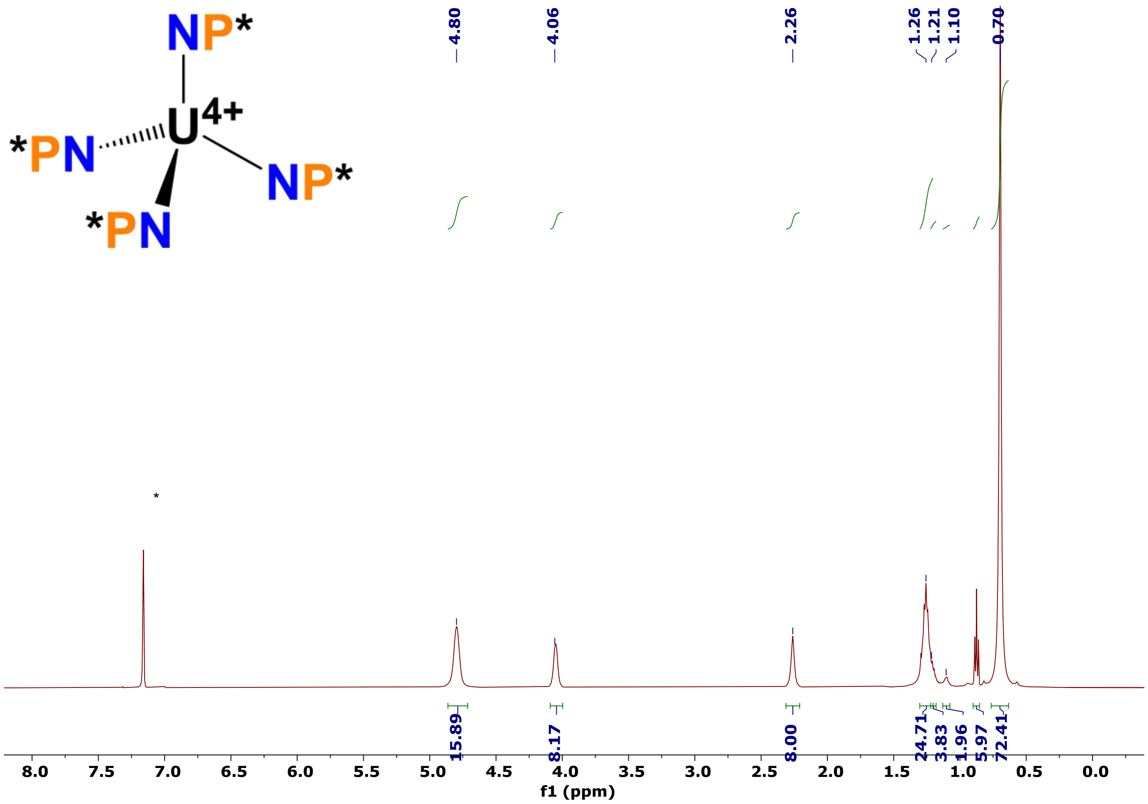

Figure S17. ^{1}H NMR (500 MHz) of **5** in C_6D_6. Solvent residual peak is denoted with *.

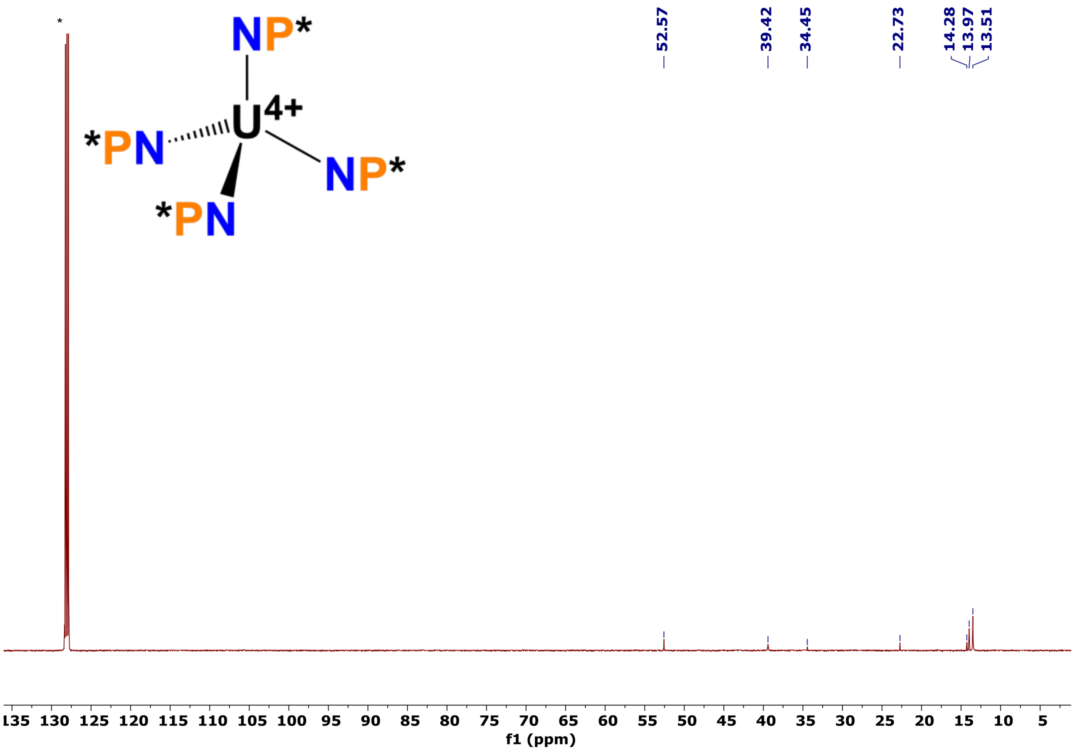

Figure S18. $^{13}C\{^{1}H\}$ NMR (126 MHz) of **5** in C_6D_6. Solvent residual peak is denoted with *.

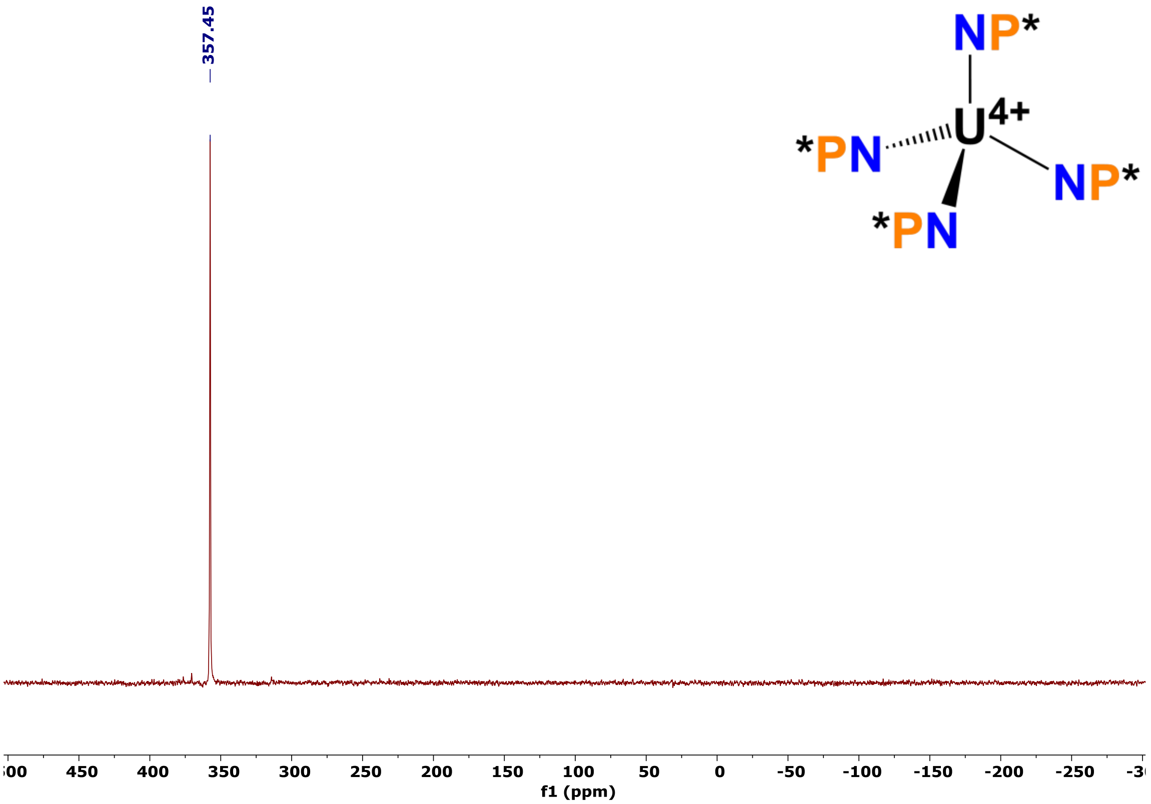

Figure S19. $^{31}P\{^{1}H\}$ NMR (203 MHz) of **5** in C_6D_6

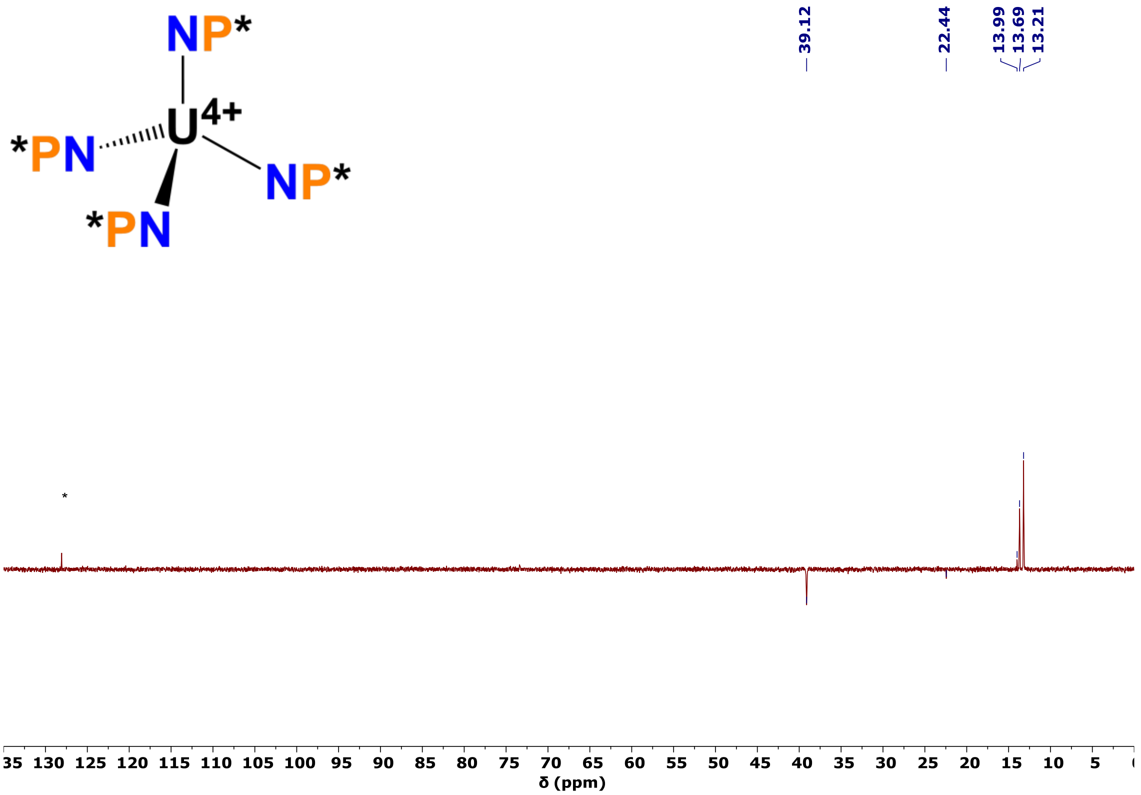

Figure S20. $^{13}C\{^{1}H\}$ DEPT135 NMR of **5.**

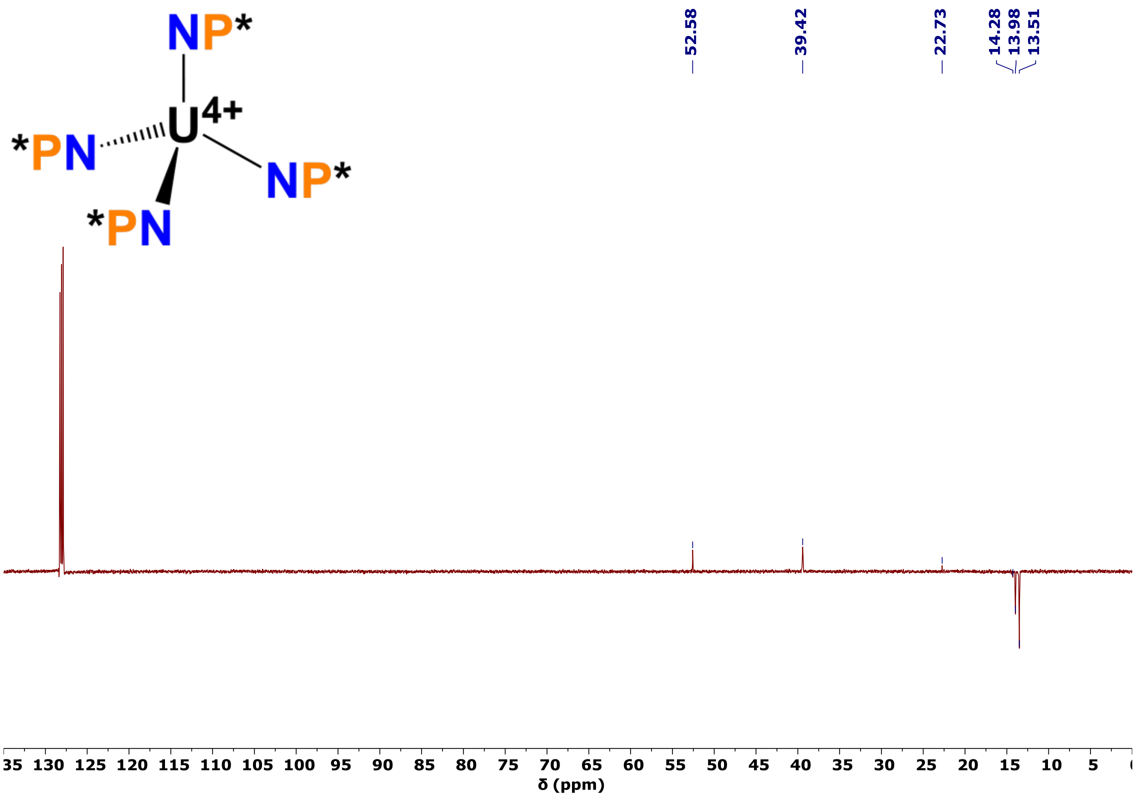

Figure S21. $^{13}C\{^{1}H\}$ DEPT45 NMR of **5.**

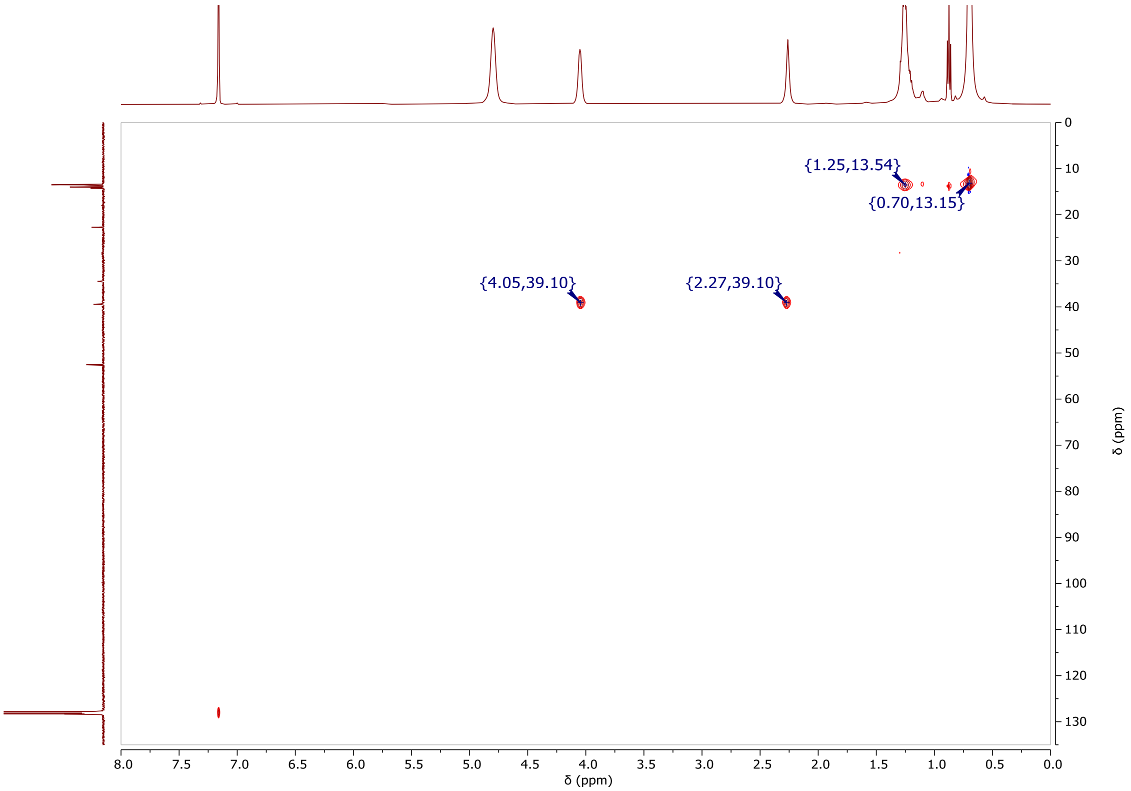

Figure S22. ^{1}H-^{13}C HSQC of **5** in C_6D_6. Residual solvent signals are at 7.16 ppm (^{1}H) and 128.06 ppm (^{13}C).

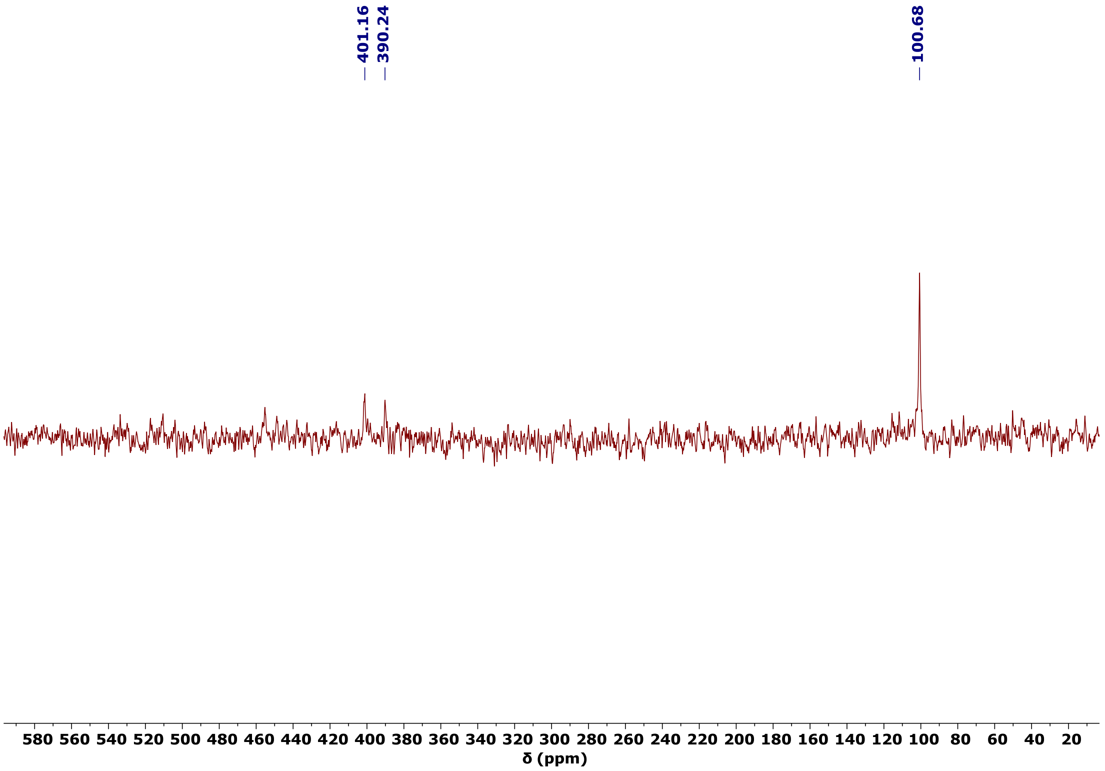

Figure S23. Crude $^{31}P\{^{1}H\}$ NMR (162 MHz, C_6D_6) of the reaction mixture of **3** and Fc{BArF24}.

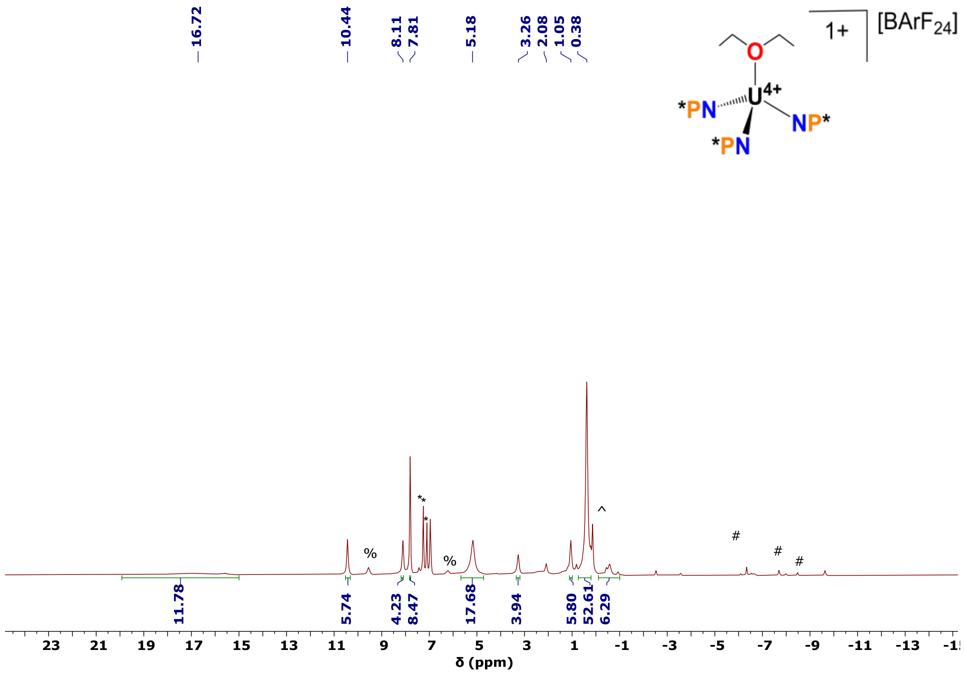

Figure S24 ^{1}H NMR (400 MHz, C_6D_5Cl) of **6** in * denotes residual solvent, % denotes fluorobenzene, ^ denotes silicon grease, # denotes impurities (note that this crystallized sample passed EA).

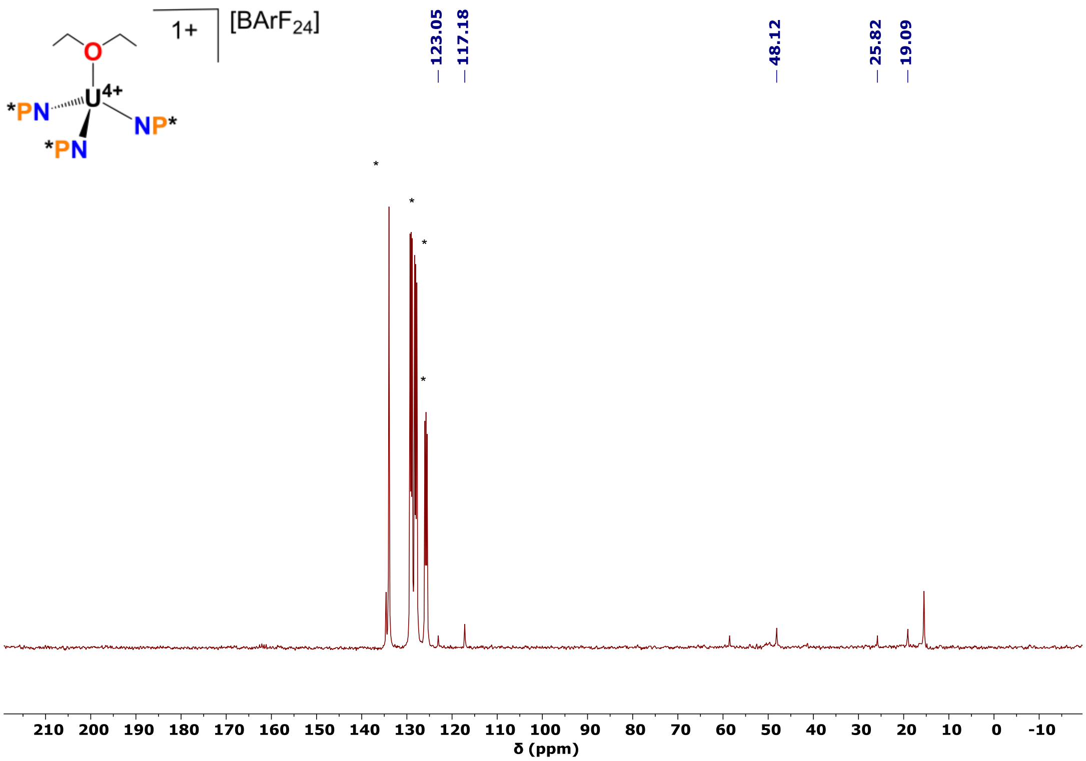

Figure S25. $^{13}C\{^{1}H\}$ NMR (126 MHz) of **6** in C_6D_5Cl. Solvent residual peak(C_6D_5Cl) is denoted with *.

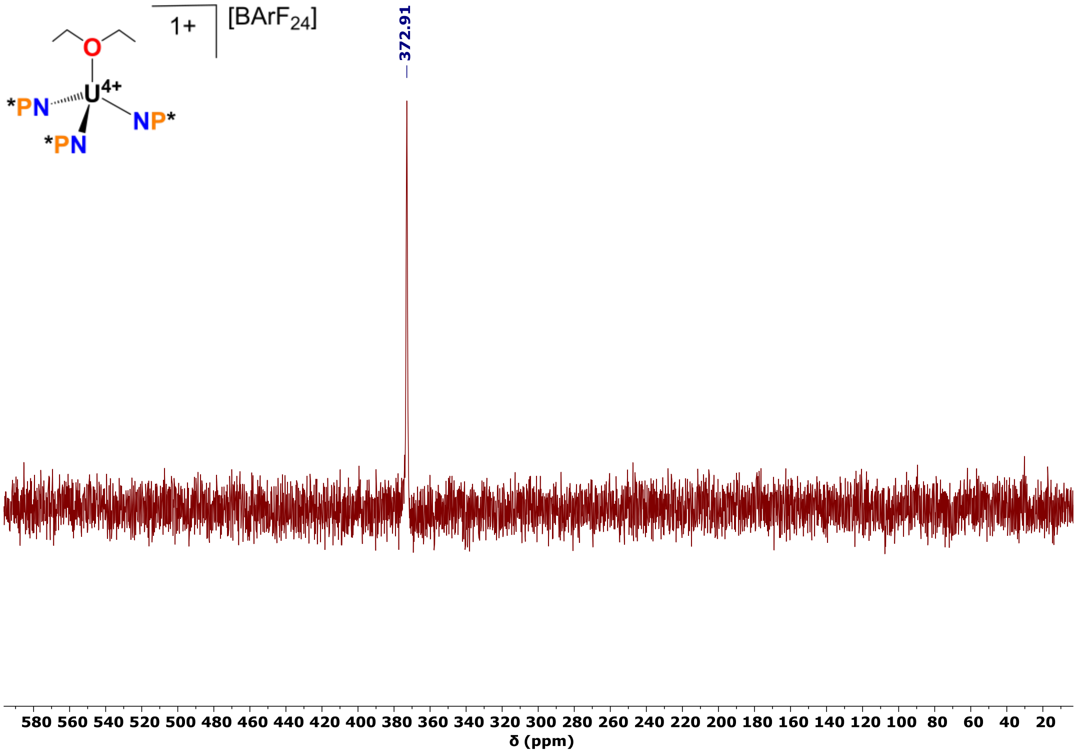

Figure S26. $^{31}P\{^{1}H\}$ NMR (203 MHz) of **6** in C_6D_5Cl.

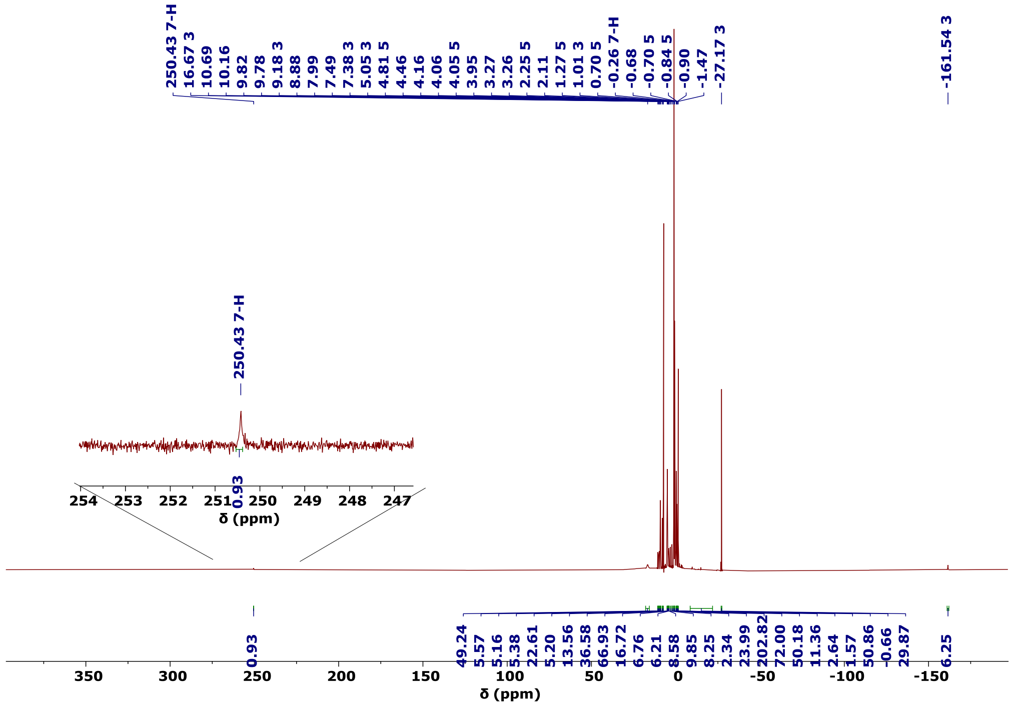

Figure S27. ^{1}H NMR (400 MHz, C_6D_6) of crude reaction, 2 hours after hydrogenation of **3**, yielding a mixture of **3**, **7-H** and **5**. U-H resonance is integrated against the tBu group on the NP* ligand of 7-H, identified at -0.26 ppm to give relative integration.

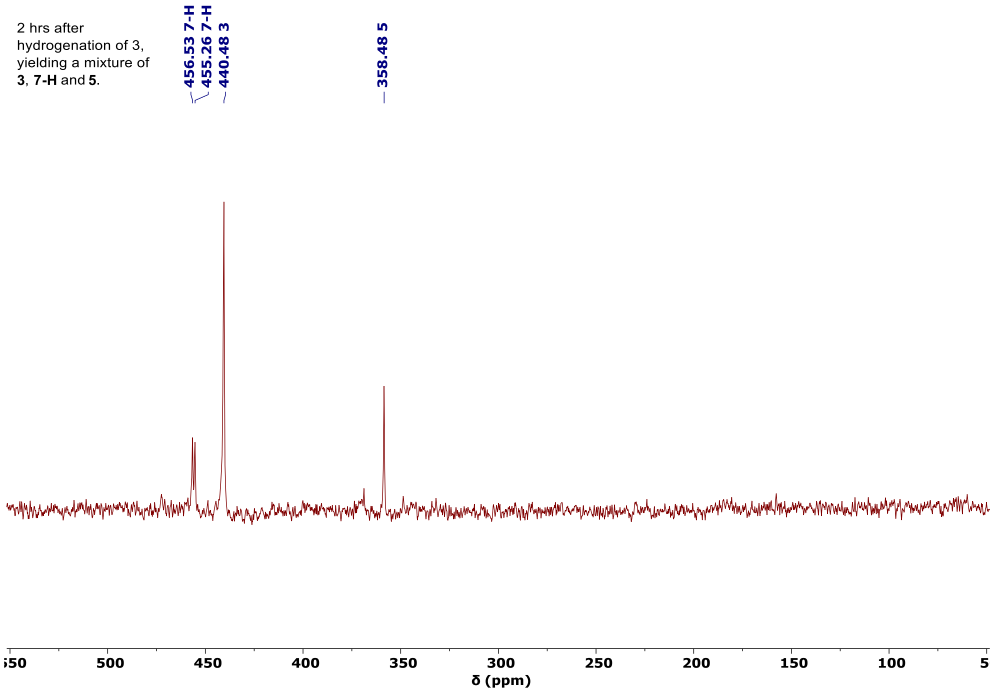

Figure S28. ^{31}P{^{1}H} NMR (162 MHz, C_6D_6) of crude reaction, 2 hours after hydrogenation of **3**, yielding a mixture of **3**, **7-H** and **5**.

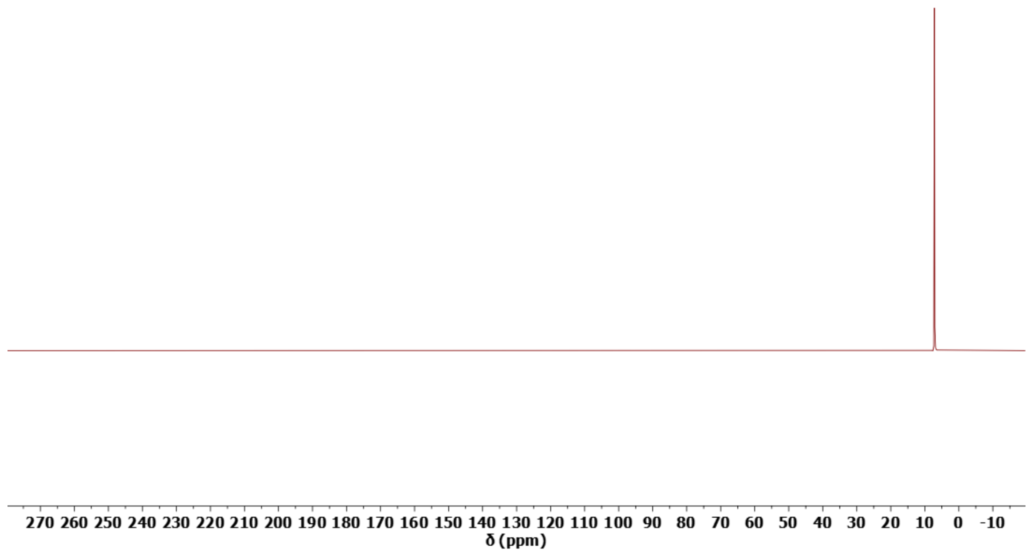

Figure S29. ^{2}H NMR (61 MHz, C_6D_6) of in-situ reaction of **7-D** only showing the residual solvent. Due to the low resolution of ^{2}H compared to ^{1}H, at 6 scans U-D resonance cannot be resolved.

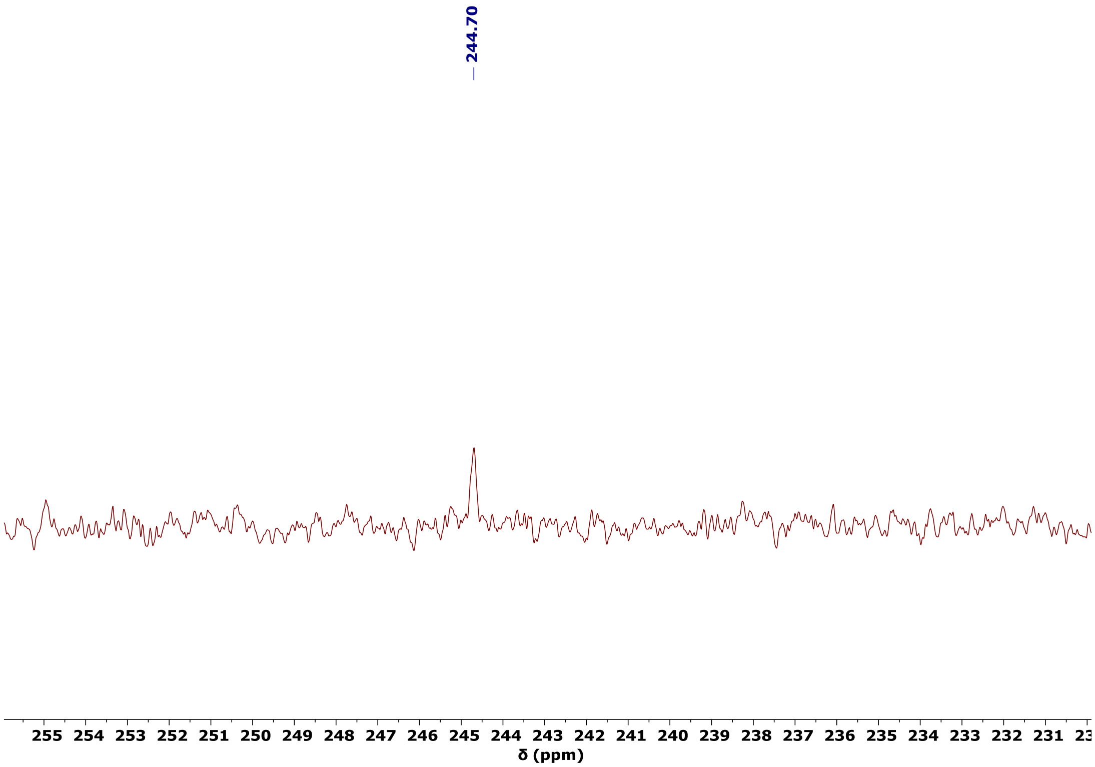

Figure S30. ^{2}H NMR (61 MHz, C_6D_6) of in-situ reaction of **7-D,** collected between 260 and 230 ppm with 1024 scans to show U-D resonance at 244.70 ppm.

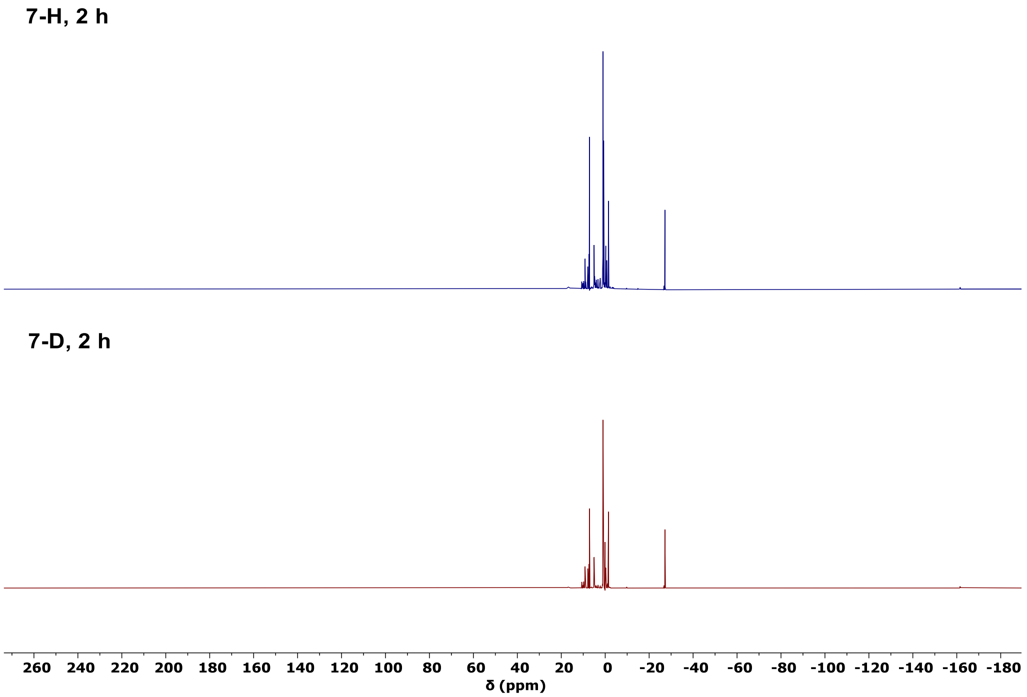

Figure S31. Stacked ^{1}H NMR (400 MHz, C_6D_6) spectra of crude reaction of **7-H** (blue, shown on top) and **7-D** (red, shown on bottom), 2 h after hydrogenation of **3**.

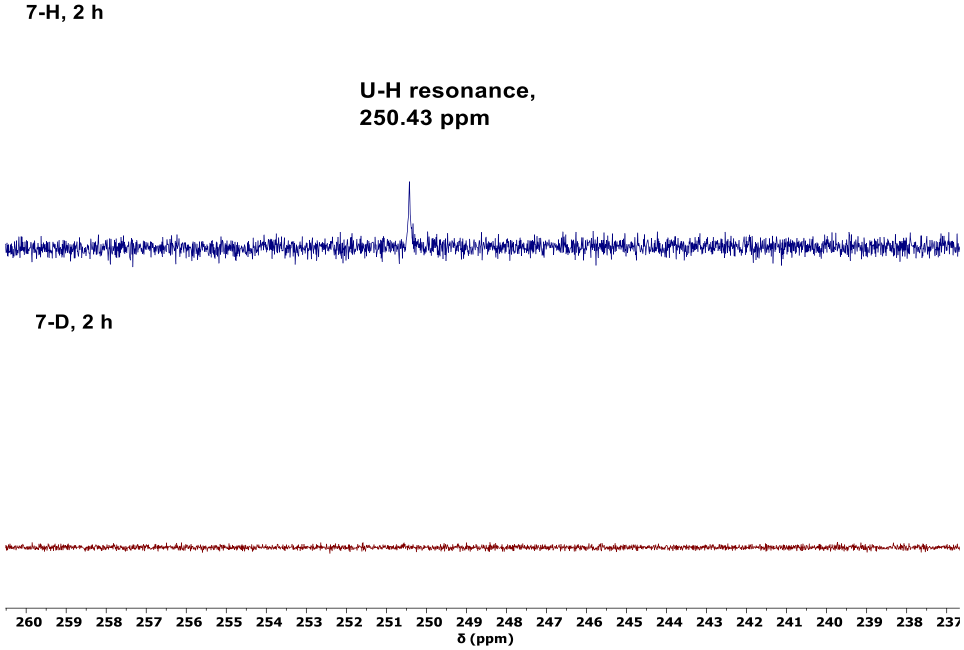

Figure S32. Magnified view of the stacked ^{1}H NMR (400 MHz, C_6D_6) spectra of crude reaction of **7-H** (blue, shown on top) and **7-D** (red, shown on bottom), 2 h after hydrogenation of **3** (expansion of **Figure S31**). U-H resonance at 250. 43 ppm is only observed in **7-H** and not in **7-D**.

UV-vis NIR Electronic Absorption Spectra

UV-vis NIR: Data presented in the main text Figure 3 was collected at a single, higher concentration (5-10 mM) to show the *f-f* transition in complexes 1-5, shown in **Figure S33**. The resulting molar absorptivity coefficient takes into account the premise that the instrument is in the linear range where data was collected, and our analysis only concerns the relative changes across the complexes. **Figure S34** shows the co-plotted UV-vis spectra of complexes 1-5 at a dilute concentration, where a poorly resolved featured in all complexes but **2** around 330-380 nm.

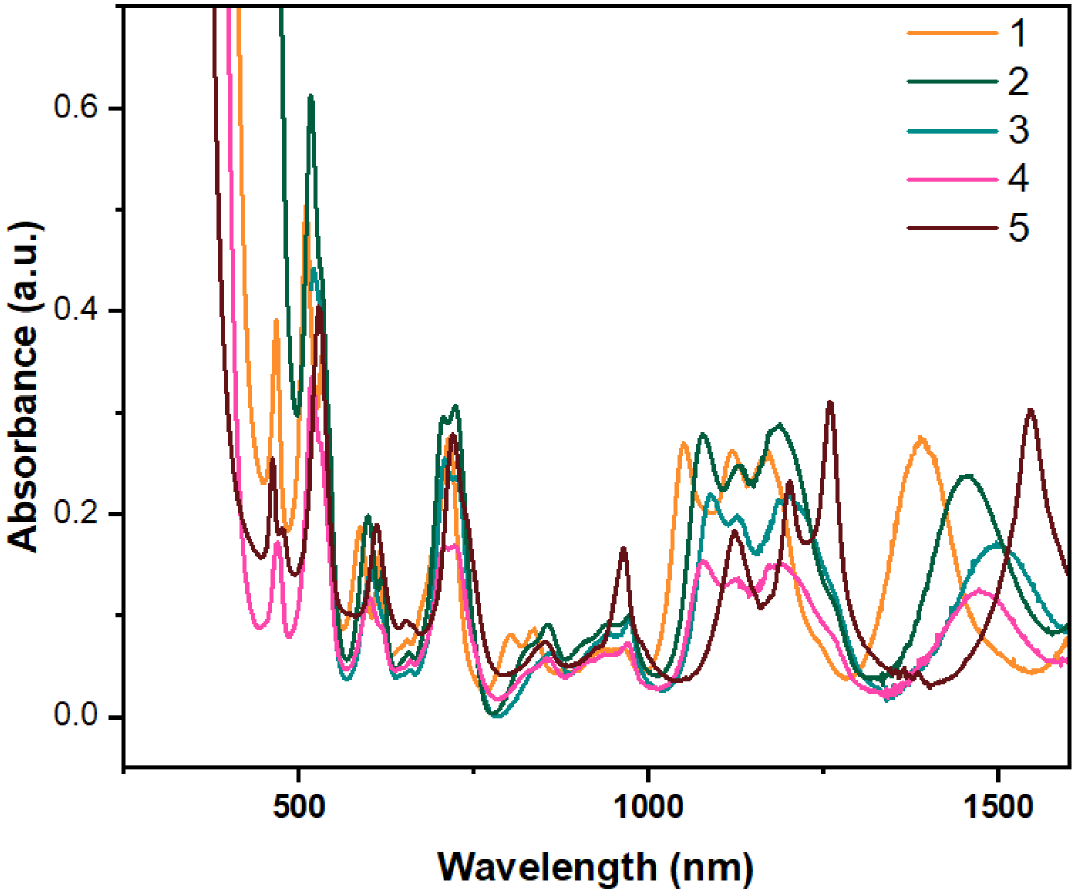

Figure S33. Co-plotted UV-vis NIR spectra of complexes **1** (8.0 mM in THF), **2** (9.1 mM in THF), **3** (8.9 mM in hexanes), **4** (5.8 mM in THF), **5** (6.3 mM in THF) to show f-f transition**.**

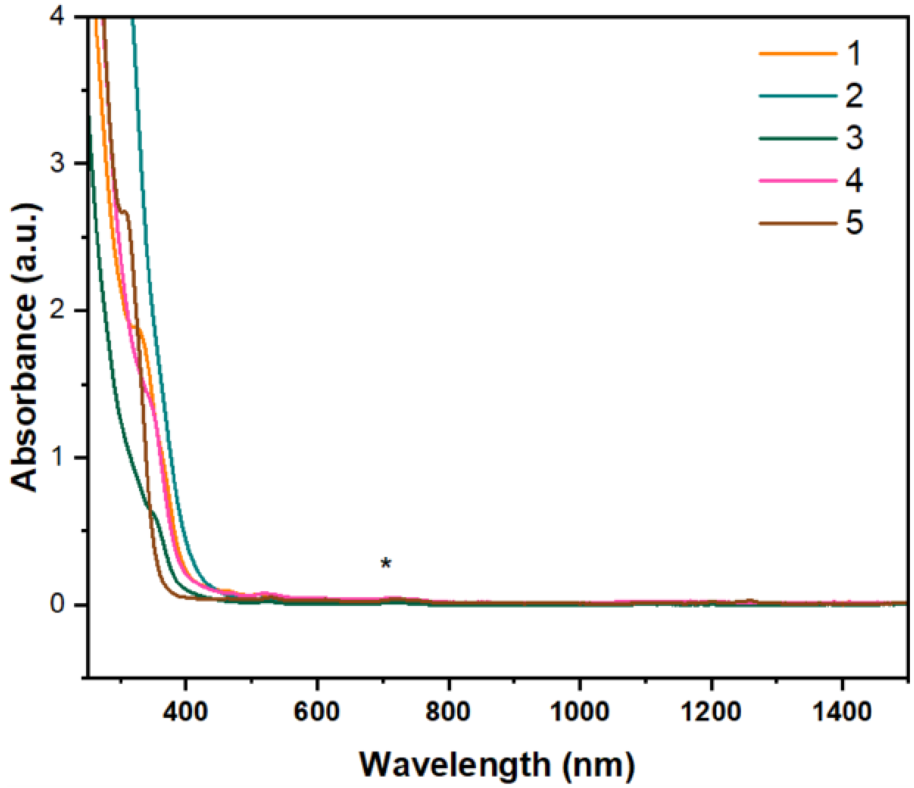

Figure S34. Dilute, co-plotted UV-vis NIR spectra of complexes **1** (350 μM in THF), **2** (350 μM in THF), **3** (200 μM in hexanes), **4** (520 μM in THF), **5** (390 μM in THF).

Infra-Red Spectrum of 7-H and 7-D.

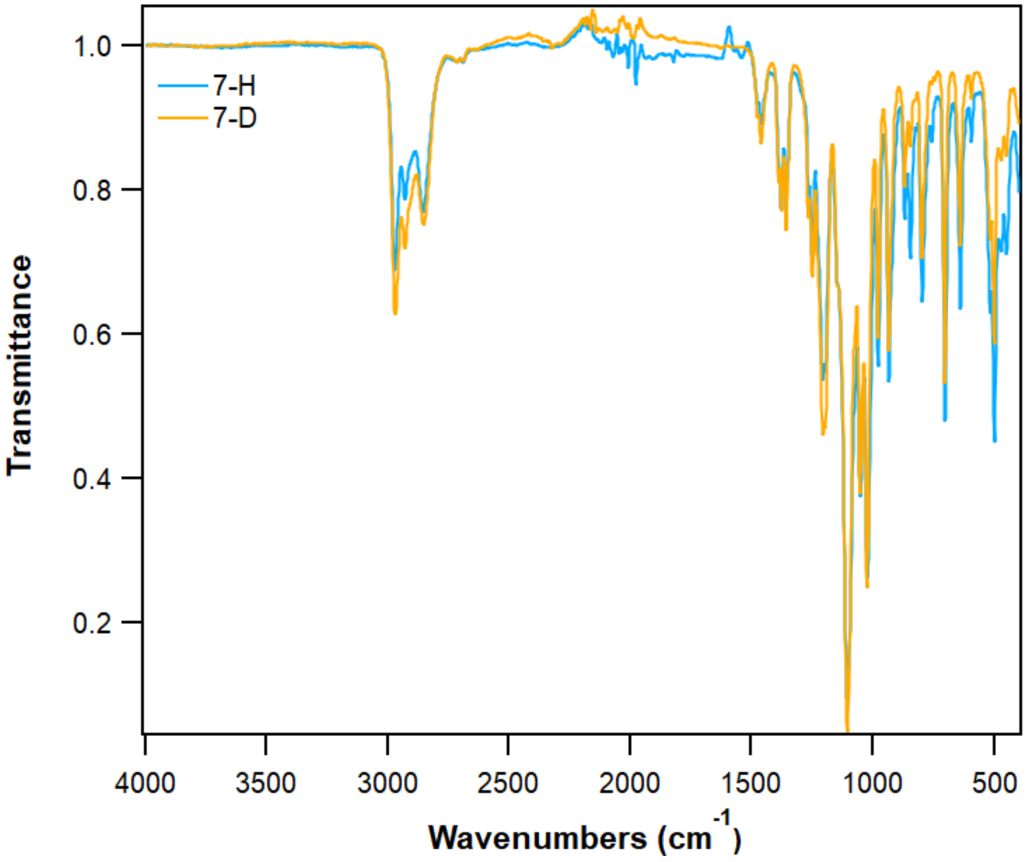

Figure S35. Solution of **7-H** and **7-D** 3 hours after the addition of H_2/D_2 gas show a nearly identical IR spectra, and no clear U-H or U-D shift is identified.

Electrochemistry

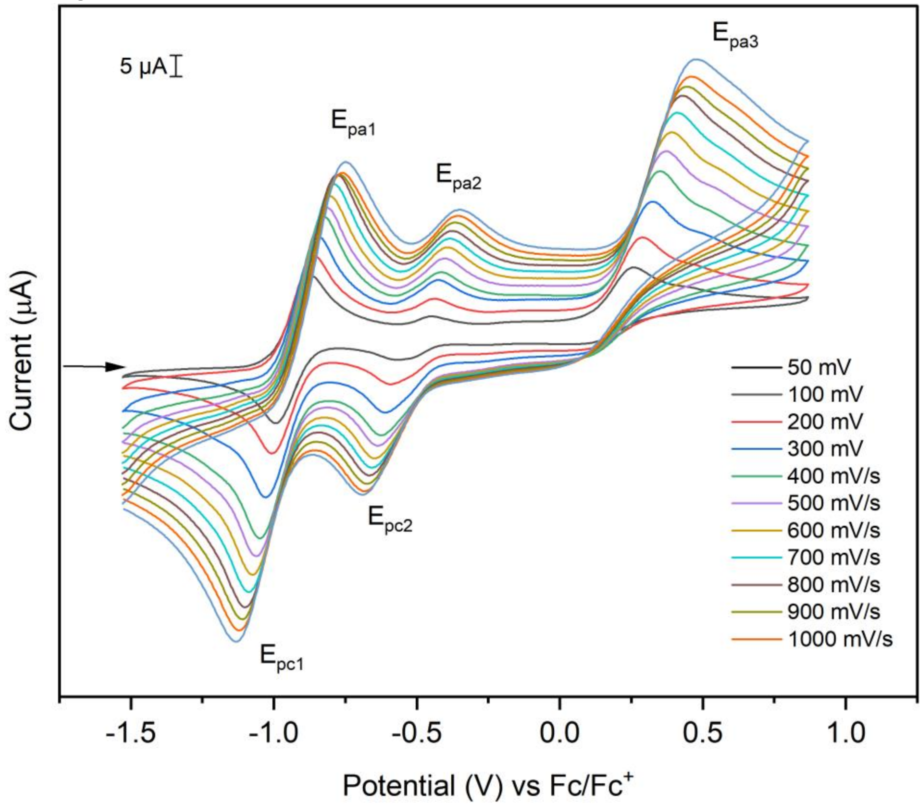

Figure S36. Scan rate dependence of **1** (3 mM) in 50 mM [^{n}Bu$_4$N][PF$_6$] in 1,2-Difluorobenzene.

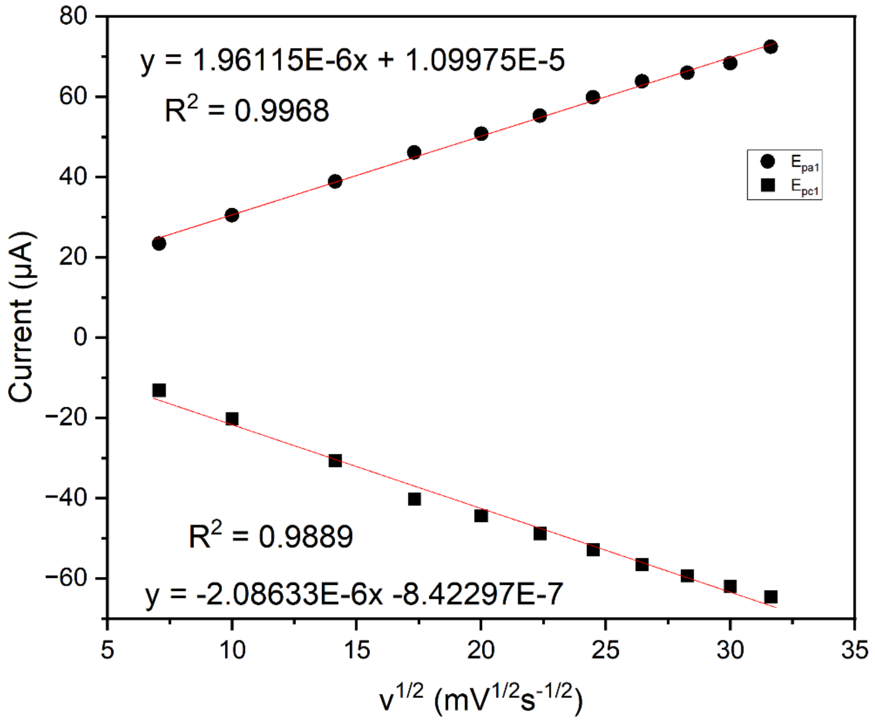

Figure S37. Randles-Sevcik plot of **1** (3 mM) at Epa1 and Epc1 in 50 mM [^{n}Bu$_4$N][PF$_6$] in 1,2-Difluorobenzene.

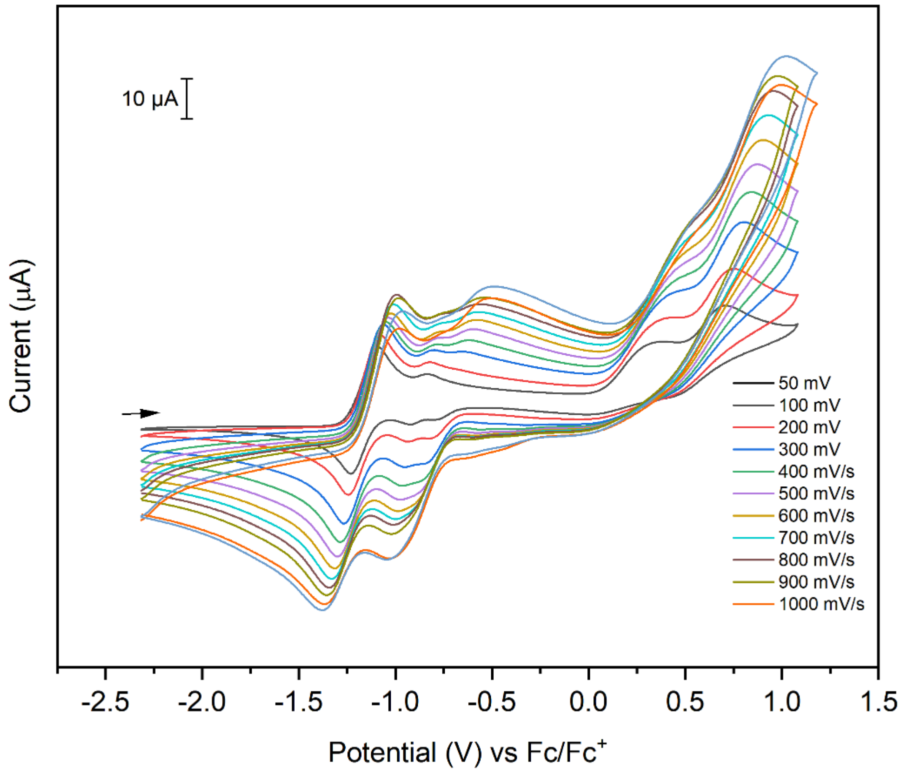

Figure S38. Scan rate dependence of **3** (3 mM) in 50 mM [^{n}Bu$_4$N][BPh$_4$] in 1,2-Difluorobenzene.

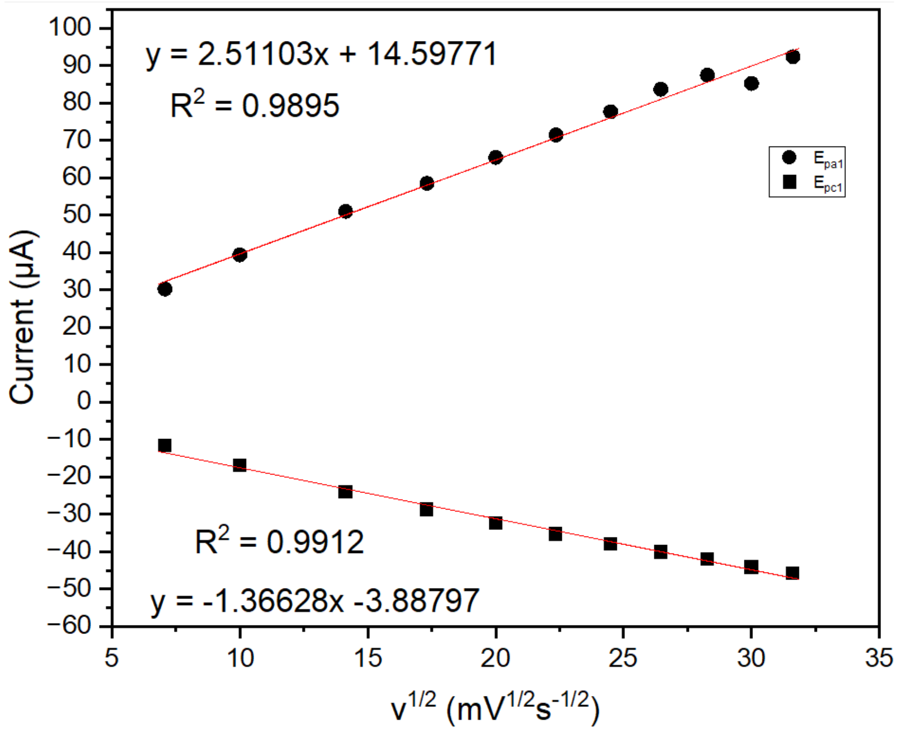

Figure S39. Randles-Sevcik plot of **3** (3 mM) at Epa1 and Epc1 in 50 mM [^{n}Bu$_4$N][PF$_6$] in 1,2-Difluorobenzene.

Crystallographic Analyses

Crystals suitable for X-ray diffraction were covered with Cargille-NVH oil in a N_2 glovebox and transferred to the diffractometer in a capped 20 mL vial. Crystals were cooled to 100K prior to mounting crystals, and kept at 100(2)K throughout the duration of the collection. Data were collected from a shock-cooled single crystal at 100.00 K on a Bruker D8 VENTURE dual wavelength Mo/Cu three-circle diffractometer with a microfocus sealed X-ray tube using a mirror optics as monochromator and a Bruker PHOTON III detector. The diffractometer was equipped with an Oxford Cryostream 800 low temperature device and used MoKα radiation (λ = 0.71073 Å). All data were integrated with SAINT V8.40B and a multi-scan absorption correction using SADABS 2016/2 was applied.[1,2] The structure was solved by direct methods with SHELXT and refined by full-matrix least-squares methods against F2 using XL.[3,4] All non-hydrogen atoms were refined with anisotropic displacement parameters. All hydrogen atoms were refined isotropic on calculated positions using a riding model with their Uiso values constrained to 1.5 times the U_{eq} of their pivot atoms for terminal sp^3 carbon atoms and 1.2 times for all other carbon atoms. Disordered moieties were refined using bond lengths restraints and displacement parameter restraints.

Table S1. Crystallographic data table for Complexes **1-4**.

	**1**	**2**	**3**	**4**
Empirical Formula	$C_{54}H_{124}IN_{12}P_3U$	$C_{48.74}H_{102.75}I_{0.04}N_{12}P_3U$*	$C_{47}H_{107}N_{12}P_3U$	$C_{43}H_{99}N_{12}P_3U$
Formula Weight	1399.48	1192.49	1171.38	1115.28
Temperature/K	100.00	100(2)	100(2)	100.00
Crystal system	monoclinic	triclinic	orthorhombic	monoclinic
Space group	$P2_1/c$	P-1	Pbca (61)	$P2_1/n$
a/Å	10.889(4)	10.5661(14)	19.028(6)	13.1003(10)
b/Å	24.407(10)	13.5294(16)	26.322(8)	19.2482(17)
c/Å	23.258(7)	20.817(3)	46.732(13)	21.8242(17)
α/°	90	90.431(5)	90	90
β/°	91.649(9)	96.263(5)	90	90.376(3)
γ/°	90	97.688(5)	90	90
Volume/Å^3	6178(4)	2930.8(7)	23407(12)	5503.0(8)
Z	4	2	16	4
ρ_{calc}g/cm^3	1.505	1.351	1.330	1.346
μ/mm^{-1}	3.250	2.911	2.896	3.076
F(000)	2888	1236.0	9760.0	2312
Crystal size/mm^3	0.292×0.349×0.512	0.103 × 0.078 × 0.032	0.286×0.326×0.383	0.206×0.198×0.115
Radiation	MoKα (λ = 0.71073)	MoKα (λ = 0.71073)	MoKα (λ = 0.71073)	MoKα (λ=0.71073)
2Θ range for data collection/°	3.74 to 52.80 (0.80 Å)	4.182 to 59.16	3.49 to 56.57 (0.75 Å)	4.21 to 61.02 (0.70 Å)
Index ranges	−13 ≤ h ≤ 13 −30 ≤ k ≤ 30 −25 ≤ l ≤ 29	-14 ≤ h ≤ 14, -18 ≤ k ≤ 18, -28 ≤ l ≤ 28	-−25 ≤ h ≤ 25 −32 ≤ k ≤ 35 −62 ≤ l ≤ 62	−16 ≤ h ≤ 18 −27 ≤ k ≤ 27 −31 ≤ l ≤ 31
Reflections collected	158656	187385	944364	323311
Independent reflections	12639 [R_{int} = 0.0553 R_{sigma} = 0.0235]	16430 [R_{int} = 0.0816, R_{sigma} = 0.0371]	29046 [R_{int} = 0.1468, R_{sigma} = 0.0370]	16794[R_{int} = 0.0665 R_{sigma} = 0.0243]
Data/restraints/parameters	12639 / 0 / 556	16430/3/640	29046 / 767 / 1525	16794/20/618
Goodness-of-fit on F^2	1.024	1.131	1.077	1.099
Final R indexes [I>=2σ (I)]	R_1 = 0.0245 wR_2 = 0.0640	R_1 = 0.0326, wR_2 = 0.0591	R_1 = 0.0443 wR_2 = 0.0925	R_1 = 0.0225 wR_2 = 0.0414

Final R indexes [all data]	R_1 = 0.0256<br>wR_2 = 0.0646	R_1 = 0.0416, wR_2 = 0.0623	R_1 = 0.0612<br>wR_2 = 0.0998	R_1 = 0.0341<br>wR_2 = 0.0462
Largest diff. peak/hole /e Å^{-3}	1.04/−1.04	1.79/-1.40	1.44/−1.09	0.67/-0.80
CCDC Number	2538110	2538367	2538369	2473803

***2** crystal structure includes 1 which co-crystalizes at 0.0368 occupancy.

Table S2. Crystallographic data table for Complexes **5**, **6**, **7-H**.

	**5**	**6**	**7-H**
Empirical Formula	$C_{61}H_{140}N_{16}P_4U$	$C_{78}H_{118}BF_{24}N_{12}OP_3U$	$C_{42}H_{96}I_{0.04}N_{12}P_3U$
Formula Weight	1459.79	2037.59	1104.68
Temperature/K	107.4(2)	100.00	100.00
Crystal system	tetragonal	triclinic	monoclinic
Space group	$I\bar{4}$	P-1	$P2_1/n$ (14)
a/Å	13.3165(7)	13.415(8)	13.143(4)
b/Å	13.3165(7)	17.010(7)	18.804(6)
c/Å	21.3014(18)	20.415(11)	21.805(7)
α/°	90	89.73(2)	90
β/°	90	79.30(4)	90.012(17)
γ/°	90	89.992(16)	90
Volume/Å^3	3777.4(5)	4577(4)	5389(3)
Z	2	2	4
ρ_{calc}g/cm^3	1.283	1.478	1.362
μ/mm^{-1}	2.279	1.923	3.160
F(000)	1540	2072.0	2283
Crystal size/mm^3	0.299×0.19×0.17	0.13 × 0.11 × 0.09	0.069×0.169×0.319
Radiation	MoKα (λ=0.71073)	MoKα (λ = 0.71073)	MoK_α<br>(λ=0.71073 Å)
2Θ range for data collection/°	3.82 to 61.00<br>(0.70 Å)	3.91 to 50.054	4.32 to 52.74<br>(0.80 Å)
Index ranges	−19 ≤ h ≤ 19<br>−19 ≤ k ≤ 19<br>−30 ≤ l ≤ 30	-15 ≤ h ≤ 15<br>-20 ≤ k ≤ 20<br>-24 ≤ l ≤ 24	−15 ≤ h ≤ 16<br>−23 ≤ k ≤ 23<br>−26 ≤ l ≤ 27
Reflections collected	146193	192850	69911
Independent reflections	5752<br>[R_{int} = 0.0812<br>R_{sigma} = 0.0237]	16176<br>[R_{int} = 0.2252, R_{sigma} = 0.0809]	11879 [R_{int} = 0.1476, R_{sigma} = 0.1004]
Data/restraints/parameters	5752/34/230	16176/57/1127	11024 / 287 / 722
Goodness-of-fit on F^2	1.088	1.094	1.037
Final R indexes [I>=2σ (I)]	R_1 = 0.0204<br>wR_2 = 0.0510	R_1 = 0.0736<br>wR_2 = 0.1626	R^1 = 0.0467<br>wR_2 = 0.0862
Final R indexes [all data]	R_1 = 0.0204<br>wR_2 = 0.0510	R_1 = 0.0961<br>wR_2 = 0.1843	R_1 = 0.0829<br>wR_2 = 0.1032
Largest diff. peak/hole /e Å^{-3}	1.18/-0.79	2.23/-3.47	1.35/−1.10
CCDC Number	2473802	2538109	2538368

***7-H** crystal structure includes **1** which co-crystalizes at 0.0356 occupancy.

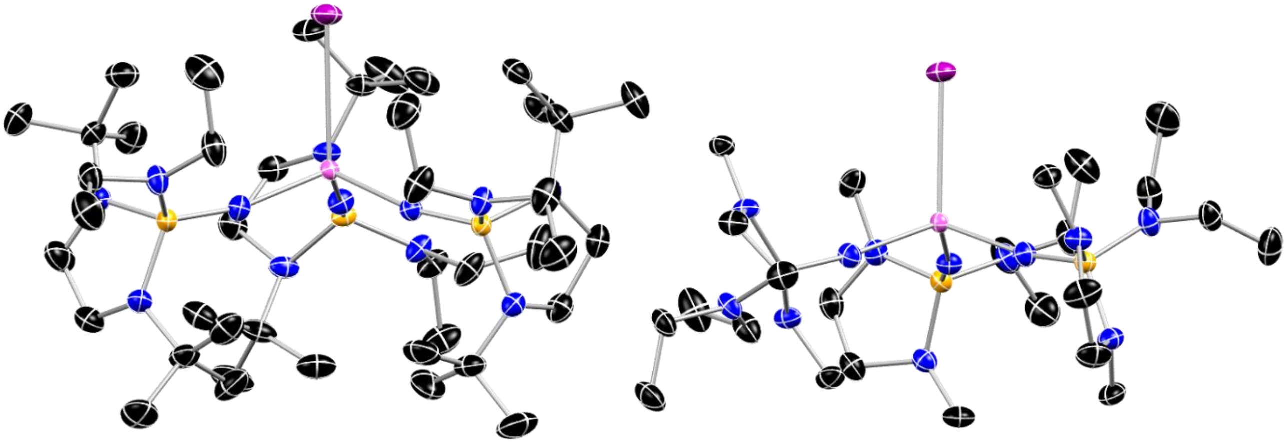

Figure S40. (Left) Asymmetric unit of **1** with thermal ellipsoids shown at 50% probability level. Hydrogen atoms are omitted for clarity. Uranium is shown in pink, carbon is shown in black, nitrogen is shown in blue, phosphorous is shown in orange and iodine is shown in purple. (Right) Truncated asymmetric unit of **1** with thermal ellipsoids shown at 50% probability level. Hydrogen atoms and ^{t}Bu groups on the ligand are omitted for clarity.

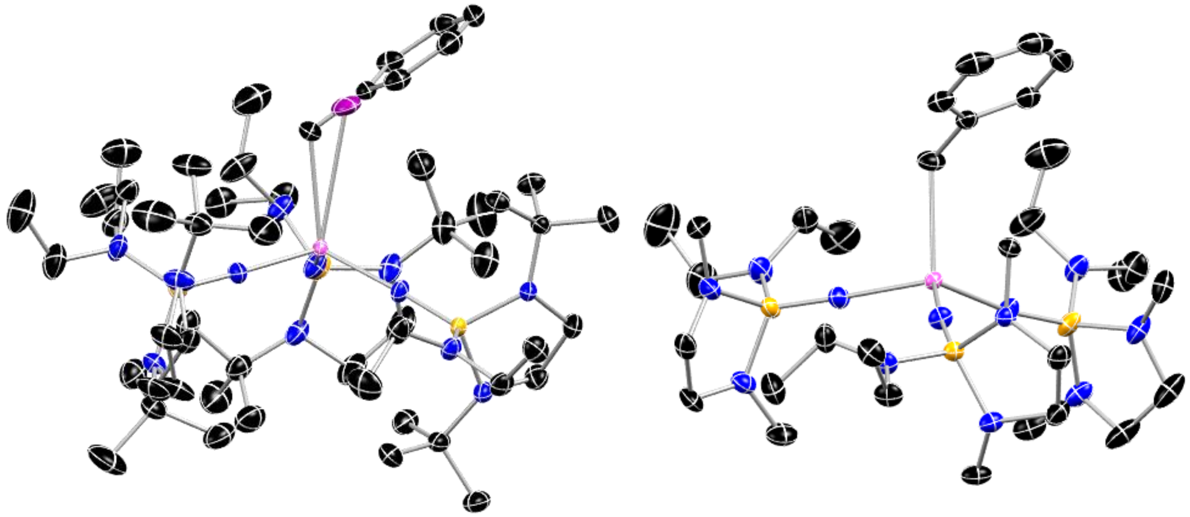

Figure S41. (Left) Asymmetric unit of **2** with thermal ellipsoids shown at 50% probability level. Hydrogen atoms are omitted for clarity. Uranium is shown in pink, carbon is shown in black, nitrogen is shown in blue, and phosphorous is shown in orange. Note that this structure includes **1** which co-crystalizes at 0.0368 occupancy. (Right) Truncated asymmetric unit of **2** with thermal ellipsoids shown at 50% probability level. Hydrogen atoms, ^{t}Bu groups on the ligand, ligand disorder on one of the the ligand (at the N*C*H$_2$-*C*H$_2$N position and NEt$_2$ position),

and co-crystalized 1 are omitted for clarity.

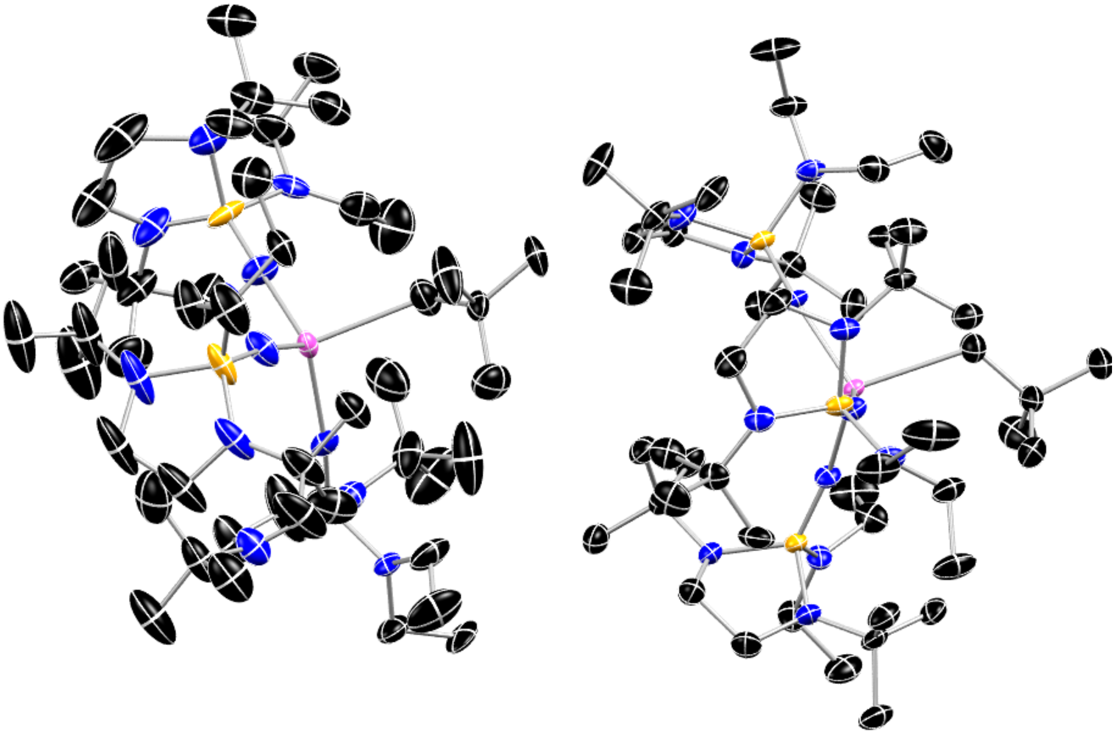

Figure S42. Asymmetric unit of **3** containing two molecules is shown with thermal ellipsoids at 50% probability level. Uranium is shown in pink, carbon is shown in black, nitrogen is shown in blue, and phosphorous is shown in orange. Hydrogen atoms and disorders are omitted for clarity.

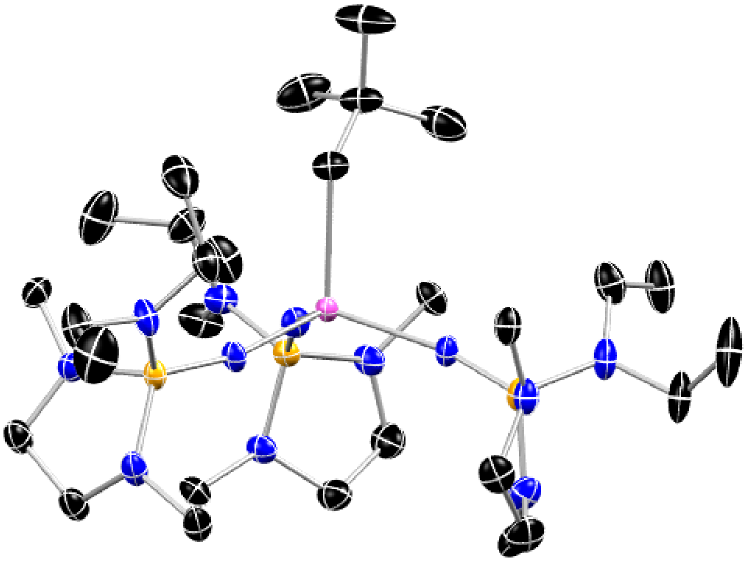

Figure S43. Truncated representation of **3** only showing one molecule, with thermal ellipsoids at 50% probability level. Uranium is shown in pink, carbon is shown in black, nitrogen is shown in blue, and phosphorous is shown in orange. Hydrogen atoms and ^{t}Bu groups on the ligand and another molecule in the asymmetric unit are omitted for clarity.

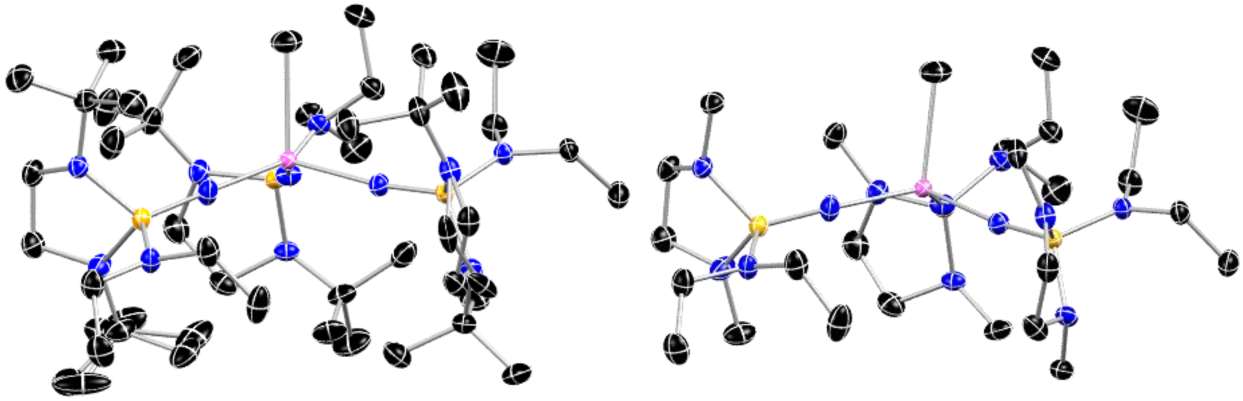

Figure S44. .(Left) Asymmetric unit of **4** with thermal ellipsoids shown at 50% probability level. Hydrogen atoms are omitted for clarity. Uranium is shown in pink, carbon is shown in black, nitrogen is shown in blue, and phosphorous is shown in orange. (Right) Truncated asymmetric unit of **4** with thermal ellipsoids shown at 50% probability level. Hydrogen atoms and ^{t}Bu groups on the ligand are omitted for clarity.

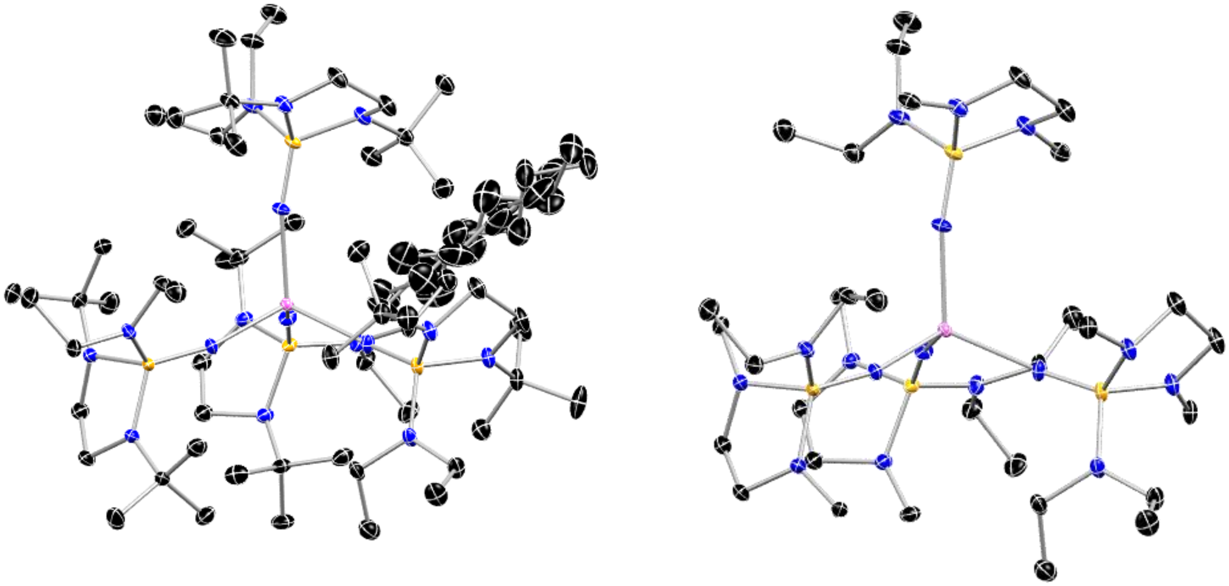

Figure S45. (Left) Asymmetric unit of **5** with thermal ellipsoids shown at 50% probability level. Hydrogen atoms are omitted for clarity. Uranium is shown in pink, carbon is shown in black, nitrogen is shown in blue, and phosphorous is shown in orange. (Right) Truncated asymmetric unit of **5** with thermal ellipsoids shown at 50% probability level. Hydrogen atoms, ^{t}Bu groups on the ligand and pentane are omitted for clarity.

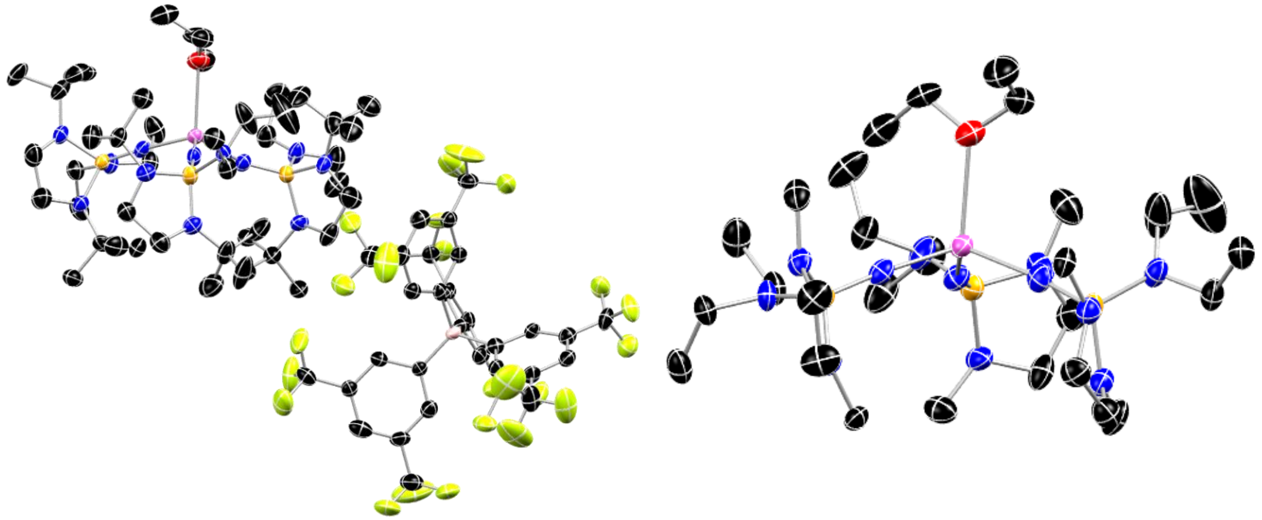

Figure S46. (Left) Asymmetric unit of **6** with thermal ellipsoids shown at 50% probability level. Hydrogen atoms are omitted for clarity. Uranium is shown in pink, carbon is shown in black, nitrogen is shown in blue, phosphorous is shown in orange, boron is shown in light pink, and fluorine is shown in neon. (Right) Truncated asymmetric unit of **6** with thermal ellipsoids shown at 50% probability level. Hydrogen atoms, ^{t}Bu groups on the ligand and $[BArF_{24}]^-$ are omitted for clarity.

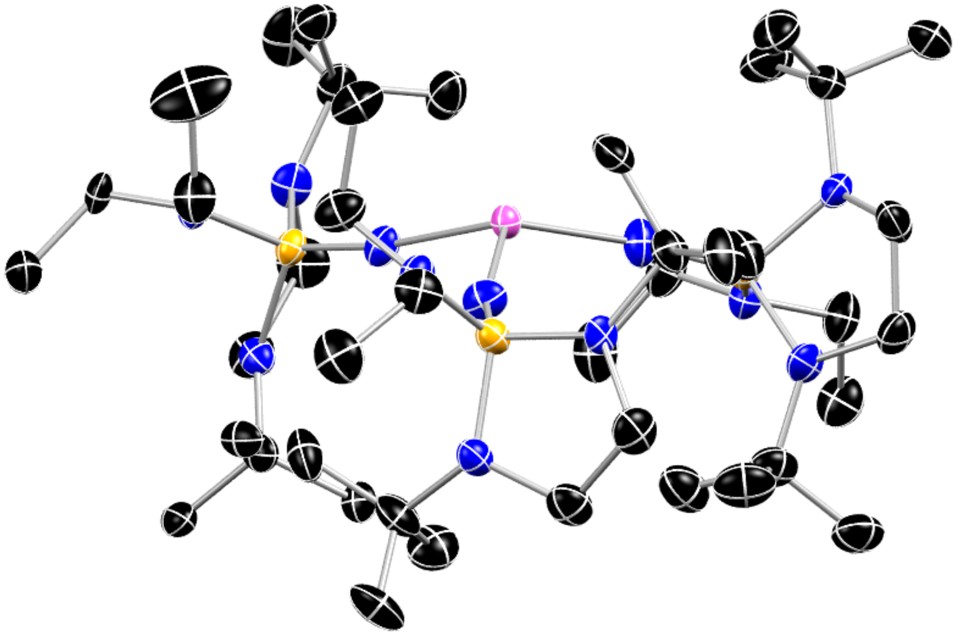

Figure S47. Asymmetric unit of **7-H** with thermal ellipsoids shown at 50% probability level, showing the disordered part which contains 0.0340 occupancy of **1** which co-crystalize. Uranium is shown in pink, carbon is shown in black, nitrogen is shown in blue, and phosphorous is shown in orange.

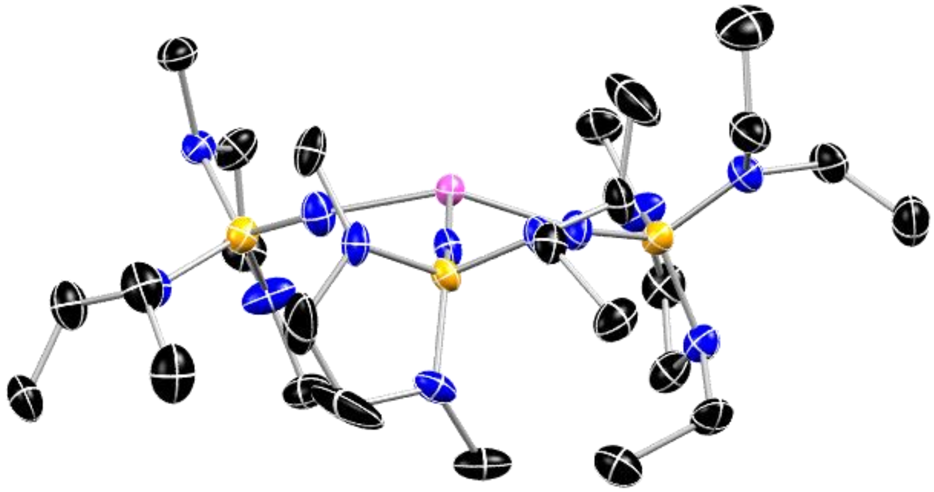

Figure S48. Truncated asymmetric unit of **7-H** shown with thermal ellipsoids at 50% probability level. Uranium is shown in pink, carbon is shown in black, nitrogen is shown in blue, and phosphorous is shown in orange. Hydrogen atoms (excluding U-H atom which cannot be located) ^{t}Bu groups on the ligand, and partial occupation of **1** are omitted for clarity.

Table S3. Bond lengths for **1.**

Atom	Atom	Length/Å	Atom	Atom	Length/Å
U1	I1	3.0602(10)	N11	C35	1.489(4)
U1	N1	2.129(2)	N12	C34	1.471(4)
U1	N2	2.134(2)	N12	C39	1.492(4)
U1	N3	2.134(2)	C1	C2	1.508(5)
P1	N1	1.550(2)	C3	C4	1.526(5)
P1	N4	1.658(3)	C5	C6	1.482(5)
P1	N5	1.674(2)	C7	C8	1.529(5)
P1	N6	1.686(3)	C7	C9	1.529(5)
P2	N2	1.550(2)	C7	C10	1.529(4)
P2	N7	1.656(2)	C11	C12	1.531(5)
P2	N8	1.665(2)	C11	C13	1.528(5)

Atom	Atom	Length/Å	Atom	Atom	Length/Å
P2	N9	1.698(2)	C11	C14	1.529(4)
P3	N3	1.558(2)	C15	C16	1.489(5)
P3	N10	1.658(3)	C17	C18	1.488(5)
P3	N11	1.674(2)	C19	C20	1.511(5)
P3	N12	1.687(2)	C21	C22	1.536(4)
N4	C1	1.469(4)	C21	C23	1.536(4)
N4	C3	1.463(4)	C21	C24	1.521(5)
N5	C5	1.451(4)	C25	C26	1.538(5)
N5	C7	1.483(4)	C25	C27	1.546(5)
N6	C6	1.459(4)	C25	C28	1.531(5)
N6	C11	1.492(4)	C29	C30	1.525(4)
N7	C15	1.474(4)	C31	C32	1.513(5)
N7	C17	1.454(4)	C33	C34	1.506(4)
N8	C19	1.458(4)	C35	C36	1.522(4)
N8	C21	1.484(4)	C35	C37	1.526(4)
N9	C20	1.480(4)	C35	C38	1.519(4)
N9	C25	1.480(4)	C39	C40	1.539(5)
N10	C29	1.469(3)	C39	C41	1.538(4)
N10	C31	1.466(4)	C39	C42	1.530(5)
N11	C33	1.454(3)			

Table S4. Bond angles for **1.**

Atom	Atom	Atom	Angle/˚	Atom	Atom	Atom	Angle/˚
N1	U1	I1	108.18(7)	C34	N12	P3	109.47(18)
N1	U1	N2	105.36(9)	C34	N12	C39	116.5(3)
N1	U1	N3	113.02(9)	C39	N12	P3	123.53(19)
N2	U1	I1	110.18(6)	N4	C1	C2	114.5(3)
N2	U1	N3	109.99(9)	N4	C3	C4	114.2(3)

Atom	Atom	Atom	Angle/˚	Atom	Atom	Atom	Angle/˚
N3	U1	I1	110.01(7)	N5	C5	C6	108.7(3)
N1	P1	N4	110.33(13)	N6	C6	C5	109.2(3)
N1	P1	N5	118.35(13)	N5	C7	C8	110.0(3)
N1	P1	N6	118.67(13)	N5	C7	C9	111.4(2)
N4	P1	N5	106.53(13)	N5	C7	C10	108.6(3)
N4	P1	N6	107.92(13)	C8	C7	C9	109.6(3)
N5	P1	N6	93.36(12)	C8	C7	C10	107.5(3)
N2	P2	N7	110.03(13)	C9	C7	C10	109.5(3)
N2	P2	N8	118.20(13)	N6	C11	C12	111.6(3)
N2	P2	N9	119.17(12)	N6	C11	C13	109.4(3)
N7	P2	N8	107.11(12)	N6	C11	C14	108.5(3)
N7	P2	N9	107.80(12)	C13	C11	C12	109.7(3)
N8	P2	N9	92.90(13)	C13	C11	C14	108.4(3)
N3	P3	N10	110.84(12)	C14	C11	C12	109.2(3)
N3	P3	N11	118.18(12)	N7	C15	C16	115.0(3)
N3	P3	N12	118.68(13)	N7	C17	C18	117.3(3)
N10	P3	N11	107.69(13)	N8	C19	C20	106.1(2)
N10	P3	N12	106.72(13)	N9	C20	C19	105.2(2)
N11	P3	N12	92.93(12)	N8	C21	C22	108.2(3)
P1	N1	U1	167.30(15)	N8	C21	C23	109.8(2)
P2	N2	U1	165.28(15)	N8	C21	C24	110.9(2)
P3	N3	U1	172.58(15)	C23	C21	C22	110.6(3)
C1	N4	P1	120.3(2)	C24	C21	C22	107.9(3)
C3	N4	P1	123.0(2)	C24	C21	C23	109.4(3)
C3	N4	C1	116.6(2)	N9	C25	C26	108.2(3)
C5	N5	P1	113.5(2)	N9	C25	C27	111.7(3)
C5	N5	C7	118.3(2)	N9	C25	C28	109.9(3)

Atom	Atom	Atom	Angle/°	Atom	Atom	Atom	Angle/°
C7	N5	P1	124.64(19)	C26	C25	C27	109.7(3)
C6	N6	P1	111.1(2)	C28	C25	C26	108.0(3)
C6	N6	C11	117.7(3)	C28	C25	C27	109.2(3)
C11	N6	P1	122.6(2)	N10	C29	C30	114.0(3)
C15	N7	P2	120.4(2)	N10	C31	C32	113.3(3)
C17	N7	P2	122.9(2)	N11	C33	C34	105.5(2)
C17	N7	C15	116.6(2)	N12	C34	C33	104.9(2)
C19	N8	P2	113.8(2)	N11	C35	C36	111.2(2)
C19	N8	C21	118.2(2)	N11	C35	C37	109.4(2)
C21	N8	P2	126.7(2)	N11	C35	C38	108.5(3)
C20	N9	P2	107.84(19)	C36	C35	C37	108.4(3)
C25	N9	P2	122.0(2)	C38	C35	C36	108.9(3)
C25	N9	C20	116.7(2)	C38	C35	C37	110.4(3)
C29	N10	P3	119.62(19)	N12	C39	C40	111.4(3)
C31	N10	P3	122.6(2)	N12	C39	C41	107.9(2)
C31	N10	C29	117.5(2)	N12	C39	C42	110.6(3)
C33	N11	P3	113.22(19)	C41	C39	C40	109.3(3)
C33	N11	C35	118.7(2)	C42	C39	C40	108.6(3)
C35	N11	P3	127.96(19)	C42	C39	C41	109.0(3)

Table S5. Bond length for **2.**

Atom–Atom	Length [Å]
U1–N1	2.182(3)
U1–N5	2.179(3)
U1–N9	2.161(3)
U1–C43	2.504(5)
P1–N9	1.539(3)
P1–N10	1.672(4)
P1–N11	1.688(4)
P1–N12	1.683(4)
P2–N1	1.541(4)
P2–N2	1.663(4)
P2–N3	1.690(4)
P2–N4	1.674(4)

P3–N5	1.541(4)
P3–N6	1.662(4)
P3–N7	1.673(4)
P3–N8	1.699(4)
N2–C1	1.455(6)
N2–C3	1.464(6)
N3–C5	1.494(6)
N3–C9	1.459(6)
N4–C10	1.449(6)
N4–C11	1.481(6)
N6–C15	1.456(7)
N6–C17	1.459(6)
N7–C19	1.479(6)
N7–C23	1.446(6)
N8–C24	1.461(6)
N8–C25	1.492(6)
N10–C29	1.461(6)
N10–C31	1.457(6)
N11–C33	1.490(6)
N11–C37	1.463(6)
N12–C38	1.465(6)
N12–C39	1.487(6)
C1–H1A	0.9900
C1–H1B	0.9900
C1–C2	1.491(8)
C2–H2A	0.9800
C2–H2B	0.9800
C2–H2C	0.9800
C3–H3A	0.9900
C3–H3B	0.9900
C3–C4	1.505(7)
C4–H4A	0.9800
C4–H4B	0.9800
C4–H4C	0.9800
C5–C6	1.529(7)
C5–C7	1.524(7)
C5–C8	1.528(6)
C6–H6A	0.9800
C6–H6B	0.9800
C6–H6C	0.9800
C7–H7A	0.9800
C7–H7B	0.9800
C7–H7C	0.9800
C8–H8A	0.9800
C8–H8B	0.9800
C8–H8C	0.9800
C9–H9A	0.9900
C9–H9B	0.9900
C9–C10	1.512(7)
C10–H10A	0.9900
C10–H10B	0.9900
C11–C12	1.535(6)
C11–C13	1.528(7)

C11–C14	1.532(7)
C12–H12A	0.9800
C12–H12B	0.9800
C12–H12C	0.9800
C13–H13A	0.9800
C13–H13B	0.9800
C13–H13C	0.9800
C14–H14A	0.9800
C14–H14B	0.9800
C14–H14C	0.9800
C15–H15A	0.9900
C15–H15B	0.9900
C15–C16	1.527(7)
C16–H16A	0.9800
C16–H16B	0.9800
C16–H16C	0.9800
C17–H17A	0.9900
C17–H17B	0.9900
C17–C18	1.514(9)
C18–H18A	0.9800
C18–H18B	0.9800
C18–H18C	0.9800
C19–C20	1.532(7)
C19–C21	1.528(7)
C19–C22	1.516(7)
C20–H20A	0.9800
C20–H20B	0.9800
C20–H20C	0.9800
C21–H21A	0.9800
C21–H21B	0.9800
C21–H21C	0.9800
C22–H22A	0.9800
C22–H22B	0.9800
C22–H22C	0.9800
C23–H23A	0.9900
C23–H23B	0.9900
C23–C24	1.513(8)
C24–H24A	0.9900
C24–H24B	0.9900
C25–C26	1.538(6)
C25–C27	1.535(6)
C25–C28	1.523(7)
C26–H26A	0.9800
C26–H26B	0.9800
C26–H26C	0.9800
C27–H27A	0.9800
C27–H27B	0.9800
C27–H27C	0.9800
C28–H28A	0.9800
C28–H28B	0.9800
C28–H28C	0.9800
C29–H29A	0.9900
C29–H29B	0.9900

C29–C30	1.510(8)
C30–H30A	0.9800
C30–H30B	0.9800
C30–H30C	0.9800
C31–H31A	0.9900
C31–H31B	0.9900
C31–C32	1.513(8)
C32–H32A	0.9800
C32–H32B	0.9800
C32–H32C	0.9800
C33–C34	1.524(7)
C33–C35	1.526(8)
C33–C36	1.532(7)
C34–H34A	0.9800
C34–H34B	0.9800
C34–H34C	0.9800
C35–H35A	0.9800
C35–H35B	0.9800
C35–H35C	0.9800
C36–H36A	0.9800
C36–H36B	0.9800
C36–H36C	0.9800
C37–H37A	0.9900
C37–H37B	0.9900
C37–C38	1.508(6)
C38–H38A	0.9900
C38–H38B	0.9900
C39–C40	1.523(7)
C39–C41	1.532(6)
C39–C42	1.528(7)
C40–H40A	0.9800
C40–H40B	0.9800
C40–H40C	0.9800
C41–H41A	0.9800
C41–H41B	0.9800
C41–H41C	0.9800
C42–H42A	0.9800
C42–H42B	0.9800
C42–H42C	0.9800
C43–H43A	0.9900
C43–H43B	0.9900
C43–C44	1.539(7)
C44–C45	1.522(7)
C44–C46	1.528(8)
C44–C47	1.525(8)
C45–H45A	0.9800
C45–H45B	0.9800
C45–H45C	0.9800
C46–H46A	0.9800
C46–H46B	0.9800
C46–H46C	0.9800
C47–H47A	0.9800
C47–H47B	0.9800

C47–H47C	0.9800
U2–N13	2.178(4)
U2–N17	2.178(4)
U2–N21	2.169(4)
U2–C90	2.494(11)
U2–C90A	2.498(10)
P4–N13	1.537(4)
P4–N15	1.688(5)
P4–N14	1.687(8)
P4–N16	1.688(11)
P4–N14A	1.674(11)
P4–N16A	1.687(11)
P5–N17	1.544(4)
P5–N18	1.655(5)
P5–N19	1.698(6)
P5–N20	1.678(5)
N15–C52	1.488(8)
N15–C56	1.460(13)
N15–C56A	1.480(11)
N18–C62	1.471(7)
N18–C64	1.468(7)
N19–C66	1.481(9)
N19–C70	1.469(7)
N20–C71	1.452(9)
N20–C72	1.499(10)
N21–P6	1.529(8)
N21–P6A	1.528(7)
C52–C53	1.501(10)
C52–C54	1.532(11)
C52–C55	1.527(9)
C53–H53A	0.9800
C53–H53B	0.9800
C53–H53C	0.9800
C54–H54A	0.9800
C54–H54B	0.9800
C54–H54C	0.9800
C55–H55A	0.9800
C55–H55B	0.9800
C55–H55C	0.9800
C62–H62A	0.9900
C62–H62B	0.9900
C62–C63	1.498(9)
C63–H63A	0.9800
C63–H63B	0.9800
C63–H63C	0.9800
C64–H64A	0.9900
C64–H64B	0.9900
C64–C65	1.511(9)
C65–H65A	0.9800
C65–H65B	0.9800
C65–H65C	0.9800
C66–C67	1.544(8)
C66–C68	1.525(8)

C66–C69	1.518(10)
C67–H67A	0.9800
C67–H67B	0.9800
C67–H67C	0.9800
C68–H68A	0.9800
C68–H68B	0.9800
C68–H68C	0.9800
C69–H69A	0.9800
C69–H69B	0.9800
C69–H69C	0.9800
C70–H70A	0.9900
C70–H70B	0.9900
C70–C71	1.516(13)
C71–H71A	0.9900
C71–H71B	0.9900
C72–C73	1.533(10)
C72–C74	1.535(12)
C72–C75	1.510(13)
C73–H73A	0.9800
C73–H73B	0.9800
C73–H73C	0.9800
C74–H74A	0.9800
C74–H74B	0.9800
C74–H74C	0.9800
C75–H75A	0.9800
C75–H75B	0.9800
C75–H75C	0.9800
P6–N22	1.617(10)
P6–N23	1.699(10)
P6–N24	1.685(11)
N14–C48	1.482(16)
N14–C50	1.433(12)
N16–C57	1.475(17)
N16–C58	1.476(13)
N22–C76	1.487(12)
N22–C78	1.416(13)
N23–C80	1.568(12)
N23–C84	1.424(9)
N24–C85	1.508(13)
N24–C86	1.416(12)
C48–H48A	0.9900
C48–H48B	0.9900
C48–C49	1.49(2)
C49–H49A	0.9800
C49–H49B	0.9800
C49–H49C	0.9800
C50–H50A	0.9900
C50–H50B	0.9900
C50–C51	1.46(2)
C51–H51A	0.9800
C51–H51B	0.9800
C51–H51C	0.9800
C56–H56A	0.9900

C56–H56B	0.9900
C56–C57	1.48(2)
C57–H57A	0.9900
C57–H57B	0.9900
C58–C59	1.527(17)
C58–C60	1.535(15)
C58–C61	1.511(15)
C59–H59A	0.9800
C59–H59B	0.9800
C59–H59C	0.9800
C60–H60A	0.9800
C60–H60B	0.9800
C60–H60C	0.9800
C61–H61A	0.9800
C61–H61B	0.9800
C61–H61C	0.9800
C76–H76A	0.9900
C76–H76B	0.9900
C76–C77	1.517(15)
C77–H77A	0.9800
C77–H77B	0.9800
C77–H77C	0.9800
C78–H78A	0.9900
C78–H78B	0.9900
C78–C79	1.494(15)
C79–H79A	0.9800
C79–H79B	0.9800
C79–H79C	0.9800
C80–C81	1.568(10)
C80–C82	1.564(10)
C80–C83	1.554(9)
C81–H81A	0.9800
C81–H81B	0.9800
C81–H81C	0.9800
C82–H82A	0.9800
C82–H82B	0.9800
C82–H82C	0.9800
C83–H83A	0.9800
C83–H83B	0.9800
C83–H83C	0.9800
C84–H84A	0.9900
C84–H84B	0.9900
C84–C85	1.513(9)
C85–H85A	0.9900
C85–H85B	0.9900
C86–C87	1.493(11)
C86–C88	1.542(11)
C86–C89	1.537(13)
C87–H87A	0.9800
C87–H87B	0.9800
C87–H87C	0.9800
C88–H88A	0.9800
C88–H88B	0.9800

C88–H88C	0.9800
C89–H89A	0.9800
C89–H89B	0.9800
C89–H89C	0.9800
C90–H90A	0.9900
C90–H90B	0.9900
C90–C91	1.508(15)
C91–C92	1.506(12)
C91–C93	1.521(13)
C91–C94	1.519(14)
C92–H92A	0.9800
C92–H92B	0.9800
C92–H92C	0.9800
C93–H93A	0.9800
C93–H93B	0.9800
C93–H93C	0.9800
C94–H94A	0.9800
C94–H94B	0.9800
C94–H94C	0.9800
P6A–N22A	1.70(2)
P6A–N23A	1.724(14)
P6A–N24A	1.666(10)
N14A–C48A	1.44(2)
N14A–C50A	1.444(13)
N16A–C57A	1.484(16)
N16A–C58A	1.462(13)
N22A–C76A	1.517(17)
N22A–C78A	1.499(18)
N23A–C80A	1.34(2)
N23A–C84A	1.405(9)
N24A–C85A	1.468(12)
N24A–C86A	1.427(11)
C48A–H48C	0.9900
C48A–H48D	0.9900
C48A–C49A	1.54(3)
C49A–H49D	0.9800
C49A–H49E	0.9800
C49A–H49F	0.9800
C50A–H50C	0.9900
C50A–H50D	0.9900
C50A–C51A	1.55(2)
C51A–H51D	0.9800
C51A–H51E	0.9800
C51A–H51F	0.9800
C56A–H56C	0.9900
C56A–H56D	0.9900
C56A–C57A	1.53(2)
C57A–H57C	0.9900
C57A–H57D	0.9900
C58A–C59A	1.520(15)
C58A–C60A	1.529(16)
C58A–C61A	1.512(15)
C59A–H59D	0.9800

C59A–H59E	0.9800
C59A–H59F	0.9800
C60A–H60D	0.9800
C60A–H60E	0.9800
C60A–H60F	0.9800
C61A–H61D	0.9800
C61A–H61E	0.9800
C61A–H61F	0.9800
C76A–H76C	0.9900
C76A–H76D	0.9900
C76A–C77A	1.49(2)
C77A–H77D	0.9800
C77A–H77E	0.9800
C77A–H77F	0.9800
C78A–H78C	0.9900
C78A–H78D	0.9900
C78A–C79A	1.45(2)
C79A–H79D	0.9800
C79A–H79E	0.9800
C79A–H79F	0.9800
C80A–C81A	1.592(15)
C80A–C82A	1.555(15)
C80A–C83A	1.546(14)
C81A–H81D	0.9800
C81A–H81E	0.9800
C81A–H81F	0.9800
C82A–H82D	0.9800
C82A–H82E	0.9800
C82A–H82F	0.9800
C83A–H83D	0.9800
C83A–H83E	0.9800
C83A–H83F	0.9800
C84A–H84C	0.9900
C84A–H84D	0.9900
C84A–C85A	1.525(9)
C85A–H85C	0.9900
C85A–H85D	0.9900
C86A–C87A	1.528(12)
C86A–C88A	1.617(12)
C86A–C89A	1.529(12)
C87A–H87D	0.9800
C87A–H87E	0.9800
C87A–H87F	0.9800
C88A–H88D	0.9800
C88A–H88E	0.9800
C88A–H88F	0.9800
C89A–H89D	0.9800
C89A–H89E	0.9800
C89A–H89F	0.9800
C90A–H90C	0.9900
C90A–H90D	0.9900
C90A–C91A	1.505(14)
C91A–C92A	1.501(12)

C91A–C93A	1.525(14)
C91A–C94A	1.516(12)
C92A–H92D	0.9800
C92A–H92E	0.9800
C92A–H92F	0.9800
C93A–H93D	0.9800
C93A–H93E	0.9800
C93A–H93F	0.9800
C94A–H94D	0.9800
C94A–H94E	0.9800
C94A–H94F	0.9800

Table S6. Bond angles for **2.**

**Atom–Atom–Atom**	**Angle [°]**
N1–U1–C43	112.45(15)
N5–U1–N1	113.85(14)
N5–U1–C43	102.68(16)
N9–U1–N1	110.87(14)
N9–U1–N5	114.62(13)
N9–U1–C43	101.41(15)
N9–P1–N10	110.6(2)
N9–P1–N11	119.7(2)
N9–P1–N12	116.93(19)
N10–P1–N11	105.9(2)
N10–P1–N12	109.10(19)
N12–P1–N11	93.01(18)
N1–P2–N2	111.3(2)
N1–P2–N3	120.61(19)
N1–P2–N4	118.1(2)
N2–P2–N3	105.10(19)
N2–P2–N4	107.5(2)
N4–P2–N3	92.07(19)
N5–P3–N6	111.7(2)
N5–P3–N7	118.7(2)
N5–P3–N8	119.3(2)
N6–P3–N7	105.7(2)
N6–P3–N8	106.23(19)
N7–P3–N8	92.8(2)
P2–N1–U1	163.8(2)
C1–N2–P2	122.4(3)
C1–N2–C3	117.1(4)
C3–N2–P2	120.2(3)
C5–N3–P2	123.9(3)
C9–N3–P2	108.8(3)
C9–N3–C5	117.0(4)
C10–N4–P2	114.0(3)
C10–N4–C11	118.2(4)
C11–N4–P2	126.0(3)
P3–N5–U1	163.4(2)
C15–N6–P3	121.7(3)
C15–N6–C17	117.4(4)
C17–N6–P3	120.2(4)

C19–N7–P3	125.9(3)
C23–N7–P3	113.7(3)
C23–N7–C19	119.3(4)
C24–N8–P3	108.2(3)
C24–N8–C25	116.8(4)
C25–N8–P3	122.3(3)
P1–N9–U1	165.3(2)
C29–N10–P1	122.1(3)
C31–N10–P1	120.9(3)
C31–N10–C29	116.8(4)
C33–N11–P1	122.2(3)
C37–N11–P1	110.3(3)
C37–N11–C33	116.6(4)
C38–N12–P1	112.4(3)
C38–N12–C39	118.6(4)
C39–N12–P1	125.8(3)
N2–C1–H1A	108.4
N2–C1–H1B	108.4
N2–C1–C2	115.6(5)
H1A–C1–H1B	107.5
C2–C1–H1A	108.4
C2–C1–H1B	108.4
C1–C2–H2A	109.5
C1–C2–H2B	109.5
C1–C2–H2C	109.5
H2A–C2–H2B	109.5
H2A–C2–H2C	109.5
H2B–C2–H2C	109.5
N2–C3–H3A	108.4
N2–C3–H3B	108.4
N2–C3–C4	115.6(4)
H3A–C3–H3B	107.4
C4–C3–H3A	108.4
C4–C3–H3B	108.4
C3–C4–H4A	109.5
C3–C4–H4B	109.5
C3–C4–H4C	109.5
H4A–C4–H4B	109.5
H4A–C4–H4C	109.5
H4B–C4–H4C	109.5
N3–C5–C6	107.9(4)
N3–C5–C7	110.3(4)
N3–C5–C8	111.4(4)
C7–C5–C6	108.7(4)
C7–C5–C8	108.6(4)
C8–C5–C6	109.9(4)
C5–C6–H6A	109.5
C5–C6–H6B	109.5
C5–C6–H6C	109.5
H6A–C6–H6B	109.5
H6A–C6–H6C	109.5
H6B–C6–H6C	109.5
C5–C7–H7A	109.5

C5–C7–H7B	109.5
C5–C7–H7C	109.5
H7A–C7–H7B	109.5
H7A–C7–H7C	109.5
H7B–C7–H7C	109.5
C5–C8–H8A	109.5
C5–C8–H8B	109.5
C5–C8–H8C	109.5
H8A–C8–H8B	109.5
H8A–C8–H8C	109.5
H8B–C8–H8C	109.5
N3–C9–H9A	110.6
N3–C9–H9B	110.6
N3–C9–C10	105.6(4)
H9A–C9–H9B	108.8
C10–C9–H9A	110.6
C10–C9–H9B	110.6
N4–C10–C9	105.9(4)
N4–C10–H10A	110.6
N4–C10–H10B	110.6
C9–C10–H10A	110.6
C9–C10–H10B	110.6
H10A–C10–H10B	108.7
N4–C11–C12	108.6(4)
N4–C11–C13	110.5(4)
N4–C11–C14	110.5(4)
C13–C11–C12	110.1(4)
C13–C11–C14	108.9(4)
C14–C11–C12	108.3(4)
C11–C12–H12A	109.5
C11–C12–H12B	109.5
C11–C12–H12C	109.5
H12A–C12–H12B	109.5
H12A–C12–H12C	109.5
H12B–C12–H12C	109.5
C11–C13–H13A	109.5
C11–C13–H13B	109.5
C11–C13–H13C	109.5
H13A–C13–H13B	109.5
H13A–C13–H13C	109.5
H13B–C13–H13C	109.5
C11–C14–H14A	109.5
C11–C14–H14B	109.5
C11–C14–H14C	109.5
H14A–C14–H14B	109.5
H14A–C14–H14C	109.5
H14B–C14–H14C	109.5
N6–C15–H15A	108.7
N6–C15–H15B	108.7
N6–C15–C16	114.3(5)
H15A–C15–H15B	107.6
C16–C15–H15A	108.7
C16–C15–H15B	108.7

C15–C16–H16A	109.5
C15–C16–H16B	109.5
C15–C16–H16C	109.5
H16A–C16–H16B	109.5
H16A–C16–H16C	109.5
H16B–C16–H16C	109.5
N6–C17–H17A	108.5
N6–C17–H17B	108.5
N6–C17–C18	115.2(5)
H17A–C17–H17B	107.5
C18–C17–H17A	108.5
C18–C17–H17B	108.5
C17–C18–H18A	109.5
C17–C18–H18B	109.5
C17–C18–H18C	109.5
H18A–C18–H18B	109.5
H18A–C18–H18C	109.5
H18B–C18–H18C	109.5
N7–C19–C20	108.9(4)
N7–C19–C21	110.9(4)
N7–C19–C22	110.2(4)
C21–C19–C20	107.7(4)
C22–C19–C20	109.9(5)
C22–C19–C21	109.3(4)
C19–C20–H20A	109.5
C19–C20–H20B	109.5
C19–C20–H20C	109.5
H20A–C20–H20B	109.5
H20A–C20–H20C	109.5
H20B–C20–H20C	109.5
C19–C21–H21A	109.5
C19–C21–H21B	109.5
C19–C21–H21C	109.5
H21A–C21–H21B	109.5
H21A–C21–H21C	109.5
H21B–C21–H21C	109.5
C19–C22–H22A	109.5
C19–C22–H22B	109.5
C19–C22–H22C	109.5
H22A–C22–H22B	109.5
H22A–C22–H22C	109.5
H22B–C22–H22C	109.5
N7–C23–H23A	110.6
N7–C23–H23B	110.6
N7–C23–C24	105.7(4)
H23A–C23–H23B	108.7
C24–C23–H23A	110.6
C24–C23–H23B	110.6
N8–C24–C23	105.7(4)
N8–C24–H24A	110.6
N8–C24–H24B	110.6
C23–C24–H24A	110.6
C23–C24–H24B	110.6

H24A–C24–H24B	108.7
N8–C25–C26	108.5(4)
N8–C25–C27	111.7(4)
N8–C25–C28	110.2(4)
C27–C25–C26	109.3(4)
C28–C25–C26	108.6(4)
C28–C25–C27	108.5(4)
C25–C26–H26A	109.5
C25–C26–H26B	109.5
C25–C26–H26C	109.5
H26A–C26–H26B	109.5
H26A–C26–H26C	109.5
H26B–C26–H26C	109.5
C25–C27–H27A	109.5
C25–C27–H27B	109.5
C25–C27–H27C	109.5
H27A–C27–H27B	109.5
H27A–C27–H27C	109.5
H27B–C27–H27C	109.5
C25–C28–H28A	109.5
C25–C28–H28B	109.5
C25–C28–H28C	109.5
H28A–C28–H28B	109.5
H28A–C28–H28C	109.5
H28B–C28–H28C	109.5
N10–C29–H29A	108.7
N10–C29–H29B	108.7
N10–C29–C30	114.4(4)
H29A–C29–H29B	107.6
C30–C29–H29A	108.7
C30–C29–H29B	108.7
C29–C30–H30A	109.5
C29–C30–H30B	109.5
C29–C30–H30C	109.5
H30A–C30–H30B	109.5
H30A–C30–H30C	109.5
H30B–C30–H30C	109.5
N10–C31–H31A	108.4
N10–C31–H31B	108.4
N10–C31–C32	115.3(5)
H31A–C31–H31B	107.5
C32–C31–H31A	108.4
C32–C31–H31B	108.4
C31–C32–H32A	109.5
C31–C32–H32B	109.5
C31–C32–H32C	109.5
H32A–C32–H32B	109.5
H32A–C32–H32C	109.5
H32B–C32–H32C	109.5
N11–C33–C34	110.3(4)
N11–C33–C35	111.2(4)
N11–C33–C36	108.6(4)
C34–C33–C35	108.4(5)

C34–C33–C36	107.8(4)
C35–C33–C36	110.4(5)
C33–C34–H34A	109.5
C33–C34–H34B	109.5
C33–C34–H34C	109.5
H34A–C34–H34B	109.5
H34A–C34–H34C	109.5
H34B–C34–H34C	109.5
C33–C35–H35A	109.5
C33–C35–H35B	109.5
C33–C35–H35C	109.5
H35A–C35–H35B	109.5
H35A–C35–H35C	109.5
H35B–C35–H35C	109.5
C33–C36–H36A	109.5
C33–C36–H36B	109.5
C33–C36–H36C	109.5
H36A–C36–H36B	109.5
H36A–C36–H36C	109.5
H36B–C36–H36C	109.5
N11–C37–H37A	110.7
N11–C37–H37B	110.7
N11–C37–C38	105.2(4)
H37A–C37–H37B	108.8
C38–C37–H37A	110.7
C38–C37–H37B	110.7
N12–C38–C37	104.5(4)
N12–C38–H38A	110.9
N12–C38–H38B	110.9
C37–C38–H38A	110.9
C37–C38–H38B	110.9
H38A–C38–H38B	108.9
N12–C39–C40	112.2(4)
N12–C39–C41	109.5(4)
N12–C39–C42	109.3(4)
C40–C39–C41	108.4(4)
C40–C39–C42	109.6(4)
C42–C39–C41	107.8(4)
C39–C40–H40A	109.5
C39–C40–H40B	109.5
C39–C40–H40C	109.5
H40A–C40–H40B	109.5
H40A–C40–H40C	109.5
H40B–C40–H40C	109.5
C39–C41–H41A	109.5
C39–C41–H41B	109.5
C39–C41–H41C	109.5
H41A–C41–H41B	109.5
H41A–C41–H41C	109.5
H41B–C41–H41C	109.5
C39–C42–H42A	109.5
C39–C42–H42B	109.5
C39–C42–H42C	109.5

H42A–C42–H42B	109.5
H42A–C42–H42C	109.5
H42B–C42–H42C	109.5
U1–C43–H43A	107.1
U1–C43–H43B	107.1
H43A–C43–H43B	106.8
C44–C43–U1	120.9(3)
C44–C43–H43A	107.1
C44–C43–H43B	107.1
C45–C44–C43	111.9(4)
C45–C44–C46	108.1(5)
C45–C44–C47	110.1(5)
C46–C44–C43	108.2(5)
C47–C44–C43	109.4(4)
C47–C44–C46	109.0(5)
C44–C45–H45A	109.5
C44–C45–H45B	109.5
C44–C45–H45C	109.5
H45A–C45–H45B	109.5
H45A–C45–H45C	109.5
H45B–C45–H45C	109.5
C44–C46–H46A	109.5
C44–C46–H46B	109.5
C44–C46–H46C	109.5
H46A–C46–H46B	109.5
H46A–C46–H46C	109.5
H46B–C46–H46C	109.5
C44–C47–H47A	109.5
C44–C47–H47B	109.5
C44–C47–H47C	109.5
H47A–C47–H47B	109.5
H47A–C47–H47C	109.5
H47B–C47–H47C	109.5
N13–U2–N17	114.99(17)
N13–U2–C90	99.3(5)
N13–U2–C90A	114.6(3)
N17–U2–C90	115.6(4)
N17–U2–C90A	98.6(4)
N21–U2–N13	114.14(16)
N21–U2–N17	113.56(18)
N21–U2–C90	97.1(4)
N21–U2–C90A	98.8(3)
N13–P4–N15	119.4(2)
N13–P4–N14	114.8(4)
N13–P4–N16	113.7(7)
N13–P4–N14A	105.9(5)
N13–P4–N16A	123.3(7)
N15–P4–N16	100.0(5)
N14–P4–N15	98.7(4)
N14–P4–N16	108.2(9)
N14A–P4–N15	115.1(4)
N14A–P4–N16A	107.8(9)
N16A–P4–N15	84.4(4)

N17–P5–N18	111.9(2)
N17–P5–N19	119.9(3)
N17–P5–N20	118.9(3)
N18–P5–N19	105.4(3)
N18–P5–N20	105.7(3)
N20–P5–N19	92.5(3)
P4–N13–U2	167.5(3)
C52–N15–P4	123.7(4)
C56–N15–P4	105.3(9)
C56–N15–C52	125.1(10)
C56A–N15–P4	116.2(7)
C56A–N15–C52	109.5(7)
P5–N17–U2	167.9(3)
C62–N18–P5	120.4(4)
C64–N18–P5	122.8(4)
C64–N18–C62	116.9(5)
C66–N19–P5	122.3(4)
C70–N19–P5	108.8(4)
C70–N19–C66	116.4(6)
C71–N20–P5	112.9(5)
C71–N20–C72	119.9(5)
C72–N20–P5	125.8(4)
P6–N21–U2	168.6(4)
P6A–N21–U2	175.6(4)
N15–C52–C53	111.7(6)
N15–C52–C54	110.9(5)
N15–C52–C55	111.3(7)
C53–C52–C54	107.3(8)
C53–C52–C55	109.5(6)
C55–C52–C54	105.9(7)
C52–C53–H53A	109.5
C52–C53–H53B	109.5
C52–C53–H53C	109.5
H53A–C53–H53B	109.5
H53A–C53–H53C	109.5
H53B–C53–H53C	109.5
C52–C54–H54A	109.5
C52–C54–H54B	109.5
C52–C54–H54C	109.5
H54A–C54–H54B	109.5
H54A–C54–H54C	109.5
H54B–C54–H54C	109.5
C52–C55–H55A	109.5
C52–C55–H55B	109.5
C52–C55–H55C	109.5
H55A–C55–H55B	109.5
H55A–C55–H55C	109.5
H55B–C55–H55C	109.5
N18–C62–H62A	108.5
N18–C62–H62B	108.5
N18–C62–C63	115.0(5)
H62A–C62–H62B	107.5
C63–C62–H62A	108.5

C63–C62–H62B	108.5
C62–C63–H63A	109.5
C62–C63–H63B	109.5
C62–C63–H63C	109.5
H63A–C63–H63B	109.5
H63A–C63–H63C	109.5
H63B–C63–H63C	109.5
N18–C64–H64A	108.6
N18–C64–H64B	108.6
N18–C64–C65	114.7(5)
H64A–C64–H64B	107.6
C65–C64–H64A	108.6
C65–C64–H64B	108.6
C64–C65–H65A	109.5
C64–C65–H65B	109.5
C64–C65–H65C	109.5
H65A–C65–H65B	109.5
H65A–C65–H65C	109.5
H65B–C65–H65C	109.5
N19–C66–C67	108.4(6)
N19–C66–C68	110.2(6)
N19–C66–C69	112.6(5)
C68–C66–C67	107.2(5)
C69–C66–C67	109.4(7)
C69–C66–C68	108.9(6)
C66–C67–H67A	109.5
C66–C67–H67B	109.5
C66–C67–H67C	109.5
H67A–C67–H67B	109.5
H67A–C67–H67C	109.5
H67B–C67–H67C	109.5
C66–C68–H68A	109.5
C66–C68–H68B	109.5
C66–C68–H68C	109.5
H68A–C68–H68B	109.5
H68A–C68–H68C	109.5
H68B–C68–H68C	109.5
C66–C69–H69A	109.5
C66–C69–H69B	109.5
C66–C69–H69C	109.5
H69A–C69–H69B	109.5
H69A–C69–H69C	109.5
H69B–C69–H69C	109.5
N19–C70–H70A	110.9
N19–C70–H70B	110.9
N19–C70–C71	104.3(7)
H70A–C70–H70B	108.9
C71–C70–H70A	110.9
C71–C70–H70B	110.9
N20–C71–C70	107.2(5)
N20–C71–H71A	110.3
N20–C71–H71B	110.3
C70–C71–H71A	110.3

C70–C71–H71B	110.3
H71A–C71–H71B	108.5
N20–C72–C73	108.1(6)
N20–C72–C74	108.2(9)
N20–C72–C75	110.7(6)
C73–C72–C74	109.3(6)
C75–C72–C73	110.4(9)
C75–C72–C74	110.1(7)
C72–C73–H73A	109.5
C72–C73–H73B	109.5
C72–C73–H73C	109.5
H73A–C73–H73B	109.5
H73A–C73–H73C	109.5
H73B–C73–H73C	109.5
C72–C74–H74A	109.5
C72–C74–H74B	109.5
C72–C74–H74C	109.5
H74A–C74–H74B	109.5
H74A–C74–H74C	109.5
H74B–C74–H74C	109.5
C72–C75–H75A	109.5
C72–C75–H75B	109.5
C72–C75–H75C	109.5
H75A–C75–H75B	109.5
H75A–C75–H75C	109.5
H75B–C75–H75C	109.5
N21–P6–N22	119.3(6)
N21–P6–N23	118.3(6)
N21–P6–N24	116.0(7)
N22–P6–N23	107.5(6)
N22–P6–N24	96.1(8)
N24–P6–N23	95.0(6)
C48–N14–P4	120.0(10)
C50–N14–P4	121.0(9)
C50–N14–C48	118.8(10)
C57–N16–P4	107.2(10)
C57–N16–C58	117.0(12)
C58–N16–P4	130.0(10)
C76–N22–P6	120.9(9)
C78–N22–P6	122.0(8)
C78–N22–C76	116.3(9)
C80–N23–P6	120.7(6)
C84–N23–P6	116.9(10)
C84–N23–C80	119.5(9)
C85–N24–P6	101.2(10)
C86–N24–P6	135.9(10)
C86–N24–C85	111.6(10)
N14–C48–H48A	109.4
N14–C48–H48B	109.4
N14–C48–C49	111.3(13)
H48A–C48–H48B	108.0
C49–C48–H48A	109.4
C49–C48–H48B	109.4

C48–C49–H49A	109.5
C48–C49–H49B	109.5
C48–C49–H49C	109.5
H49A–C49–H49B	109.5
H49A–C49–H49C	109.5
H49B–C49–H49C	109.5
N14–C50–H50A	108.0
N14–C50–H50B	108.0
N14–C50–C51	117.2(14)
H50A–C50–H50B	107.2
C51–C50–H50A	108.0
C51–C50–H50B	108.0
C50–C51–H51A	109.5
C50–C51–H51B	109.5
C50–C51–H51C	109.5
H51A–C51–H51B	109.5
H51A–C51–H51C	109.5
H51B–C51–H51C	109.5
N15–C56–H56A	108.7
N15–C56–H56B	108.7
N15–C56–C57	114.1(14)
H56A–C56–H56B	107.6
C57–C56–H56A	108.7
C57–C56–H56B	108.7
N16–C57–C56	110.3(12)
N16–C57–H57A	109.6
N16–C57–H57B	109.6
C56–C57–H57A	109.6
C56–C57–H57B	109.6
H57A–C57–H57B	108.1
N16–C58–C59	112.0(13)
N16–C58–C60	109.8(12)
N16–C58–C61	111.2(13)
C59–C58–C60	107.1(14)
C61–C58–C59	107.4(12)
C61–C58–C60	109.1(12)
C58–C59–H59A	109.5
C58–C59–H59B	109.5
C58–C59–H59C	109.5
H59A–C59–H59B	109.5
H59A–C59–H59C	109.5
H59B–C59–H59C	109.5
C58–C60–H60A	109.5
C58–C60–H60B	109.5
C58–C60–H60C	109.5
H60A–C60–H60B	109.5
H60A–C60–H60C	109.5
H60B–C60–H60C	109.5
C58–C61–H61A	109.5
C58–C61–H61B	109.5
C58–C61–H61C	109.5
H61A–C61–H61B	109.5
H61A–C61–H61C	109.5

H61B–C61–H61C	109.5
N22–C76–H76A	108.7
N22–C76–H76B	108.7
N22–C76–C77	114.3(10)
H76A–C76–H76B	107.6
C77–C76–H76A	108.7
C77–C76–H76B	108.7
C76–C77–H77A	109.5
C76–C77–H77B	109.5
C76–C77–H77C	109.5
H77A–C77–H77B	109.5
H77A–C77–H77C	109.5
H77B–C77–H77C	109.5
N22–C78–H78A	107.2
N22–C78–H78B	107.2
N22–C78–C79	120.5(10)
H78A–C78–H78B	106.8
C79–C78–H78A	107.2
C79–C78–H78B	107.2
C78–C79–H79A	109.5
C78–C79–H79B	109.5
C78–C79–H79C	109.5
H79A–C79–H79B	109.5
H79A–C79–H79C	109.5
H79B–C79–H79C	109.5
N23–C80–C81	106.7(8)
C82–C80–N23	109.9(7)
C82–C80–C81	110.1(7)
C83–C80–N23	105.7(7)
C83–C80–C81	110.4(7)
C83–C80–C82	113.7(7)
C80–C81–H81A	109.5
C80–C81–H81B	109.5
C80–C81–H81C	109.5
H81A–C81–H81B	109.5
H81A–C81–H81C	109.5
H81B–C81–H81C	109.5
C80–C82–H82A	109.5
C80–C82–H82B	109.5
C80–C82–H82C	109.5
H82A–C82–H82B	109.5
H82A–C82–H82C	109.5
H82B–C82–H82C	109.5
C80–C83–H83A	109.5
C80–C83–H83B	109.5
C80–C83–H83C	109.5
H83A–C83–H83B	109.5
H83A–C83–H83C	109.5
H83B–C83–H83C	109.5
N23–C84–H84A	112.8
N23–C84–H84B	112.8
N23–C84–C85	94.5(12)
H84A–C84–H84B	110.3

C85–C84–H84A	112.8
C85–C84–H84B	112.8
N24–C85–C84	115.7(15)
N24–C85–H85A	108.4
N24–C85–H85B	108.4
C84–C85–H85A	108.4
C84–C85–H85B	108.4
H85A–C85–H85B	107.4
N24–C86–C87	118.3(12)
N24–C86–C88	97.1(11)
N24–C86–C89	109.4(11)
C87–C86–C88	109.9(12)
C87–C86–C89	112.0(11)
C89–C86–C88	109.1(11)
C86–C87–H87A	109.5
C86–C87–H87B	109.5
C86–C87–H87C	109.5
H87A–C87–H87B	109.5
H87A–C87–H87C	109.5
H87B–C87–H87C	109.5
C86–C88–H88A	109.5
C86–C88–H88B	109.5
C86–C88–H88C	109.5
H88A–C88–H88B	109.5
H88A–C88–H88C	109.5
H88B–C88–H88C	109.5
C86–C89–H89A	109.5
C86–C89–H89B	109.5
C86–C89–H89C	109.5
H89A–C89–H89B	109.5
H89A–C89–H89C	109.5
H89B–C89–H89C	109.5
U2–C90–H90A	106.3
U2–C90–H90B	106.3
H90A–C90–H90B	106.4
C91–C90–U2	124.1(11)
C91–C90–H90A	106.3
C91–C90–H90B	106.3
C90–C91–C93	106.9(13)
C90–C91–C94	107.1(14)
C92–C91–C90	120.3(17)
C92–C91–C93	119.7(16)
C92–C91–C94	89.1(12)
C93–C91–C94	111.6(15)
C91–C92–H92A	109.5
C91–C92–H92B	109.5
C91–C92–H92C	109.5
H92A–C92–H92B	109.5
H92A–C92–H92C	109.5
H92B–C92–H92C	109.5
C91–C93–H93A	109.5
C91–C93–H93B	109.5
C91–C93–H93C	109.5

H93A–C93–H93B	109.5
H93A–C93–H93C	109.5
H93B–C93–H93C	109.5
C91–C94–H94A	109.5
C91–C94–H94B	109.5
C91–C94–H94C	109.5
H94A–C94–H94B	109.5
H94A–C94–H94C	109.5
H94B–C94–H94C	109.5
N21–P6A–N22A	109.8(8)
N21–P6A–N23A	105.0(8)
N21–P6A–N24A	121.9(6)
N22A–P6A–N23A	115.7(12)
N24A–P6A–N22A	113.3(8)
N24A–P6A–N23A	89.5(7)
C48A–N14A–P4	116.6(13)
C48A–N14A–C50A	117.0(14)
C50A–N14A–P4	125.9(11)
C57A–N16A–P4	119.4(9)
C58A–N16A–P4	122.5(9)
C58A–N16A–C57A	118.0(11)
C76A–N22A–P6A	113.5(14)
C78A–N22A–P6A	136.1(13)
C78A–N22A–C76A	109.5(14)
C80A–N23A–P6A	133.7(15)
C80A–N23A–C84A	106.7(13)
C84A–N23A–P6A	114.1(12)
C85A–N24A–P6A	110.6(8)
C86A–N24A–P6A	123.2(8)
C86A–N24A–C85A	113.6(9)
N14A–C48A–H48C	109.5
N14A–C48A–H48D	109.5
N14A–C48A–C49A	110.7(14)
H48C–C48A–H48D	108.1
C49A–C48A–H48C	109.5
C49A–C48A–H48D	109.5
C48A–C49A–H49D	109.5
C48A–C49A–H49E	109.5
C48A–C49A–H49F	109.5
H49D–C49A–H49E	109.5
H49D–C49A–H49F	109.5
H49E–C49A–H49F	109.5
N14A–C50A–H50C	108.0
N14A–C50A–H50D	108.0
N14A–C50A–C51A	117.3(13)
H50C–C50A–H50D	107.2
C51A–C50A–H50C	108.0
C51A–C50A–H50D	108.0
C50A–C51A–H51D	109.5
C50A–C51A–H51E	109.5
C50A–C51A–H51F	109.5
H51D–C51A–H51E	109.5
H51D–C51A–H51F	109.5

H51E–C51A–H51F	109.5
N15–C56A–H56C	112.1
N15–C56A–H56D	112.1
N15–C56A–C57A	98.5(10)
H56C–C56A–H56D	109.7
C57A–C56A–H56C	112.1
C57A–C56A–H56D	112.1
N16A–C57A–C56A	101.6(13)
N16A–C57A–H57C	111.5
N16A–C57A–H57D	111.5
C56A–C57A–H57C	111.5
C56A–C57A–H57D	111.5
H57C–C57A–H57D	109.3
N16A–C58A–C59A	109.0(12)
N16A–C58A–C60A	110.4(12)
N16A–C58A–C61A	112.1(13)
C59A–C58A–C60A	109.2(14)
C61A–C58A–C59A	107.9(12)
C61A–C58A–C60A	108.2(11)
C58A–C59A–H59D	109.5
C58A–C59A–H59E	109.5
C58A–C59A–H59F	109.5
H59D–C59A–H59E	109.5
H59D–C59A–H59F	109.5
H59E–C59A–H59F	109.5
C58A–C60A–H60D	109.5
C58A–C60A–H60E	109.5
C58A–C60A–H60F	109.5
H60D–C60A–H60E	109.5
H60D–C60A–H60F	109.5
H60E–C60A–H60F	109.5
C58A–C61A–H61D	109.5
C58A–C61A–H61E	109.5
C58A–C61A–H61F	109.5
H61D–C61A–H61E	109.5
H61D–C61A–H61F	109.5
H61E–C61A–H61F	109.5
N22A–C76A–H76C	109.3
N22A–C76A–H76D	109.3
H76C–C76A–H76D	108.0
C77A–C76A–N22A	111.5(18)
C77A–C76A–H76C	109.3
C77A–C76A–H76D	109.3
C76A–C77A–H77D	109.5
C76A–C77A–H77E	109.5
C76A–C77A–H77F	109.5
H77D–C77A–H77E	109.5
H77D–C77A–H77F	109.5
H77E–C77A–H77F	109.5
N22A–C78A–H78C	108.1
N22A–C78A–H78D	108.1
H78C–C78A–H78D	107.3
C79A–C78A–N22A	116.7(16)

C79A–C78A–H78C	108.1
C79A–C78A–H78D	108.1
C78A–C79A–H79D	109.5
C78A–C79A–H79E	109.5
C78A–C79A–H79F	109.5
H79D–C79A–H79E	109.5
H79D–C79A–H79F	109.5
H79E–C79A–H79F	109.5
N23A–C80A–C81A	106.9(19)
N23A–C80A–C82A	108.0(15)
N23A–C80A–C83A	121.3(19)
C82A–C80A–C81A	107.8(16)
C83A–C80A–C81A	104.5(15)
C83A–C80A–C82A	107.7(16)
C80A–C81A–H81D	109.5
C80A–C81A–H81E	109.5
C80A–C81A–H81F	109.5
H81D–C81A–H81E	109.5
H81D–C81A–H81F	109.5
H81E–C81A–H81F	109.5
C80A–C82A–H82D	109.5
C80A–C82A–H82E	109.5
C80A–C82A–H82F	109.5
H82D–C82A–H82E	109.5
H82D–C82A–H82F	109.5
H82E–C82A–H82F	109.5
C80A–C83A–H83D	109.5
C80A–C83A–H83E	109.5
C80A–C83A–H83F	109.5
H83D–C83A–H83E	109.5
H83D–C83A–H83F	109.5
H83E–C83A–H83F	109.5
N23A–C84A–H84C	112.1
N23A–C84A–H84D	112.1
N23A–C84A–C85A	98.3(13)
H84C–C84A–H84D	109.8
C85A–C84A–H84C	112.1
C85A–C84A–H84D	112.1
N24A–C85A–C84A	104.8(10)
N24A–C85A–H85C	110.8
N24A–C85A–H85D	110.8
C84A–C85A–H85C	110.8
C84A–C85A–H85D	110.8
H85C–C85A–H85D	108.9
N24A–C86A–C87A	121.6(11)
N24A–C86A–C88A	103.7(10)
N24A–C86A–C89A	112.7(10)
C87A–C86A–C88A	101.0(10)
C87A–C86A–C89A	110.3(10)
C89A–C86A–C88A	105.2(8)
C86A–C87A–H87D	109.5
C86A–C87A–H87E	109.5
C86A–C87A–H87F	109.5

H87D–C87A–H87E	109.5
H87D–C87A–H87F	109.5
H87E–C87A–H87F	109.5
C86A–C88A–H88D	109.5
C86A–C88A–H88E	109.5
C86A–C88A–H88F	109.5
H88D–C88A–H88E	109.5
H88D–C88A–H88F	109.5
H88E–C88A–H88F	109.5
C86A–C89A–H89D	109.5
C86A–C89A–H89E	109.5
C86A–C89A–H89F	109.5
H89D–C89A–H89E	109.5
H89D–C89A–H89F	109.5
H89E–C89A–H89F	109.5
U2–C90A–H90C	106.0
U2–C90A–H90D	106.0
H90C–C90A–H90D	106.3
C91A–C90A–U2	125.4(10)
C91A–C90A–H90C	106.0
C91A–C90A–H90D	106.0
C90A–C91A–C93A	111.1(13)
C92A–C91A–C90A	120.5(16)
C92A–C91A–C93A	88.1(13)
C92A–C91A–C94A	117.5(17)
C94A–C91A–C90A	109.1(13)
C94A–C91A–C93A	107.9(16)
C91A–C92A–H92D	109.5
C91A–C92A–H92E	109.5
C91A–C92A–H92F	109.5
H92D–C92A–H92E	109.5
H92D–C92A–H92F	109.5
H92E–C92A–H92F	109.5
C91A–C93A–H93D	109.5
C91A–C93A–H93E	109.5
C91A–C93A–H93F	109.5
H93D–C93A–H93E	109.5
H93D–C93A–H93F	109.5
H93E–C93A–H93F	109.5
C91A–C94A–H94D	109.5
C91A–C94A–H94E	109.5
C91A–C94A–H94F	109.5
H94D–C94A–H94E	109.5
H94D–C94A–H94F	109.5
H94E–C94A–H94F	109.5

Table S7. Bond lengths for **3.**

Atom	Atom	Length/Å	Atom	Atom	Length/Å
U1	N1	2.182(4)	P4	N13	1.534(5)

Atom	Atom	Length/Å	Atom	Atom	Length/Å
U1	N5	2.179(4)	P4	N14	1.661(6)
U1	N9	2.161(3)	P4	N16	1.659(6)
U1	C1	2.506(5)	P4	N15	1.672(7)
P1	N1	1.542(4)	P5	N17	1.536(5)
P1	N2	1.663(4)	P5	N18	1.662(5)
P1	N3	1.690(4)	P5	N19	1.684(5)
P1	N4	1.673(4)	P5	N20	1.665(5)
P2	N5	1.540(4)	P6	N21	1.546(4)
P2	N6	1.661(4)	P6	N22	1.658(5)
P2	N7	1.674(4)	P6	N23	1.698(6)
P2	N8	1.699(4)	P6	N24	1.676(5)
P3	N9	1.540(4)	N14	C61	1.352(13)
P3	N10	1.673(4)	N14	C57	1.550(12)
P3	N11	1.686(4)	N16	C53	1.464(11)
P3	N12	1.682(4)	N16	C55	1.465(12)
N2	C6	1.454(7)	N18	C67	1.459(8)
N2	C8	1.462(6)	N18	C69	1.444(9)
N3	C10	1.493(6)	N19	C71	1.491(9)
N3	C14	1.460(6)	N19	C75	1.464(8)
N4	C15	1.451(7)	N20	C76	1.477(11)
N4	C16	1.481(6)	N20	C77	1.466(12)
N6	C20	1.457(7)	N22	C81	1.468(7)
N6	C22	1.459(7)	N22	C83	1.466(7)
N7	C24	1.479(7)	N23	C85	1.478(10)
N7	C28	1.448(6)	N23	C89	1.467(8)
N8	C29	1.457(7)	N24	C90	1.445(10)
N8	C30	1.491(7)	N24	C91	1.501(11)

Atom	Atom	Length/Å	Atom	Atom	Length/Å
N10	C34	1.460(6)	C48	C49	1.474(13)
N10	C36	1.459(6)	C49	C50	1.530(15)
N11	C38	1.496(6)	C49	C51	1.473(14)
N11	C42	1.462(6)	C49	C52	1.601(12)
N12	C43	1.468(6)	C49	C48A	1.471(12)
N12	C44	1.486(6)	C49	C50A	1.516(13)
C1	C2	1.536(7)	C49	C51A	1.474(19)
C2	C3	1.522(8)	C49	C52A	1.610(13)
C2	C4	1.529(9)	C63	C65	1.431(12)
C2	C5	1.524(9)	C63	C66	1.618(11)
C6	C7	1.489(8)	C63	N15	1.430(9)
C8	C9	1.506(8)	C63	C64	1.525(9)
C10	C11	1.528(7)	C61	C62	1.74(2)
C10	C12	1.529(7)	C62	N15	1.592(13)
C10	C13	1.521(7)	C57	C59	1.524(15)
C14	C15	1.512(7)	C57	C58	1.429(15)
C16	C17	1.526(7)	C57	C60	1.868(18)
C16	C18	1.533(7)	C53	C54	1.696(17)
C16	C19	1.533(7)	C67	C68	1.672(12)
C20	C21	1.524(7)	C67	C68A	1.693(16)
C22	C23	1.512(9)	C69	C70	1.347(13)
C24	C25	1.525(7)	C69	C70A	1.354(13)
C24	C26	1.516(8)	C71	C72	1.538(10)
C24	C27	1.536(7)	C71	C73	1.501(10)
C28	C29	1.515(8)	C71	C74	1.532(12)
C30	C31	1.534(7)	C75	C76	1.498(11)
C30	C32	1.523(7)	C77	C78	1.388(15)

Atom	Atom	Length/Å	Atom	Atom	Length/Å
C30	C33	1.539(7)	C77	C79	1.82(2)
C34	C35	1.506(8)	C77	C80	1.504(14)
C36	C37	1.514(8)	C77	C78A	1.751(18)
C38	C39	1.526(8)	C77	C79A	1.409(16)
C38	C40	1.522(8)	C81	C82	1.502(9)
C38	C41	1.532(7)	C83	C84	1.512(9)
C42	C43	1.508(7)	C85	C86	1.528(11)
C44	C45	1.524(7)	C85	C87	1.540(9)
C44	C46	1.533(7)	C85	C88	1.527(8)
C44	C47	1.529(7)	C89	C90	1.513(14)
U2	N13	2.171(4)	C91	C92	1.506(14)
U2	N17	2.179(4)	C91	C93	1.533(11)
U2	N21	2.177(4)	C91	C94	1.543(13)
U2	C48	2.498(11)	C56	C55	1.344(15)
U2	C48A	2.495(11)	C55	C56A	1.469(17)

Table S8. Bond angles for **3.**

Atom	Atom	Atom	Angle/˚	Atom	Atom	Atom	Angle/˚
N1	U1	C1	112.37(16)	N13	P4	N14	116.9(3)
N5	U1	N1	113.87(15)	N13	P4	N16	114.8(3)
N5	U1	C1	102.67(17)	N13	P4	N15	118.1(3)
N9	U1	N1	110.89(14)	N14	P4	N15	93.0(4)
N9	U1	N5	114.62(14)	N16	P4	N14	106.2(4)
N9	U1	C1	101.45(16)	N16	P4	N15	105.2(3)
N1	P1	N2	111.2(2)	N17	P5	N18	111.3(3)
N1	P1	N3	120.7(2)	N17	P5	N19	119.6(3)
N1	P1	N4	118.1(2)	N17	P5	N20	118.7(3)
N2	P1	N3	105.0(2)	N18	P5	N19	105.6(3)

Atom	Atom	Atom	Angle/˚	Atom	Atom	Atom	Angle/˚
N2	P1	N4	107.4(2)	N18	P5	N20	107.3(3)
N4	P1	N3	92.2(2)	N20	P5	N19	92.3(3)
N5	P2	N6	111.7(2)	N21	P6	N22	111.9(3)
N5	P2	N7	118.7(2)	N21	P6	N23	119.7(3)
N5	P2	N8	119.3(2)	N21	P6	N24	119.1(3)
N6	P2	N7	105.7(2)	N22	P6	N23	105.5(3)
N6	P2	N8	106.3(2)	N22	P6	N24	105.6(3)
N7	P2	N8	92.8(2)	N24	P6	N23	92.5(3)
N9	P3	N10	110.5(2)	P4	N13	U2	173.1(3)
N9	P3	N11	119.8(2)	C61	N14	P4	119.0(6)
N9	P3	N12	116.8(2)	C61	N14	C57	116.7(8)
N10	P3	N11	105.9(2)	C57	N14	P4	124.3(7)
N10	P3	N12	109.2(2)	C53	N16	P4	122.9(7)
N12	P3	N11	93.01(19)	C53	N16	C55	112.9(8)
P1	N1	U1	163.7(2)	C55	N16	P4	123.3(5)
C6	N2	P1	122.4(3)	P5	N17	U2	167.4(3)
C6	N2	C8	117.1(4)	C67	N18	P5	119.6(5)
C8	N2	P1	120.2(4)	C69	N18	P5	122.6(5)
C10	N3	P1	123.9(3)	C69	N18	C67	117.7(6)
C14	N3	P1	108.8(3)	C71	N19	P5	123.6(4)
C14	N3	C10	116.9(4)	C75	N19	P5	111.5(5)
C15	N4	P1	113.9(3)	C75	N19	C71	115.4(6)
C15	N4	C16	118.1(4)	C76	N20	P5	113.0(5)
C16	N4	P1	126.1(3)	C77	N20	P5	126.3(6)
P2	N5	U1	163.5(2)	C77	N20	C76	119.8(7)
C20	N6	P2	121.8(3)	P6	N21	U2	167.8(3)
C20	N6	C22	117.2(5)	C81	N22	P6	120.3(4)

Atom	Atom	Atom	Angle/˚	Atom	Atom	Atom	Angle/˚
C22	N6	P2	120.3(4)	C83	N22	P6	122.6(4)
C24	N7	P2	125.9(3)	C83	N22	C81	117.1(5)
C28	N7	P2	113.7(4)	C85	N23	P6	122.6(4)
C28	N7	C24	119.4(4)	C89	N23	P6	108.5(5)
C29	N8	P2	108.2(3)	C89	N23	C85	116.8(7)
C29	N8	C30	116.8(4)	C90	N24	P6	113.2(6)
C30	N8	P2	122.2(3)	C90	N24	C91	119.9(6)
P3	N9	U1	165.4(2)	C91	N24	P6	125.6(5)
C34	N10	P3	122.2(3)	C49	C48	U2	126.4(8)
C36	N10	P3	120.9(4)	C48	C49	C50	120.0(13)
C36	N10	C34	116.7(4)	C48	C49	C52	102.0(16)
C38	N11	P3	122.2(3)	C50	C49	C52	86.9(10)
C42	N11	P3	110.4(3)	C51	C49	C48	110.4(13)
C42	N11	C38	116.5(4)	C51	C49	C50	121.5(14)
C43	N12	P3	112.3(3)	C51	C49	C52	110.4(12)
C43	N12	C44	118.5(4)	C48A	C49	C50A	110.7(14)
C44	N12	P3	125.9(3)	C48A	C49	C51A	123.3(17)
C2	C1	U1	121.0(3)	C48A	C49	C52A	110.2(14)
C3	C2	C1	112.0(5)	C50A	C49	C52A	104.5(13)
C3	C2	C4	107.7(5)	C51A	C49	C50A	118.1(16)
C3	C2	C5	110.4(5)	C51A	C49	C52A	84.4(11)
C4	C2	C1	108.2(5)	C65	C63	C66	102.0(8)
C5	C2	C1	109.4(5)	C65	C63	C64	112.8(8)
C5	C2	C4	109.0(5)	N15	C63	C65	121.5(7)
N2	C6	C7	115.6(5)	N15	C63	C66	100.8(6)
N2	C8	C9	115.7(5)	N15	C63	C64	112.4(6)
N3	C10	C11	111.6(4)	C64	C63	C66	104.3(6)

Atom	Atom	Atom	Angle/˚	Atom	Atom	Atom	Angle/˚
N3	C10	C12	107.9(4)	N14	C61	C62	103.0(9)
N3	C10	C13	110.2(4)	N15	C62	C61	94.7(10)
C11	C10	C12	109.8(4)	N14	C57	C60	105.2(8)
C13	C10	C11	108.6(4)	C59	C57	N14	108.8(9)
C13	C10	C12	108.7(5)	C59	C57	C60	102.1(10)
N3	C14	C15	105.6(4)	C58	C57	N14	108.9(11)
N4	C15	C14	106.0(4)	C58	C57	C59	117.3(11)
N4	C16	C17	110.7(4)	C58	C57	C60	113.7(10)
N4	C16	C18	110.4(4)	N16	C53	C54	103.6(9)
N4	C16	C19	108.5(4)	N18	C67	C68	114.4(6)
C17	C16	C18	108.9(4)	N18	C67	C68A	104.4(9)
C17	C16	C19	110.2(4)	C70	C69	N18	131.2(12)
C19	C16	C18	108.1(4)	C70A	C69	N18	116.2(9)
N6	C20	C21	114.4(5)	N19	C71	C72	111.4(7)
N6	C22	C23	115.4(6)	N19	C71	C73	111.5(7)
N7	C24	C25	110.9(4)	N19	C71	C74	111.0(5)
N7	C24	C26	110.3(4)	C73	C71	C72	109.6(7)
N7	C24	C27	108.7(4)	C73	C71	C74	107.8(8)
C25	C24	C27	107.8(5)	C74	C71	C72	105.4(7)
C26	C24	C25	109.4(5)	N19	C75	C76	104.4(6)
C26	C24	C27	109.7(5)	N20	C76	C75	107.8(6)
N7	C28	C29	105.6(4)	N20	C77	C79	106.1(8)
N8	C29	C28	105.9(4)	N20	C77	C80	112.0(7)
N8	C30	C31	111.8(4)	N20	C77	C78A	96.9(10)
N8	C30	C32	110.2(4)	C78	C77	N20	129.9(14)
N8	C30	C33	108.5(4)	C78	C77	C79	99.2(12)
C31	C30	C33	109.3(4)	C78	C77	C80	109.2(10)

Atom	Atom	Atom	Angle/˚	Atom	Atom	Atom	Angle/˚
C32	C30	C31	108.5(4)	C80	C77	C79	92.7(11)
C32	C30	C33	108.5(4)	C80	C77	C78A	104.5(8)
N10	C34	C35	114.6(5)	C79A	C77	N20	108.9(9)
N10	C36	C37	115.1(5)	C79A	C77	C80	127.7(15)
N11	C38	C39	110.2(4)	C79A	C77	C78A	101.3(12)
N11	C38	C40	111.3(4)	N22	C81	C82	114.8(6)
N11	C38	C41	108.6(4)	N22	C83	C84	114.6(5)
C39	C38	C41	107.8(4)	N23	C85	C86	112.3(6)
C40	C38	C39	108.3(5)	N23	C85	C87	108.9(6)
C40	C38	C41	110.4(5)	N23	C85	C88	110.2(6)
N11	C42	C43	105.1(4)	C86	C85	C87	109.2(7)
N12	C43	C42	104.4(4)	C88	C85	C86	108.7(6)
N12	C44	C45	112.2(4)	C88	C85	C87	107.4(5)
N12	C44	C46	109.4(4)	N23	C89	C90	104.6(7)
N12	C44	C47	109.2(4)	N24	C90	C89	107.0(6)
C45	C44	C46	108.3(4)	N24	C91	C92	111.0(6)
C45	C44	C47	109.6(5)	N24	C91	C93	107.9(7)
C47	C44	C46	107.9(4)	N24	C91	C94	107.8(10)
N13	U2	N17	114.22(17)	C92	C91	C93	110.7(10)
N13	U2	N21	113.56(19)	C92	C91	C94	110.5(8)
N13	U2	C48	97.6(4)	C93	C91	C94	108.9(7)
N13	U2	C48A	99.0(5)	C49	C48A	U2	126.8(8)
N17	U2	C48	100.2(9)	C63	N15	P4	129.3(5)
N17	U2	C48A	115.1(5)	C63	N15	C62	104.4(8)
N21	U2	N17	114.95(18)	C62	N15	P4	109.1(6)
N21	U2	C48	114.3(7)	N16	C55	C56A	126.0(11)
N21	U2	C48A	97.8(8)	C56	C55	N16	112.5(14)

Table S9. Bond lengths for **4.**

Atom	Atom	Length/Å	Atom	Atom	Length/Å
U1	N1	2.1508(16)	N12	C39	1.463(3)
U1	N5	2.1664(17)	N12	C40	1.494(3)
U1	N9	2.1587(16)	C2	C3	1.508(3)
U1	C1	2.448(2)	C4	C5	1.524(3)
P1	N1	1.5433(17)	C6	C7	1.534(3)
P1	N2	1.6623(16)	C6	C8	1.523(3)
P1	N3	1.6871(17)	C6	C9	1.532(3)
P1	N4	1.6803(17)	C10	C11	1.523(6)
P2	N5	1.5440(18)	C10A	C11A	1.508(11)
P2	N6	1.6742(17)	C12	C13	1.532(3)
P2	N7	1.6663(18)	C12	C14	1.539(3)
P2	N8	1.7006(17)	C12	C15	1.523(3)
P3	N9	1.5482(17)	C16	C17	1.511(3)
P3	N10	1.6623(17)	C18	C19	1.517(3)
P3	N11	1.6676(17)	C20	C21	1.539(8)
P3	N12	1.6973(17)	C20	C21A	1.503(12)
N2	C2	1.459(2)	C20	C22	1.548(6)
N2	C4	1.463(2)	C20	C22A	1.541(11)
N3	C6	1.492(3)	C20	C23	1.507(7)
N3	C10	1.513(5)	C20	C23A	1.557(11)
N3	C10A	1.382(8)	C24	C25	1.510(3)
N4	C11	1.424(5)	C26	C27	1.526(3)
N4	C11A	1.548(9)	C26	C28	1.531(3)
N4	C12	1.488(3)	C26	C29	1.533(3)
N6	C16	1.464(3)	C30	C31	1.519(3)
N6	C18	1.465(3)	C32	C33	1.512(3)
N7	C20	1.489(3)	C34	C35	1.538(3)

Atom	Atom	Length/Å	Atom	Atom	Length/Å
N7	C24	1.455(3)	C34	C36	1.520(3)
N8	C25	1.463(2)	C34	C37	1.529(3)
N8	C26	1.489(3)	C38	C39	1.516(3)
N10	C30	1.464(3)	C40	C41	1.534(3)
N10	C32	1.464(2)	C40	C42	1.535(3)
N11	C34	1.484(3)	C40	C43	1.523(3)
N11	C38	1.456(3)			

Table S10. Bond angles for **4.**

Atom	Atom	Atom	Angle/˚	Atom	Atom	Atom	Angle/˚
N1	U1	N5	110.87(7)	C40	N12	P3	121.02(14)
N1	U1	N9	112.26(7)	N2	C2	C3	113.86(19)
N1	U1	C1	102.98(8)	N2	C4	C5	114.72(17)
N5	U1	C1	105.15(8)	N3	C6	C7	107.9(2)
N9	U1	N5	121.82(7)	N3	C6	C8	109.90(17)
N9	U1	C1	101.17(8)	N3	C6	C9	111.48(19)
N1	P1	N2	110.65(9)	C8	C6	C7	108.2(2)
N1	P1	N3	118.64(9)	C8	C6	C9	109.1(2)
N1	P1	N4	119.18(9)	C9	C6	C7	110.2(2)
N2	P1	N3	107.21(9)	N3	C10	C11	105.6(4)
N2	P1	N4	106.09(8)	N3	C10A	C11A	105.5(7)
N4	P1	N3	93.19(9)	N4	C11	C10	104.9(4)
N5	P2	N6	110.42(9)	C10A	C11A	N4	106.5(6)
N5	P2	N7	118.88(10)	N4	C12	C13	109.26(19)
N5	P2	N8	118.64(9)	N4	C12	C14	110.33(18)
N6	P2	N8	107.20(9)	N4	C12	C15	109.67(16)
N7	P2	N6	107.04(10)	C13	C12	C14	109.45(18)
N7	P2	N8	92.89(9)	C15	C12	C13	108.2(2)

Atom	Atom	Atom	Angle/˚	Atom	Atom	Atom	Angle/˚
N9	P3	N10	110.99(9)	C15	C12	C14	109.88(19)
N9	P3	N11	119.06(9)	N6	C16	C17	114.2(2)
N9	P3	N12	118.75(9)	N6	C18	C19	113.91(18)
N10	P3	N11	106.51(9)	N7	C20	C21	108.8(6)
N10	P3	N12	106.61(9)	N7	C20	C21A	109.6(9)
N11	P3	N12	92.92(9)	N7	C20	C22	111.6(3)
P1	N1	U1	171.99(11)	N7	C20	C22A	110.8(4)
C2	N2	P1	122.33(13)	N7	C20	C23	108.7(4)
C2	N2	C4	116.48(16)	N7	C20	C23A	109.5(7)
C4	N2	P1	120.90(13)	C21	C20	C22	105.4(5)
C6	N3	P1	122.65(14)	C21A	C20	C22A	114.4(9)
C6	N3	C10	116.7(2)	C21A	C20	C23A	107.4(8)
C10	N3	P1	106.9(3)	C22A	C20	C23A	104.9(7)
C10A	N3	P1	116.8(4)	C23	C20	C21	112.5(6)
C10A	N3	C6	117.6(4)	C23	C20	C22	109.9(5)
C11	N4	P1	115.3(2)	N7	C24	C25	105.73(17)
C11	N4	C12	117.4(2)	N8	C25	C24	104.84(17)
C11A	N4	P1	106.2(4)	N8	C26	C27	109.33(18)
C12	N4	P1	124.52(14)	N8	C26	C28	108.06(17)
C12	N4	C11A	117.9(4)	N8	C26	C29	112.17(18)
P2	N5	U1	162.83(12)	C27	C26	C28	107.97(19)
C16	N6	P2	121.21(14)	C27	C26	C29	109.7(2)
C16	N6	C18	115.98(17)	C28	C26	C29	109.55(19)
C18	N6	P2	118.69(14)	N10	C30	C31	114.13(19)
C20	N7	P2	127.76(15)	N10	C32	C33	116.01(18)
C24	N7	P2	113.33(14)	N11	C34	C35	108.72(19)
C24	N7	C20	118.57(18)	N11	C34	C36	110.61(18)

Atom	Atom	Atom	Angle/˚	Atom	Atom	Atom	Angle/˚
C25	N8	P2	109.68(13)	N11	C34	C37	109.97(18)
C25	N8	C26	116.41(16)	C36	C34	C35	107.9(2)
C26	N8	P2	123.08(13)	C36	C34	C37	109.5(2)
P3	N9	U1	168.32(11)	C37	C34	C35	110.1(2)
C30	N10	P3	122.80(13)	N11	C38	C39	106.20(17)
C32	N10	P3	120.65(14)	N12	C39	C38	105.39(18)
C32	N10	C30	116.35(17)	N12	C40	C41	107.6(2)
C34	N11	P3	126.30(14)	N12	C40	C42	111.99(19)
C38	N11	P3	113.65(14)	N12	C40	C43	110.02(17)
C38	N11	C34	119.18(17)	C41	C40	C42	109.4(2)
C39	N12	P3	109.13(14)	C43	C40	C41	108.6(2)
C39	N12	C40	116.22(17)	C43	C40	C42	109.1(2)

Table S11. Bond length for **5.**

Atom	Atom	Length/Å	Atom	Atom	Length/Å
U1	N1[1]	2.202(2)	C1	C2	1.526(5)
U1	N1[2]	2.202(2)	C11	C12	1.528(4)
U1	N1[3]	2.202(2)	C11	C14	1.534(5)
U1	N1	2.202(2)	C11	C13	1.536(4)
P1	N3	1.680(2)	C5	C7	1.533(4)
P1	N4	1.698(2)	C5	C8	1.539(4)
P1	N2	1.668(3)	C5	C6	1.524(4)
P1	N1	1.547(2)	C10	C9	1.520(4)
N3	C5	1.476(4)	C4	C3	1.527(4)
N3	C9	1.461(4)	C15	C16	1.550(19)
N4	C11	1.489(4)	C16	C17	1.43(3)
N4	C10	1.460(4)	C17	C18	1.48(3)
N2	C1	1.462(6)	C18	C19	1.48(4)
N2	C3	1.462(4)			

[1]1+Y,1-X,1-Z; [2]1-Y,+X,1-Z; [3]1-X,1-Y,+Z

Table S12. Bond angles for **5.**

Atom	Atom	Atom	Angle/˚	Atom	Atom	Atom	Angle/˚
N1[1]	U1	N1[2]	112.75(13)	N2	C1	C2	113.7(3)
N1[2]	U1	N1	107.86(7)	N4	C11	C12	110.5(2)
N1[2]	U1	N1[3]	107.86(7)	N4	C11	C14	112.1(2)

Atom	Atom	Atom	Angle/˚	Atom	Atom	Atom	Angle/˚
N1[3]	U1	N1	112.75(13)	N4	C11	C13	107.8(2)
N1[1]	U1	N1	107.86(7)	C12	C11	C14	108.9(3)
N1[1]	U1	N1[3]	107.86(7)	C12	C11	C13	107.9(2)
N3	P1	N4	91.71(12)	C14	C11	C13	109.5(3)
N2	P1	N3	105.35(13)	N3	C5	C7	109.3(3)
N2	P1	N4	106.11(13)	N3	C5	C8	108.9(2)
N1	P1	N3	119.39(13)	N3	C5	C6	111.4(2)
N1	P1	N4	119.67(14)	C7	C5	C8	110.4(3)
N1	P1	N2	112.03(13)	C6	C5	C7	108.6(2)
C5	N3	P1	127.6(2)	C6	C5	C8	108.4(3)
C9	N3	P1	113.95(19)	N4	C10	C9	104.9(3)
C9	N3	C5	118.1(2)	N2	C3	C4	114.4(3)
C11	N4	P1	124.00(19)	P1	N1	U1	164.32(16)
C10	N4	P1	108.72(19)	N3	C9	C10	105.3(2)
C10	N4	C11	116.2(2)	C17	C16	C15	129(2)
C1	N2	P1	122.44(18)	C16	C17	C18	129(2)
C3	N2	P1	120.0(2)	C19	C18	C17	147(6)
C3	N2	C1	117.5(2)				

[1]1+Y,1-X,1-Z; [2]1-Y,+X,1-Z; [3]1-X,1-Y,+Z

Table S13. Bond length for **6.**

Atom	Atom	Length/Å	Atom	Atom	Length/Å
U1	N1	2.100(9)	C54	C51	1.46(2)
U1	N3	2.131(10)	C21	C22	1.53(2)
U1	O1	2.530(9)	C78	F24	1.34(2)
U1	N2	2.136(11)	C78	C75	1.483(19)
P1	N1	1.567(10)	C48	C47	1.421(16)
P1	N4	1.658(11)	C48	C49	1.404(17)
P1	N5	1.677(10)	C64	C63	1.392(16)

Atom	Atom	Length/Å	Atom	Atom	Length/Å
P1	N6	1.655(10)	C64	C65	1.399(18)
P2	N9	1.677(10)	C63	C68	1.397(16)
P2	N8	1.684(12)	C63	B1	1.637(16)
P2	N7	1.666(11)	C11	C13	1.52(2)
P2	N2	1.564(11)	C11	C14	1.523(19)
P3	N12	1.697(11)	C11	C12	1.54(2)
P3	N10	1.659(13)	C47	B1	1.651(16)
P3	N11	1.667(11)	C47	C52	1.392(18)
P3	N3	1.580(10)	C74	C75	1.386(18)
F8	C61	1.346(16)	C74	C73	1.382(18)
F9	C61	1.331(15)	C46	C43	1.53(2)
F23	C78	1.301(18)	C50	C49	1.39(2)
F7	C61	1.324(15)	C50	C51	1.389(19)
F19	C77	1.339(16)	C58	C59	1.383(17)
F22	C78	1.28(2)	C65	C69	1.513(19)
F11	C62	1.308(15)	C65	C66	1.36(2)
F20	C77	1.341(16)	C76	C75	1.400(17)
F10	C62	1.337(15)	C76	C71	1.384(16)
F6	C54	1.370(19)	C60	C59	1.393(17)
N9	C24	1.439(19)	C15	C18	1.50(2)
N9	C29	1.484(19)	C15	C17	1.529(17)
N12	C38	1.457(16)	C62	C59	1.519(16)
N12	C43	1.486(17)	O1	C3	1.423(17)
N10	C35	1.483(19)	O1	C1	1.460(18)
N10	C33	1.438(17)	C31	C29	1.54(2)
F15	C69	1.318(18)	C68	C67	1.398(17)
C23	N8	1.462(17)	B1	C71	1.647(17)

Atom	Atom	Length/Å	Atom	Atom	Length/Å
C23	C24	1.51(2)	C27	C25	1.556(17)
F14	C69	1.331(17)	C45	C43	1.50(2)
N4	C7	1.490(15)	C25	C26	1.53(2)
N4	C5	1.478(17)	C25	C28	1.501(19)
C56	C55	1.407(16)	C73	C72	1.393(16)
C56	C57	1.393(17)	C73	C77	1.489(19)
N8	C25	1.487(16)	C29	C30	1.53(2)
F21	C77	1.362(16)	C29	C32	1.54(2)
N11	C39	1.483(19)	C72	C71	1.398(17)
N11	C37	1.443(19)	C5	C6	1.51(2)
F12	C62	1.310(15)	C10	C9	1.47(2)
F1	C53	1.316(16)	F13	C69	1.332(19)
F2	C53	1.303(16)	C67	C66	1.404(18)
C55	C60	1.407(17)	C67	C70	1.492(18)
C55	B1	1.649(18)	C52	C51	1.416(18)
C53	F3	1.317(17)	C36	C35	1.54(2)
C53	C49	1.486(18)	C3	C4	1.49(3)
N5	C11	1.502(18)	C1	C2	1.50(2)
N5	C9	1.463(18)	C41	C39	1.56(3)
N7	C21	1.462(15)	C38	C37	1.48(2)
N7	C19	1.465(19)	C19	C20	1.38(2)
C7	C8	1.520(19)	C44	C43	1.539(19)
C61	C57	1.515(16)	C42	C39	1.55(3)
N6	C15	1.503(15)	C39	C40A	1.49(3)
N6	C10	1.444(18)	C39	C41A	1.54(3)
C57	C58	1.407(18)	C39	C42A	1.58(3)
F4	C54	1.350(18)	F17	C70	1.319(15)

Atom	Atom	Length/Å	Atom	Atom	Length/Å
C16	C15	1.543(19)	F18	C70	1.317(16)
C40	C39	1.48(3)	F16	C70	1.323(16)
C54	F5	1.310(19)	C34	C33	1.52(2)

Table S14. Bond angles for **6.**

Atom	Atom	Atom	Angle/˚	Atom	Atom	Atom	Angle/˚
N1	U1	N3	106.8(4)	C71	C76	C75	123.4(11)
N1	U1	O1	106.5(3)	C59	C60	C55	122.9(11)
N1	U1	N2	112.6(4)	N6	C15	C16	110.0(11)
N3	U1	O1	115.6(4)	N6	C15	C17	109.4(10)
N3	U1	N2	112.6(4)	C18	C15	N6	110.6(10)
N2	U1	O1	102.7(4)	C18	C15	C16	108.4(12)
N1	P1	N4	109.3(5)	C18	C15	C17	109.0(12)
N1	P1	N5	118.9(5)	C17	C15	C16	109.4(11)
N1	P1	N6	117.4(5)	F11	C62	F10	106.3(12)
N4	P1	N5	106.6(5)	F11	C62	F12	105.1(11)
N6	P1	N4	110.2(6)	F11	C62	C59	112.5(10)
N6	P1	N5	93.2(5)	F10	C62	C59	113.2(10)
N9	P2	N8	93.3(6)	F12	C62	F10	106.3(11)
N7	P2	N9	109.8(5)	F12	C62	C59	112.9(11)
N7	P2	N8	106.0(6)	C3	O1	U1	120.2(9)
N2	P2	N9	117.9(6)	C3	O1	C1	113.2(11)
N2	P2	N8	119.2(5)	C1	O1	U1	126.5(8)
N2	P2	N7	109.3(5)	C63	C68	C67	121.6(11)
N10	P3	N12	106.7(6)	C74	C75	C78	119.3(12)
N10	P3	N11	108.4(6)	C74	C75	C76	119.4(11)
N11	P3	N12	93.0(6)	C76	C75	C78	121.3(12)
N3	P3	N12	119.2(6)	C55	B1	C47	114.0(9)

Atom	Atom	Atom	Angle/˚	Atom	Atom	Atom	Angle/˚
N3	P3	N10	110.9(6)	C63	B1	C55	102.2(8)
N3	P3	N11	116.9(6)	C63	B1	C47	114.4(10)
P1	N1	U1	175.4(6)	C63	B1	C71	112.8(8)
C24	N9	P2	113.4(10)	C71	B1	C55	110.6(11)
C24	N9	C29	117.5(10)	C71	B1	C47	103.2(8)
C29	N9	P2	128.3(10)	N8	C25	C27	110.9(11)
C38	N12	P3	111.7(9)	N8	C25	C26	109.8(11)
C38	N12	C43	117.2(11)	N8	C25	C28	111.2(11)
C43	N12	P3	124.2(9)	C26	C25	C27	108.0(12)
C35	N10	P3	120.2(10)	C28	C25	C27	109.6(11)
C33	N10	P3	124.1(10)	C28	C25	C26	107.3(12)
C33	N10	C35	114.2(12)	C74	C73	C72	120.2(12)
N8	C23	C24	106.2(11)	C74	C73	C77	119.5(11)
C7	N4	P1	119.7(9)	C72	C73	C77	120.2(12)
C5	N4	P1	124.0(9)	N9	C29	C31	109.6(13)
C5	N4	C7	115.9(11)	N9	C29	C30	111.1(11)
C57	C56	C55	121.9(11)	N9	C29	C32	110.1(12)
C23	N8	P2	109.8(9)	C30	C29	C31	109.6(13)
C23	N8	C25	115.8(11)	C30	C29	C32	108.2(14)
C25	N8	P2	125.3(9)	C32	C29	C31	108.1(14)
C39	N11	P3	125.0(9)	C73	C72	C71	122.6(12)
C37	N11	P3	114.0(10)	C76	C71	B1	123.2(11)
C37	N11	C39	120.7(11)	C76	C71	C72	115.4(11)
C56	C55	B1	123.4(10)	C72	C71	B1	120.9(10)
C60	C55	C56	115.5(11)	N4	C5	C6	112.9(12)
C60	C55	B1	120.6(10)	N6	C10	C9	108.8(12)
F1	C53	F3	107.6(12)	C68	C67	C66	120.0(12)

Atom	Atom	Atom	Angle/˚	Atom	Atom	Atom	Angle/˚
F1	C53	C49	112.8(11)	C68	C67	C70	119.3(11)
F2	C53	F1	103.9(13)	C66	C67	C70	120.7(12)
F2	C53	F3	106.0(13)	C47	C52	C51	123.6(12)
F2	C53	C49	113.6(12)	N5	C9	C10	107.6(13)
F3	C53	C49	112.3(12)	C48	C49	C53	119.2(12)
C11	N5	P1	123.6(9)	C50	C49	C53	119.1(11)
C9	N5	P1	111.1(9)	C50	C49	C48	121.6(11)
C9	N5	C11	117.0(11)	O1	C3	C4	110.2(12)
C21	N7	P2	119.4(8)	P2	N2	U1	173.5(7)
C21	N7	C19	117.1(11)	F15	C69	F14	103.8(12)
C19	N7	P2	123.0(9)	F15	C69	C65	112.8(14)
N4	C7	C8	112.8(11)	F15	C69	F13	107.4(13)
F8	C61	C57	111.3(10)	F14	C69	C65	113.2(12)
F9	C61	F8	107.9(10)	F14	C69	F13	106.3(15)
F9	C61	C57	113.1(11)	F13	C69	C65	112.7(12)
F7	C61	F8	105.8(12)	O1	C1	C2	113.1(13)
F7	C61	F9	107.6(10)	C58	C59	C60	120.6(11)
F7	C61	C57	110.9(11)	C58	C59	C62	120.8(11)
C15	N6	P1	125.9(8)	C60	C59	C62	118.6(11)
C10	N6	P1	113.8(9)	N12	C38	C37	107.7(12)
C10	N6	C15	118.0(10)	C50	C51	C54	119.6(13)
C56	C57	C61	118.1(11)	C50	C51	C52	119.6(13)
C56	C57	C58	121.0(11)	C52	C51	C54	120.8(13)
C58	C57	C61	120.9(11)	F19	C77	F20	107.7(12)
F6	C54	C51	113.2(13)	F19	C77	F21	105.8(12)
F4	C54	F6	102.6(13)	F19	C77	C73	112.5(10)
F4	C54	C51	114.1(14)	F20	C77	F21	105.5(10)

Atom	Atom	Atom	Angle/˚	Atom	Atom	Atom	Angle/˚
F5	C54	F6	105.6(15)	F20	C77	C73	111.8(12)
F5	C54	F4	103.9(13)	F21	C77	C73	113.0(12)
F5	C54	C51	116.0(14)	C20	C19	N7	119.7(18)
N9	C24	C23	105.3(12)	N12	C43	C46	107.8(11)
P3	N3	U1	161.2(6)	N12	C43	C45	111.5(13)
N7	C21	C22	114.9(11)	N12	C43	C44	107.8(11)
F23	C78	F24	106.3(15)	C46	C43	C44	108.3(13)
F23	C78	C75	115.7(14)	C45	C43	C46	110.6(13)
F22	C78	F23	105.2(15)	C45	C43	C44	110.8(12)
F22	C78	F24	102.6(16)	C65	C66	C67	119.3(13)
F22	C78	C75	113.0(15)	N11	C39	C41	107.7(15)
F24	C78	C75	112.9(15)	N11	C39	C42	107.9(14)
C49	C48	C47	121.4(12)	N11	C39	C40A	111(2)
C63	C64	C65	122.7(11)	N11	C39	C41A	114(2)
C64	C63	C68	116.3(10)	N11	C39	C42A	109.2(19)
C64	C63	B1	123.2(9)	C40	C39	N11	113.1(13)
C68	C63	B1	120.1(10)	C40	C39	C41	107.8(19)
N5	C11	C13	110.7(11)	C40	C39	C42	114(2)
N5	C11	C14	110.4(13)	C42	C39	C41	106.4(16)
N5	C11	C12	111.0(12)	C40A	C39	C41A	109(3)
C13	C11	C14	106.6(13)	C40A	C39	C42A	109(3)
C13	C11	C12	110.3(14)	C41A	C39	C42A	104(2)
C14	C11	C12	107.7(13)	N11	C37	C38	108.0(12)
C48	C47	B1	120.2(11)	N10	C35	C36	116.1(14)
C52	C47	C48	115.3(11)	N10	C33	C34	114.4(13)
C52	C47	B1	124.4(10)	F17	C70	C67	112.3(11)
C73	C74	C75	119.0(12)	F17	C70	F16	104.9(12)

Atom	Atom	Atom	Angle/˚	Atom	Atom	Atom	Angle/˚
C49	C50	C51	118.5(12)	F18	C70	C67	113.5(11)
C59	C58	C57	118.0(11)	F18	C70	F17	106.2(13)
C64	C65	C69	121.1(12)	F18	C70	F16	105.7(13)
C66	C65	C64	120.0(12)	F16	C70	C67	113.6(12)
C66	C65	C69	118.9(13)				

Table S15. Bond length for **7-H.**

Atom–Atom	Length [Å]
U1–I1	2.902(12)
U1–N1	2.139(6)
U1–N5	2.161(6)
U1–N9	2.153(5)
P1–N1	1.529(6)
P1–N2	1.660(5)
P1–N4	1.687(4)
P1–N3	1.667(18)
P1–N3A	1.691(15)
P2–N5	1.535(6)
P2–N6	1.671(5)
P2–N8	1.685(5)
P2–N7	1.672(15)
P2–N7A	1.65(4)
P3–N9	1.543(5)
P3–N10	1.656(5)
P3–N12	1.690(5)
P3–N11	1.676(10)
P3–N11A	1.66(4)
N2–C1	1.478(7)
N2–C3	1.457(8)

N4–C11	1.492(8)
N4–C10	1.36(2)
N4–C10A	1.53(2)
N6–C15	1.450(9)
N6–C17	1.476(9)
N8–C24	1.452(8)
N8–C25	1.496(7)
N10–C29	1.461(9)
N10–C31	1.479(7)
N12–C38	1.460(9)
N12–C39	1.487(8)
C1–H1A	0.9900
C1–H1B	0.9900
C1–C2	1.478(9)
C2–H2A	0.9800
C2–H2B	0.9800
C2–H2C	0.9800
C3–H3A	0.9900
C3–H3B	0.9900
C3–C4	1.516(9)
C4–H4A	0.9800
C4–H4B	0.9800
C4–H4C	0.9800
C11–C12	1.549(9)
C11–C13	1.514(10)
C11–C14	1.497(9)
C12–H12A	0.9800
C12–H12B	0.9800
C12–H12C	0.9800
C13–H13A	0.9800

C13–H13B	0.9800
C13–H13C	0.9800
C14–H14A	0.9800
C14–H14B	0.9800
C14–H14C	0.9800
C15–H15A	0.9900
C15–H15B	0.9900
C15–C16	1.484(11)
C16–H16A	0.9800
C16–H16B	0.9800
C16–H16C	0.9800
C17–H17A	0.9900
C17–H17B	0.9900
C17–C18	1.515(9)
C18–H18A	0.9800
C18–H18B	0.9800
C18–H18C	0.9800
C23–H23A	0.9900
C23–H23B	0.9900
C23–H23C	0.9900
C23–H23D	0.9900
C23–C24	1.511(8)
C23–N7	1.437(18)
C23–N7A	1.49(4)
C24–H24A	0.9900
C24–H24B	0.9900
C25–C26	1.507(10)
C25–C27	1.533(10)
C25–C28	1.544(9)
C26–H26A	0.9800

C26–H26B	0.9800
C26–H26C	0.9800
C27–H27A	0.9800
C27–H27B	0.9800
C27–H27C	0.9800
C28–H28A	0.9800
C28–H28B	0.9800
C28–H28C	0.9800
C29–H29A	0.9900
C29–H29B	0.9900
C29–C30	1.516(9)
C30–H30A	0.9800
C30–H30B	0.9800
C30–H30C	0.9800
C31–H31A	0.9900
C31–H31B	0.9900
C31–C32	1.501(10)
C32–H32A	0.9800
C32–H32B	0.9800
C32–H32C	0.9800
C37–H37A	0.9900
C37–H37B	0.9900
C37–H37C	0.9900
C37–H37D	0.9900
C37–C38	1.502(9)
C37–N11	1.444(12)
C37–N11A	1.54(4)
C38–H38A	0.9900
C38–H38B	0.9900
C39–C40	1.540(9)

C39–C41	1.544(9)
C39–C42	1.509(11)
C40–H40A	0.9800
C40–H40B	0.9800
C40–H40C	0.9800
C41–H41A	0.9800
C41–H41B	0.9800
C41–H41C	0.9800
C42–H42A	0.9800
C42–H42B	0.9800
C42–H42C	0.9800
N3–C5	1.503(13)
N3–C9	1.480(15)
C5–C6	1.549(15)
C5–C7	1.523(15)
C5–C8	1.503(15)
C6–H6A	0.9800
C6–H6B	0.9800
C6–H6C	0.9800
C7–H7A	0.9800
C7–H7B	0.9800
C7–H7C	0.9800
C8–H8A	0.9800
C8–H8B	0.9800
C8–H8C	0.9800
C9–H9A	0.9900
C9–H9B	0.9900
C9–C10	1.495(18)
C10–H10A	0.9900
C10–H10B	0.9900

N3A–C5A	1.496(11)
N3A–C9A	1.461(14)
C5A–C6A	1.553(13)
C5A–C7A	1.514(13)
C5A–C8A	1.524(13)
C6A–H6AA	0.9800
C6A–H6AB	0.9800
C6A–H6AC	0.9800
C7A–H7AA	0.9800
C7A–H7AB	0.9800
C7A–H7AC	0.9800
C8A–H8AA	0.9800
C8A–H8AB	0.9800
C8A–H8AC	0.9800
C9A–H9AA	0.9900
C9A–H9AB	0.9900
C9A–C10A	1.498(19)
C10A–H10C	0.9900
C10A–H10D	0.9900
N7–C19	1.489(10)
C19–C20	1.528(13)
C19–C22	1.532(12)
C19–C21	1.515(13)
C20–H20A	0.9800
C20–H20B	0.9800
C20–H20C	0.9800
C22–H22A	0.9800
C22–H22B	0.9800
C22–H22C	0.9800
C21–H21A	0.9800

C21–H21B	0.9800
C21–H21C	0.9800
N7A–C19A	1.493(15)
C19A–C20A	1.518(18)
C19A–C22A	1.537(17)
C19A–C21A	1.513(18)
C20A–H20D	0.9800
C20A–H20E	0.9800
C20A–H20F	0.9800
C22A–H22D	0.9800
C22A–H22E	0.9800
C22A–H22F	0.9800
C21A–H21D	0.9800
C21A–H21E	0.9800
C21A–H21F	0.9800
N11–C33	1.486(9)
C33–C34	1.530(15)
C33–C35	1.519(13)
C33–C36	1.539(14)
C34–H34A	0.9800
C34–H34B	0.9800
C34–H34C	0.9800
C35–H35A	0.9800
C35–H35B	0.9800
C35–H35C	0.9800
C36–H36A	0.9800
C36–H36B	0.9800
C36–H36C	0.9800
N11A–C33A	1.491(18)
C33A–C34A	1.53(3)

C33A–C35A	1.52(2)
C33A–C36A	1.54(2)
C34A–H34D	0.9800
C34A–H34E	0.9800
C34A–H34F	0.9800
C35A–H35D	0.9800
C35A–H35E	0.9800
C35A–H35F	0.9800
C36A–H36D	0.9800
C36A–H36E	0.9800
C36A–H36F	0.9800

Table S16. Bond angles for **7-H**.

**Atom–Atom–Atom**	**Angle [°]**
N1–U1–I1	100.1(4)
N1–U1–N5	109.62(19)
N1–U1–N9	109.2(2)
N5–U1–I1	119.0(4)
N9–U1–I1	97.0(3)
N9–U1–N5	119.7(2)
N1–P1–N2	110.7(3)
N1–P1–N4	119.9(3)
N1–P1–N3	119.1(10)
N1–P1–N3A	117.6(9)
N2–P1–N4	105.4(3)
N2–P1–N3	110.8(11)
N2–P1–N3A	105.1(9)
N4–P1–N3A	96.2(5)
N3–P1–N4	88.7(6)
N5–P2–N6	110.6(3)
N5–P2–N8	118.5(3)
N5–P2–N7	121.3(5)
N5–P2–N7A	112.3(13)
N6–P2–N8	107.2(3)
N6–P2–N7	104.6(5)
N7–P2–N8	92.5(5)
N7A–P2–N6	114.0(13)
N7A–P2–N8	93.5(12)
N9–P3–N10	110.7(3)
N9–P3–N12	119.2(3)

N9–P3–N11	120.7(4)
N9–P3–N11A	107.3(11)
N10–P3–N12	106.7(3)
N10–P3–N11	104.4(4)
N11–P3–N12	92.9(4)
N11A–P3–N10	117.8(11)
N11A–P3–N12	94.8(12)
P1–N1–U1	171.7(3)
C1–N2–P1	120.8(5)
C3–N2–P1	121.8(4)
C3–N2–C1	117.0(5)
C11–N4–P1	122.8(4)
C11–N4–C10A	121.0(8)
C10–N4–P1	119.0(8)
C10–N4–C11	114.6(9)
C10A–N4–P1	105.3(8)
P2–N5–U1	164.4(3)
C15–N6–P2	122.0(5)
C15–N6–C17	115.7(5)
C17–N6–P2	118.5(5)
C24–N8–P2	110.1(4)
C24–N8–C25	116.4(5)
C25–N8–P2	123.3(5)
P3–N9–U1	164.5(4)
C29–N10–P3	123.4(4)
C29–N10–C31	116.0(5)
C31–N10–P3	120.4(4)
C38–N12–P3	109.3(4)
C38–N12–C39	117.1(5)
C39–N12–P3	122.1(4)
N2–C1–H1A	108.9
N2–C1–H1B	108.9
N2–C1–C2	113.3(6)
H1A–C1–H1B	107.7
C2–C1–H1A	108.9
C2–C1–H1B	108.9
C1–C2–H2A	109.5
C1–C2–H2B	109.5
C1–C2–H2C	109.5
H2A–C2–H2B	109.5
H2A–C2–H2C	109.5
H2B–C2–H2C	109.5
N2–C3–H3A	108.6
N2–C3–H3B	108.6
N2–C3–C4	114.7(5)
H3A–C3–H3B	107.6
C4–C3–H3A	108.6
C4–C3–H3B	108.6
C3–C4–H4A	109.5
C3–C4–H4B	109.5
C3–C4–H4C	109.5
H4A–C4–H4B	109.5
H4A–C4–H4C	109.5

H4B–C4–H4C	109.5
N4–C11–C12	107.6(6)
N4–C11–C13	110.3(5)
N4–C11–C14	110.0(5)
C13–C11–C12	109.0(6)
C14–C11–C12	108.0(6)
C14–C11–C13	111.9(6)
C11–C12–H12A	109.5
C11–C12–H12B	109.5
C11–C12–H12C	109.5
H12A–C12–H12B	109.5
H12A–C12–H12C	109.5
H12B–C12–H12C	109.5
C11–C13–H13A	109.5
C11–C13–H13B	109.5
C11–C13–H13C	109.5
H13A–C13–H13B	109.5
H13A–C13–H13C	109.5
H13B–C13–H13C	109.5
C11–C14–H14A	109.5
C11–C14–H14B	109.5
C11–C14–H14C	109.5
H14A–C14–H14B	109.5
H14A–C14–H14C	109.5
H14B–C14–H14C	109.5
N6–C15–H15A	108.6
N6–C15–H15B	108.6
N6–C15–C16	114.8(6)
H15A–C15–H15B	107.5
C16–C15–H15A	108.6
C16–C15–H15B	108.6
C15–C16–H16A	109.5
C15–C16–H16B	109.5
C15–C16–H16C	109.5
H16A–C16–H16B	109.5
H16A–C16–H16C	109.5
H16B–C16–H16C	109.5
N6–C17–H17A	108.7
N6–C17–H17B	108.7
N6–C17–C18	114.3(6)
H17A–C17–H17B	107.6
C18–C17–H17A	108.7
C18–C17–H17B	108.7
C17–C18–H18A	109.5
C17–C18–H18B	109.5
C17–C18–H18C	109.5
H18A–C18–H18B	109.5
H18A–C18–H18C	109.5
H18B–C18–H18C	109.5
H23A–C23–H23B	108.5
H23C–C23–H23D	109.2
C24–C23–H23A	110.3
C24–C23–H23B	110.3

C24–C23–H23C	111.3
C24–C23–H23D	111.3
N7–C23–H23A	110.3
N7–C23–H23B	110.3
N7–C23–C24	107.2(7)
N7A–C23–H23C	111.3
N7A–C23–H23D	111.3
N7A–C23–C24	102.3(12)
N8–C24–C23	104.6(5)
N8–C24–H24A	110.8
N8–C24–H24B	110.8
C23–C24–H24A	110.8
C23–C24–H24B	110.8
H24A–C24–H24B	108.9
N8–C25–C26	109.5(5)
N8–C25–C27	107.8(6)
N8–C25–C28	112.0(5)
C26–C25–C27	108.5(6)
C26–C25–C28	109.8(6)
C27–C25–C28	109.3(6)
C25–C26–H26A	109.5
C25–C26–H26B	109.5
C25–C26–H26C	109.5
H26A–C26–H26B	109.5
H26A–C26–H26C	109.5
H26B–C26–H26C	109.5
C25–C27–H27A	109.5
C25–C27–H27B	109.5
C25–C27–H27C	109.5
H27A–C27–H27B	109.5
H27A–C27–H27C	109.5
H27B–C27–H27C	109.5
C25–C28–H28A	109.5
C25–C28–H28B	109.5
C25–C28–H28C	109.5
H28A–C28–H28B	109.5
H28A–C28–H28C	109.5
H28B–C28–H28C	109.5
N10–C29–H29A	108.9
N10–C29–H29B	108.9
N10–C29–C30	113.5(7)
H29A–C29–H29B	107.7
C30–C29–H29A	108.9
C30–C29–H29B	108.9
C29–C30–H30A	109.5
C29–C30–H30B	109.5
C29–C30–H30C	109.5
H30A–C30–H30B	109.5
H30A–C30–H30C	109.5
H30B–C30–H30C	109.5
N10–C31–H31A	108.3
N10–C31–H31B	108.3
N10–C31–C32	116.1(6)

H31A–C31–H31B	107.4
C32–C31–H31A	108.3
C32–C31–H31B	108.3
C31–C32–H32A	109.5
C31–C32–H32B	109.5
C31–C32–H32C	109.5
H32A–C32–H32B	109.5
H32A–C32–H32C	109.5
H32B–C32–H32C	109.5
H37A–C37–H37B	108.6
H37C–C37–H37D	109.4
C38–C37–H37A	110.3
C38–C37–H37B	110.3
C38–C37–H37C	111.6
C38–C37–H37D	111.6
C38–C37–N11A	100.7(12)
N11–C37–H37A	110.3
N11–C37–H37B	110.3
N11–C37–C38	107.0(6)
N11A–C37–H37C	111.6
N11A–C37–H37D	111.6
N12–C38–C37	106.8(5)
N12–C38–H38A	110.4
N12–C38–H38B	110.4
C37–C38–H38A	110.4
C37–C38–H38B	110.4
H38A–C38–H38B	108.6
N12–C39–C40	107.6(5)
N12–C39–C41	111.2(6)
N12–C39–C42	109.9(5)
C40–C39–C41	109.2(5)
C42–C39–C40	109.2(6)
C42–C39–C41	109.6(6)
C39–C40–H40A	109.5
C39–C40–H40B	109.5
C39–C40–H40C	109.5
H40A–C40–H40B	109.5
H40A–C40–H40C	109.5
H40B–C40–H40C	109.5
C39–C41–H41A	109.5
C39–C41–H41B	109.5
C39–C41–H41C	109.5
H41A–C41–H41B	109.5
H41A–C41–H41C	109.5
H41B–C41–H41C	109.5
C39–C42–H42A	109.5
C39–C42–H42B	109.5
C39–C42–H42C	109.5
H42A–C42–H42B	109.5
H42A–C42–H42C	109.5
H42B–C42–H42C	109.5
C5–N3–P1	123.0(12)
C9–N3–P1	110.6(12)

C9–N3–C5	115.0(13)
N3–C5–C6	106.6(12)
N3–C5–C7	108.4(11)
C7–C5–C6	107.8(13)
C8–C5–N3	110.3(14)
C8–C5–C6	113.1(14)
C8–C5–C7	110.4(13)
C5–C6–H6A	109.5
C5–C6–H6B	109.5
C5–C6–H6C	109.5
H6A–C6–H6B	109.5
H6A–C6–H6C	109.5
H6B–C6–H6C	109.5
C5–C7–H7A	109.5
C5–C7–H7B	109.5
C5–C7–H7C	109.5
H7A–C7–H7B	109.5
H7A–C7–H7C	109.5
H7B–C7–H7C	109.5
C5–C8–H8A	109.5
C5–C8–H8B	109.5
C5–C8–H8C	109.5
H8A–C8–H8B	109.5
H8A–C8–H8C	109.5
H8B–C8–H8C	109.5
N3–C9–H9A	110.7
N3–C9–H9B	110.7
N3–C9–C10	105.1(13)
H9A–C9–H9B	108.8
C10–C9–H9A	110.7
C10–C9–H9B	110.7
N4–C10–C9	105.0(15)
N4–C10–H10A	110.7
N4–C10–H10B	110.7
C9–C10–H10A	110.7
C9–C10–H10B	110.7
H10A–C10–H10B	108.8
C5A–N3A–P1	124.8(11)
C9A–N3A–P1	112.8(9)
C9A–N3A–C5A	120.0(11)
N3A–C5A–C6A	107.7(11)
N3A–C5A–C7A	111.1(10)
N3A–C5A–C8A	110.2(11)
C7A–C5A–C6A	109.5(10)
C7A–C5A–C8A	109.7(10)
C8A–C5A–C6A	108.6(10)
C5A–C6A–H6AA	109.5
C5A–C6A–H6AB	109.5
C5A–C6A–H6AC	109.5
H6AA–C6A–H6AB	109.5
H6AA–C6A–H6AC	109.5
H6AB–C6A–H6AC	109.5
C5A–C7A–H7AA	109.5

C5A–C7A–H7AB	109.5
C5A–C7A–H7AC	109.5
H7AA–C7A–H7AB	109.5
H7AA–C7A–H7AC	109.5
H7AB–C7A–H7AC	109.5
C5A–C8A–H8AA	109.5
C5A–C8A–H8AB	109.5
C5A–C8A–H8AC	109.5
H8AA–C8A–H8AB	109.5
H8AA–C8A–H8AC	109.5
H8AB–C8A–H8AC	109.5
N3A–C9A–H9AA	110.5
N3A–C9A–H9AB	110.5
N3A–C9A–C10A	106.0(13)
H9AA–C9A–H9AB	108.7
C10A–C9A–H9AA	110.5
C10A–C9A–H9AB	110.5
N4–C10A–H10C	109.8
N4–C10A–H10D	109.8
C9A–C10A–N4	109.2(13)
C9A–C10A–H10C	109.8
C9A–C10A–H10D	109.8
H10C–C10A–H10D	108.3
C23–N7–P2	113.2(7)
C23–N7–C19	118.7(11)
C19–N7–P2	128.0(11)
N7–C19–C20	108.6(9)
N7–C19–C22	109.1(9)
N7–C19–C21	111.2(9)
C20–C19–C22	111.9(10)
C21–C19–C20	108.9(10)
C21–C19–C22	107.3(10)
C19–C20–H20A	109.5
C19–C20–H20B	109.5
C19–C20–H20C	109.5
H20A–C20–H20B	109.5
H20A–C20–H20C	109.5
H20B–C20–H20C	109.5
C19–C22–H22A	109.5
C19–C22–H22B	109.5
C19–C22–H22C	109.5
H22A–C22–H22B	109.5
H22A–C22–H22C	109.5
H22B–C22–H22C	109.5
C19–C21–H21A	109.5
C19–C21–H21B	109.5
C19–C21–H21C	109.5
H21A–C21–H21B	109.5
H21A–C21–H21C	109.5
H21B–C21–H21C	109.5
C23–N7A–P2	111.8(15)
C23–N7A–C19A	116(3)
C19A–N7A–P2	128(3)

N7A–C19A–C20A	108.7(17)
N7A–C19A–C22A	109.3(18)
N7A–C19A–C21A	109.9(18)
C20A–C19A–C22A	111.0(18)
C21A–C19A–C20A	110.3(18)
C21A–C19A–C22A	107.6(17)
C19A–C20A–H20D	109.5
C19A–C20A–H20E	109.5
C19A–C20A–H20F	109.5
H20D–C20A–H20E	109.5
H20D–C20A–H20F	109.5
H20E–C20A–H20F	109.5
C19A–C22A–H22D	109.5
C19A–C22A–H22E	109.5
C19A–C22A–H22F	109.5
H22D–C22A–H22E	109.5
H22D–C22A–H22F	109.5
H22E–C22A–H22F	109.5
C19A–C21A–H21D	109.5
C19A–C21A–H21E	109.5
C19A–C21A–H21F	109.5
H21D–C21A–H21E	109.5
H21D–C21A–H21F	109.5
H21E–C21A–H21F	109.5
C37–N11–P3	113.7(5)
C37–N11–C33	118.7(9)
C33–N11–P3	126.3(8)
N11–C33–C34	110.2(9)
N11–C33–C35	110.1(7)
N11–C33–C36	108.2(8)
C34–C33–C36	108.6(8)
C35–C33–C34	109.4(8)
C35–C33–C36	110.3(10)
C33–C34–H34A	109.5
C33–C34–H34B	109.5
C33–C34–H34C	109.5
H34A–C34–H34B	109.5
H34A–C34–H34C	109.5
H34B–C34–H34C	109.5
C33–C35–H35A	109.5
C33–C35–H35B	109.5
C33–C35–H35C	109.5
H35A–C35–H35B	109.5
H35A–C35–H35C	109.5
H35B–C35–H35C	109.5
C33–C36–H36A	109.5
C33–C36–H36B	109.5
C33–C36–H36C	109.5
H36A–C36–H36B	109.5
H36A–C36–H36C	109.5
H36B–C36–H36C	109.5
C37–N11A–P3	109.9(16)
C33A–N11A–P3	129(3)

C33A–N11A–C37	115(3)
N11A–C33A–C34A	112(2)
N11A–C33A–C35A	110(2)
N11A–C33A–C36A	107(2)
C34A–C33A–C36A	109(2)
C35A–C33A–C34A	110(2)
C35A–C33A–C36A	108(2)
C33A–C34A–H34D	109.5
C33A–C34A–H34E	109.5
C33A–C34A–H34F	109.5
H34D–C34A–H34E	109.5
H34D–C34A–H34F	109.5
H34E–C34A–H34F	109.5
C33A–C35A–H35D	109.5
C33A–C35A–H35E	109.5
C33A–C35A–H35F	109.5
H35D–C35A–H35E	109.5
H35D–C35A–H35F	109.5
H35E–C35A–H35F	109.5
C33A–C36A–H36D	109.5
C33A–C36A–H36E	109.5
C33A–C36A–H36F	109.5
H36D–C36A–H36E	109.5
H36D–C36A–H36F	109.5
H36E–C36A–H36F	109.5

References

(1) Bruker. SAINT.
(2) Krause, L.; Herbst-Irmer, R.; Sheldrick, G. M.; Stalke, D. Comparison of Silver and Molybdenum Microfocus X-Ray Sources for Single-Crystal Structure Determination. *J. Appl. Crystallogr.* **2015**, *48* (1), 3–10. https://doi.org/10.1107/S1600576714022985.
(3) Sheldrick, G. M. SHELXT – Integrated Space-Group and Crystal-Structure Determination. *Acta Crystallogr. Sect. Found. Adv.* **2015**, *71* (1), 3–8. https://doi.org/10.1107/S2053273314026370.
(4) Sheldrick, G. M. Crystal Structure Refinement with SHELXL. *Acta Crystallogr. Sect. C Struct. Chem.* **2015**, *71* (1), 3–8. https://doi.org/10.1107/S2053229614024218.
